# Auto‐Tandem Catalysis: Pd^II^‐Catalysed Dehydrogenation/Oxidative Heck Reaction of Cyclopentane‐1,3‐diones

**DOI:** 10.1002/chem.201704442

**Published:** 2017-11-30

**Authors:** Claire J. C. Lamb, Bryan G. Nderitu, Gemma McMurdo, John M. Tobin, Filipe Vilela, Ai‐Lan Lee

**Affiliations:** ^1^ Institute of Chemical Sciences Heriot-Watt University Edinburgh EH14 4AS United Kingdom

**Keywords:** aerobic oxidation, auto-tandem catalysis, one-pot synthesis, oxidative Heck, palladium

## Abstract

A Pd^II^ catalyst system has been used to successfully catalyse two mechanistically distinct reactions in a one‐pot procedure: dehydrogenation of 2,2‐disubstituted cyclopentane‐1,3‐diones and the subsequent oxidative Heck coupling. This auto‐tandem catalytic reaction is applicable to both batch and continuous flow processes, with the latter being the first example of a tandem aerobic dehydrogenation/oxidative Heck in flow. In addition, a telescoped reaction involving enantioselective desymmetrisation of the all‐C quaternary centre was successfully achieved.

## Introduction

**Figure 1:**
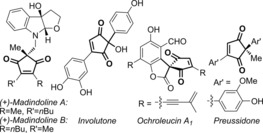
Examples of natural products containing the 2,2‐disubstituted cyclopentene‐1.3‐dione core.

**Scheme 1:**
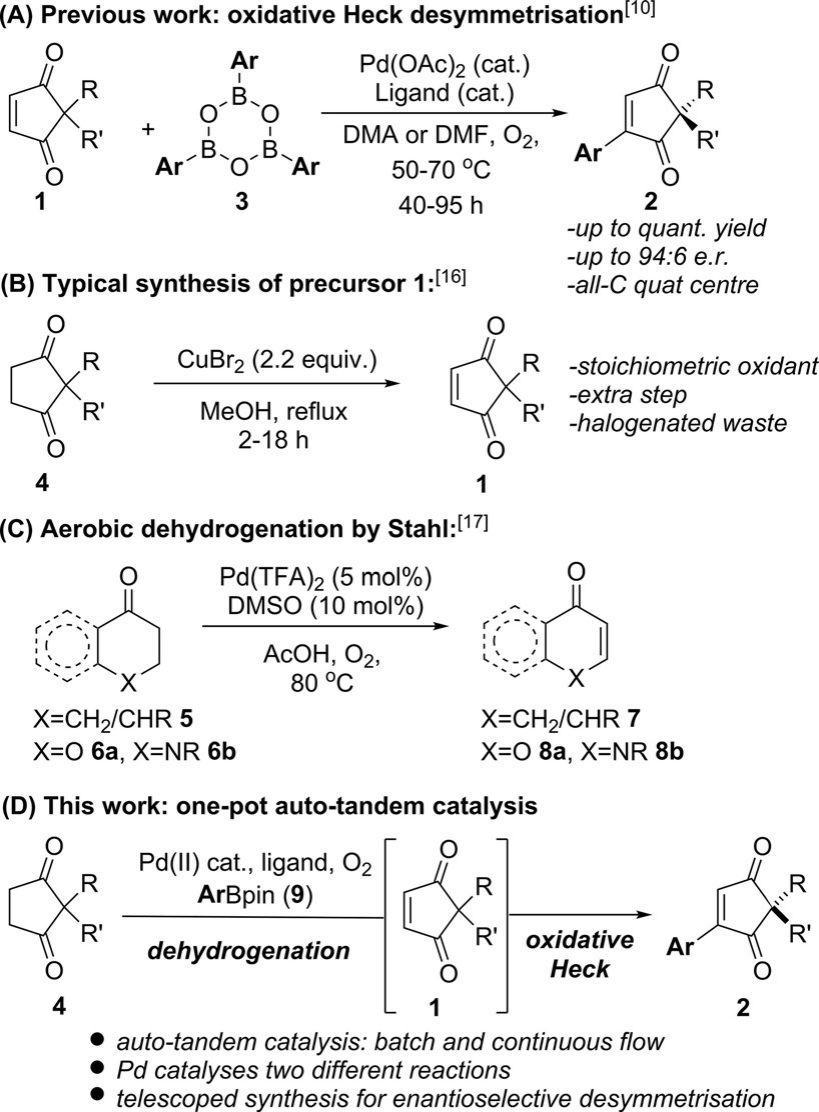
(A) Previous oxidative Heck desymmetrisation; (B) stoichiometric oxidant method towards **1**; (C) Stahl's aerobic oxidation; (D) this work.

Efficient methods to synthesise the 2,2‐disubstituted cyclopentene‐1,3‐dione core is widely sought after, as this motif is present in several biologically active compounds^1^ and natural products such as madindolines A and B,^2^ ochroleucin A_1_ 3 and similin A,^4^ as well as metabolites such as involutone^5^ and preussidone^6^ (e.g. Figure 1). Towards this end, we have recently developed a Pd^II^‐catalysed oxidative Heck,^7^, ^8^ strategy to desymmetrise the achiral precursor **1**, thereby providing an expedient way of enantioselectively desymmetrising the all‐carbon quaternary centre^9^ (**1**→**2**, Scheme 1 A).,^10^, ^11^ Alternative approaches include elegant organocatalytic methods by Mukherjee and Enders,^12^ Cu‐ or Rh‐catalysed conjugate additions to **1** by Mikami^13^ and Hayashi^14^ respectively, and silver‐catalysed desymmetrisations by Singh and Wang.^15^

In all of the aforementioned methods, however, the achiral precursor **1**, is generally accessed from cyclopentane‐1,3‐dione **4** via stoichiometric oxidation by CuBr_2_ or CuCl_2_ (Scheme 1 B).^16^ Such a procedure necessarily produces stoichiometric amounts of halogenated waste. Meanwhile, Stahl and co‐workers have developed a Pd^II^‐catalysed aerobic dehydrogenation of cyclic ketones **5** and chromanones **6** to enones **7** and chromones **8** respectively (Scheme 1 C).,^17^, ^18^ Although Stahl's conditions are very different to the oxidative Heck conditions in Scheme 1 A, and has only been applied to ketones such as **5**/**6** rather than 1,4‐diketone motifs (e.g. **4**), we were intrigued by the possibility of utilising the same Pd^II^ catalyst to carry out both the dehydrogenation of **4**→**1** and the oxidative Heck coupling **1**→**2** in a one‐pot reaction (Scheme 1 D). Such an auto‐tandem catalytic reaction,^19^ if successful, will clearly maximise the efficiency of the desymmetrisation strategy towards **1**: by reducing the number of discrete steps, maximising the efficiency of the Pd^II^ catalyst by enabling it to catalyse two mechanistically distinct reactions, and avoiding the use of stoichiometric copper salts and thereby its corresponding stoichiometric halogenated waste.

In related work, Kim and co‐workers recently demonstrated a Pd^II^‐catalysed one‐pot procedure towards flavones from chromanones **6 a**,^20^ and Hong and co‐workers synthesised functionalised cyclic enaminones and enolones from dihydroquinolinones **6 b** and chromanones **6 a,** respectively.^21^, ^22^ Nevertheless, we envisioned that the one‐pot process **4→2** that we have set out to develop would be challenging: while the one‐pot procedures mentioned above^20^, ^21^ utilise dehydrogenation **6**→**8** previously demonstrated by Stahl,^17^ the Pd^II^‐catalysed aerobic dehydrogenation of molecules with 1,4‐diketone motifs such as **4** is unprecedented. Furthermore, the one‐pot Pd‐catalysed dehydrogenation/oxidative Heck process **4→2** has the potential to be rendered enantioselective, which is once again unprecedented.

We herein disclose the successful development of the one‐pot dehydrogenation/oxidative Heck reaction of 2,2‐disubstituted cyclopentane‐1,3‐diones **4** and its corresponding substrate scope. Furthermore, the methodology can be successfully adapted for use in a continuous flow reactor, which constitutes the first example of an auto tandem catalytic aerobic dehydrogenation/oxidative Heck in flow. Finally, we demonstrate that the reaction can also be adapted for the enantioselective desymmetrisation of the all‐carbon quaternary centre in **4**, through a telescoped reaction.

## Results and Discussion

We initiated our studies by investigating the Pd^II^‐catalysed aerobic dehydrogenation of **4→1**, since this reaction has not previously been studied and would therefore require separate optimisation before the one‐pot procedure **4→2** could be attempted. Rather than Stahl's original conditions [(DMSO)_2_Pd(TFA)_2_ in AcOH (TFA=trifluoroacetate), Scheme 1 C], we chose to investigate the use of 1,10‐phenanthroline type ligands in DMF (Table 1). The reasons for this are twofold: 1,10‐phenanthroline **10** was shown to be optimal for the following oxidative Heck reaction, and would also be more suitable for adaptation to enantioselective catalysis for the following oxidative Heck step **1→2**.

**Table 1:** Selected optimisation of the Pd^II^‐catalysed aerobic dehydrogenation step.

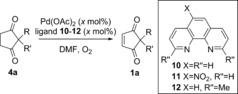					
Entry^[a]^	Ligand	*x*	Temp [°C]	Time [h]	Conv. [%]^[b]^
1	**10**	5	100	72	50
2	**11**	5	100	72	18
3	**12**	5	100	72	74
4	**10**	5	120	72	72
5	**10**	10	120	48	100 (85)^[c]^

[a] Anhydrous conditions used. [b] Conversions by ^1^H NMR analysis. [c] Isolated yield.

Firstly, the use of 5 mol % Pd(OAc)_2_ with 1,10‐phenanthroline **10** as ligand successfully produced the desired oxidation product **1**, albeit in modest conversion (50 %) after 72 h (entry 1, Table 1). Adopting 5‐nitro‐1,10‐phenanthroline **11** as the ligand resulted in an even worse conversion (18 %) under the same conditions (entry 2). Pleasingly, dmphen **12** turned out to be a good ligand for this oxidation step (entry 3), however, its use as a ligand in the subsequent oxidative Heck step **1→2** results in poor conversion. Ligand **12** is therefore unsuitable for the one‐pot reaction despite being the best ligand for dehydrogenation **4→1**. We therefore proceeded to optimise the oxidation step using 1,10‐phenanthroline **10** as ligand (entries 4–5). Increasing the reaction temperature to 120 °C provided a decent conversion of 72 % in 72 h (entry 4). Finally, increasing the catalyst and ligand loading to 10 mol % at 120 °C resulted in full conversion within 48 h, and an 85 % isolated yield of **1** (entry 5).

**Scheme 2:**
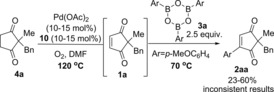
Initial attempts at the one‐pot reaction led to inconsistent results.

With these optimised conditions in hand for the first oxidation step **4**→**1**, we proceeded to investigate the one‐pot procedure by carrying out the oxidation reaction at 120 °C for approximately 30 h, followed by addition of arylboroxine **3 a** (prepared by dehydrating the corresponding arylboronic acid), and allowing the oxidative Heck reaction to proceed at 70 °C (this is the optimal temperature for the separate oxidative Heck step)^10^ for 43–93 h (Scheme 2). Following extensive optimisation (see Supporting Information), the oxidative Heck product **2 a** was successfully formed in up to 60 % yield. Frustratingly, however, the one‐pot procedure under these conditions produced very inconsistent results, with yields ranging from 23–60 % (see Supporting Information).

Whenever the yield of desired product **2 aa** was lower than expected, unreacted starting material **4 a** and intermediate **1 a** was usually present in varying amounts (see Supporting Information). At this point, we therefore surmised that the first oxidation step **4 a** to **1 a** was the cause of the inconsistent results. If the conversion of **4 a** to **1 a** is incomplete before the addition of **3 a** and cooling of the reaction to 70 °C, then no further oxidation of **4 a** to **1 a** can take place at the lower temperature. Fortunately, a quick investigation of the oxidative Heck step showed that the reaction is equally effective at higher temperatures of 100 or 120 °C (Table 2). Therefore, we proceeded to re‐investigate the full one‐pot reaction at 120 °C, in the hope that the first oxidation step will be less inconsistent at this higher temperature (Table 3). Unfortunately, this still did not lead to higher yields of desired **2 aa**.

**Table 2:** Effect of temperature on the oxidative Heck reaction.

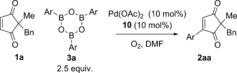				
Entry^[a]^	Temp [°C]	Time [h]	Conv. [%]^[b]^	Yield [%]^[c]^
1	70	48	100	n.d.
2	100	30	100	82
3	120	24	95	75

[a] Anhydrous conditions used. [b] Conversions by ^1^H NMR analysis. [c] Isolated yield. n.d.=not determined.

**Table 3:** Increasing temperature of the second step does not improve yield.

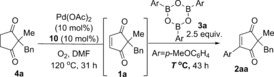				
Entry^[a]^	Temp [°C]	**2 aa** [%]^[b]^	**1 a** [%]^[b]^	**4 a** [%]^[b]^
1	100	44	–	25
2	120	44	–	–

[a] Anhydrous conditions used. [b] Determined by ^1^H NMR analysis using 1,3,5‐trimethoxybenzene as internal standard.

**Scheme 3:**
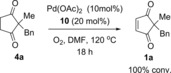
Increasing ligand:Pd ratio significantly improves reproducibility of oxidation step.

Another possible reason for the inconsistent oxidation step **4**→**1** was thought to be the stability of the active Pd^II^ catalyst at this high temperature of 120 °C: palladium black formation was occasionally observed when equal amounts of Pd(OAc)_2_ and ligand **10** were used (10 mol % respectively). This problem was successfully circumvented by addition of excess ligand (vs. Pd). Pleasingly, the addition of 20 mol % ligand **10**, while maintaining the Pd(OAc)_2_ loading at 10 mol %, resulted not only in reproducible and reliable oxidation of **4** to **2**, but also a much faster reaction time of 18 h (Scheme 3).

With much more consistent oxidation conditions in hand, the one‐pot procedure was investigated once again using the increased ligand loading (Table 4). While the oxidation of **4** to **1** was now consistently proceeding to completion (as evidenced by the absence of recovered starting material **4 a**), the oxidative Heck reaction **4** to **2** now proved problematic. Although the oxidative Heck reaction goes to completion when carried out as a separate step, it struggles to go to completion under superficially similar conditions in the one‐pot reaction. Various attempts at portion‐wise addition of boroxine at a range of temperatures failed to improve the yield of **2 aa** (Entries 1–4, Table 4). Significant amounts of side products resulting from the boroxine **9**, such as homocoupling and phenol formation, is usually observed in the one‐pot procedure.^23^ Stahl proposes that the first Pd^II^‐catalysed aerobic dehydrogenation produces hydrogen peroxide as the by‐product,^17^ and it is therefore likely that the presence of peroxide is facilitating the unwanted side‐product formation in the one‐pot reaction.^23^

**Table 4:** One‐pot reaction with increased ligand loading.

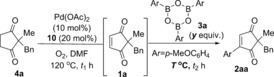					
Entry^[a]^	*T* [°C]	t_1_+t_2_ [h]	*y* [equiv]	**2 aa** [%]^[b]^	**1 a** [%]^[b]^
1	100	29+46	2+1.5^[c]^	41	21
2	120	22+73	2+1.5^[d]^	37	35
3	70	17+69	2+1.5^[e]^	40	41
4	120	20+68	2+1.5^[f,g]^	28	40

[a] Anhydrous conditions used. [b] Determined by ^1^H NMR analysis using 1,3,5‐trimethoxybenzene as internal standard. No unreacted **4 a** observed in all reactions. [c] 2nd portion added 17 h later. [d] 7 h later. [e] 20 h later. [f] 28 h later. [g] *p*‐Benzoquinone (1 equiv added).

**Scheme 4:**
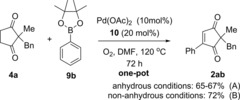
Optimised one‐pot dehydrogenation/oxidative Heck reaction.

In order to overcome this problem, the boron coupling partner was changed from arylboroxine **3** to the less reactive arylboronic esters ArBpin **9**. Pleasingly, this modification finally provided consistent and reproducible results (Scheme 4 A). The desired product **2 ab** can now be formed from **4 a** in a one‐pot procedure and consistent 65–67 % yields over the two steps (increases to 72 % under non‐anhydrous conditions, see later). Another advantage of using ArBpin **9** is that it allows for a more practical procedure, as it can be added to the reaction from the outset. This is in contrast to arylboroxine **3**, which had to be added only after oxidation of **4** to **1** was complete; yields were otherwise low due to more side‐product formation from the arylboroxine **3**.

With reproducible and optimal one‐pot conditions in hand, we set out to investigate the substrate scope of the reaction. Firstly, the aryl pinacol boronic ester scope **9** was investigated using model dione substrate **4 a** (Table 5). Unfortunately, the optimal conditions for PhBpin **9 b** shown in Scheme 4 A proved not to be general, and much lower yields were frustratingly observed when **9 a** and **9 c** were used (43 and 34 % of **2 aa** and **2 ac**, respectively, Table 5). Increasing the catalyst and ligand loading to 15 and 30 mol % led to no significant improvement (45 % and 35 % of **2 aa** and **2 ac**, respectively).

**Table 5:** Aryl pinacol boronic ester scope in the one‐pot reaction.

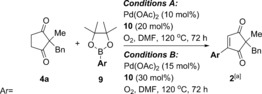
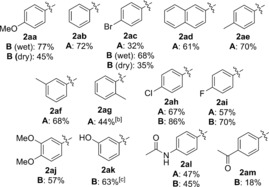

[a] Non‐anhydrous solvent and non‐dried glassware used (“wet conditions”) unless otherwise stated. Isolated yields unless otherwise stated. [b] Extra Pd(OAc)_2_ (5 mol %) added after 72 h and reaction left for a further 20 h. [c] Determined by ^1^H NMR analysis using 1,3,5‐trimethoxybenzene as internal standard.

At this point, our reasoning for this setback was that aryl pinacol boronic esters **9** are a less reactive coupling source than our original arylboroxine or arylboronic acid coupling partners.^23^, ^24^ Under strictly anhydrous conditions, it was thought that the aryl pinacol boronic esters **9** struggle to transmetallate in the absence of base, thereby resulting in low yields of **2 aa** and **2 ac**. This prompted us to attempt the reaction under “wet conditions”: non‐anhydrous solvents and non‐dried glassware, in the hope that residual water in the solvent will be sufficient to help promote transmetallation.,^23^, ^24^, ^25^, ^26^ Pleasingly, these “wet” conditions resulted in significant improvement in yields: from 45 to 77 % in the case of **3 aa** and 35 to 68 % in the case of **2 ac** (Table 5). These non‐anhydrous conditions were thus applied to the subsequent substrate scope studies (Tables 5 and 6).

**Table 6:** 2,2‐Disubstituted cyclopentane‐1,3‐dione scope in the one‐pot reaction.

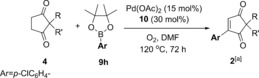
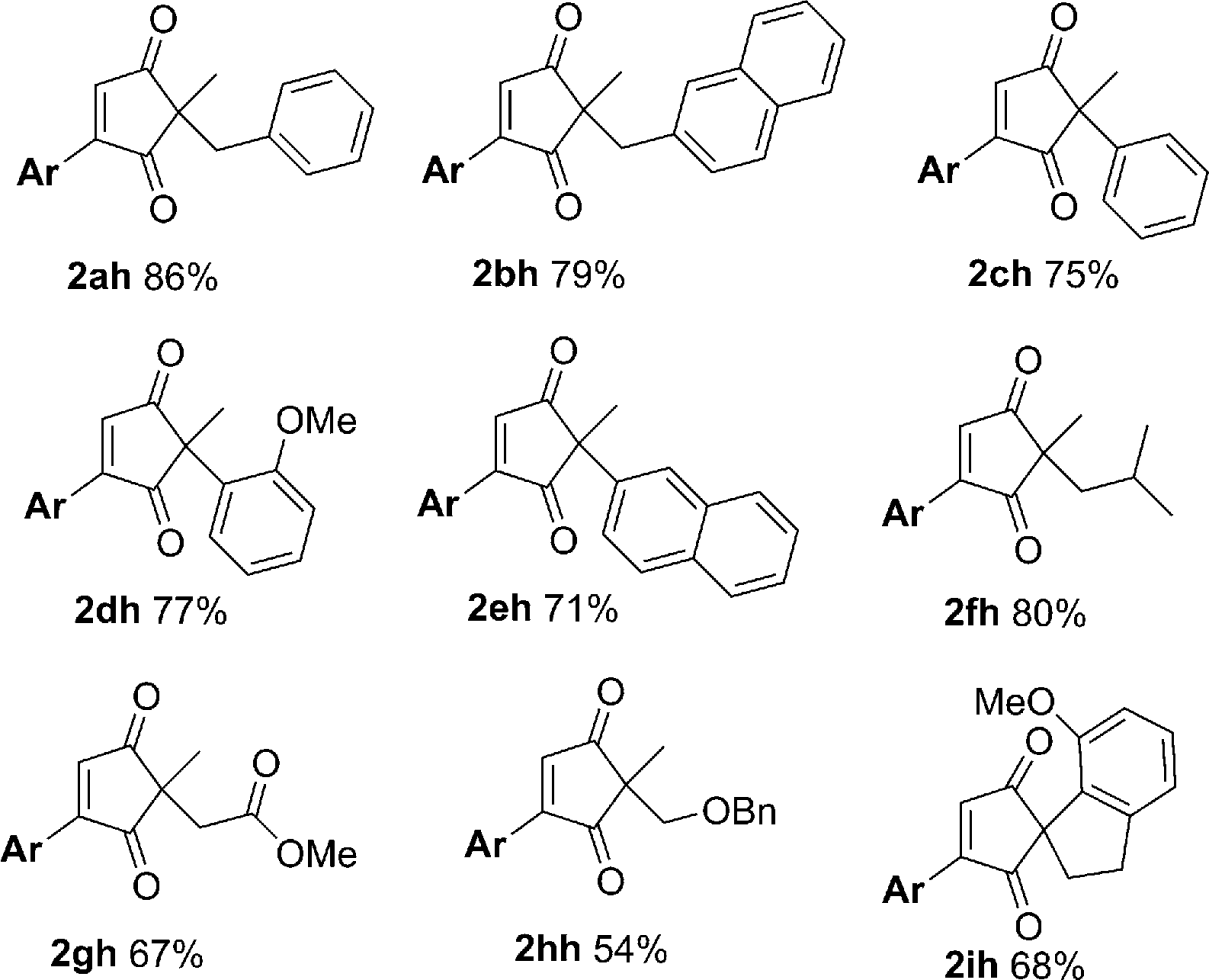

[a] Non‐anhydrous solvent and non‐dried glassware used (“wet conditions”). Isolated yields.

Results in Table 5 demonstrate that the one‐pot reaction works well for Ph‐ and naphthyl–pinacol boronic esters (72 % **2 ab** and 61 % **2 ad**). *Para*‐ and *meta*‐substitution are tolerated well (70 % **2 ae** and 68 % **2 af**), but a drop in yield to 44 % is observed for the *ortho*‐substituted tolyl **2 ag**, presumably due to steric factors. Both electron‐donating (**2 ae**–**ag**, **2 aa**, **2 aj**, **2 al**) and electron‐withdrawing **2 ac**, **2 ah**–**ai**, **2 ak**) substituents are tolerated. For a selection of these pinacol boronic esters, however, the yields were fairly moderate using the standard conditions A (e.g. **2 ac** 32 %). The use of a higher catalyst loading (15 mol %, conditions B) significantly improved the yields (e.g. **2 ac** 68 %) and conditions B were thus adopted for the less reactive coupling partners. Although this is admittedly a relatively high catalyst loading, the fact that it is used to carry out two distinct reactions in one‐pot still renders the reaction more efficient than the separate two‐step procedure, which would require 10 mol % of Pd(OAc)_2_ catalyst in each distinct step to go to completion under a similar timescale.

Finally, carbonyl containing substituents on the aryl ring results in low to moderate yields (47 % **2 al** and 18 % **2 am**) regardless of catalyst loading. Interestingly, these functional groups were tolerated well under the separate oxidative Heck conditions.^10^ These results imply that the amide and ester functionality is sensitive to the first dehydrogenation step, rather than the oxidative Heck coupling itself. Once again, it is possible that the hydrogen peroxide generated in the first step^17^ may be responsible for the lower yields of **2 al** and **2 am**.

Next, the 2,2‐disubstituted cyclopentane‐1,3‐dione (**4**) scope was investigated using aryl pinacol boronic esters **9 h** as the model coupling partner (Table 6). Replacing the Ph ring in **4 a** with a bulkier napthyl ring in **4 b** still produced the desired product **2 bh** in a good 79 % yield. Replacing the benzyl substituent in **4 a** with aromatic rings (**4 c–e**) is also tolerated, with **2 ch**, **2 dh** and **2 eh** formed in 75 %, 77 % and 71 % yields, respectively. An aryl/benzyl substituent on the cyclopentane‐1,3‐dione **4** is not necessary for good reactivity, as demonstrated by the formation of **2 fh** in a good 80 % yield. An ester group is tolerated (**2 gh**, 67 %) as is the benzyl protected alcohol (**2 hh**, 54 %). Finally, the spirocyclic **4 i** reacted smoothly to form **2 ih** in 68 % yield.

Following the successful development of the one‐pot procedure in batch, we proceeded to investigate the reaction under continuous flow. Continuous flow chemistry is an attractive alternative to traditional batch chemistry as it allows for strict regulation of specific parameters (i.e. temperature, pressure, flow rate) to control reactions which are otherwise too reactive, exothermic or hazardous for conventional use.^27^ The increased surface to volume ratio is especially useful for facilitated scale‐up of gas‐liquid reactions where a segmented flow can be beneficial for improving interface mixing.27a In our case, it should allow for more efficient O_2_ facilitated catalyst turn over. Furthermore, the scaled‐up reaction can be carried out more safely and practically under flow conditions compared to batch, especially when a flammable gaseous reagent such as oxygen is employed.^28^ We therefore sought to demonstrate this by carrying out the first auto‐tandem catalytic dehydrogenation/oxidative Heck under flow conditions.^29^

**Scheme 5:**
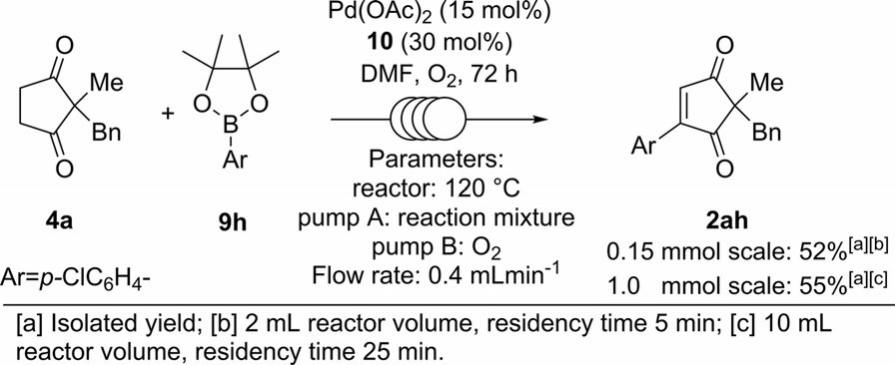
Tandem dehydrogenation/oxidative Heck under continuous flow conditions.

The continuous flow reaction was initially investigated on 0.15 mmol of substrate **4** before it was scaled up to 1.0 mmol of substrate (Scheme 5). Initial optimisation was carried out by varying the flow rate of both the reaction mixture (pump A) and oxygen (pump B, see Supporting Information). A flow rate of 0.4 mL min^−1^ and a reactor temperature of 120 °C was found to be optimal for achieving full conversion to product **2 ah** in 3 days. Pleasingly, scaling the reaction up to 1.0 mmol under the same conditions also results in full conversion, furnishing **2 ah** in 55 % yield after 3 days.

**Scheme 6:**
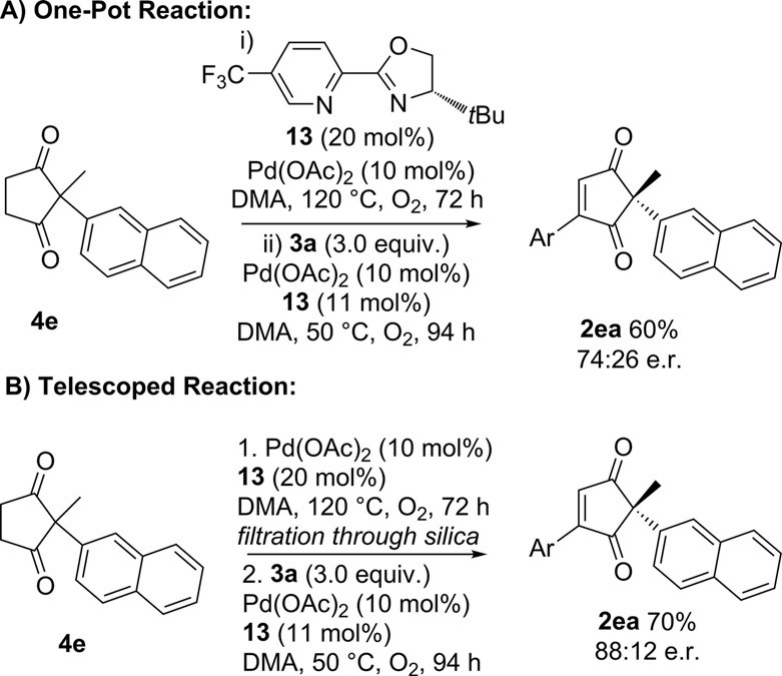
Enantioselective dehydrogenation/oxidative Heck reactions.

Finally, we aimed to extend the one‐pot procedure to the enantioselective version (Scheme 6). *t*Bu‐PyOx ligand **13** was previously used to successfully desymmetrise **1 e** via oxidative Heck coupling to produce **2 ea** in 90:10 e.r.^10^ We therefore initiated our studies by investigating whether the less reactive Pd(OAc)_2_/ligand **13** catalyst combination could oxidise **1 e**→**4 e**. The oxidation did indeed proceed to completion at 120 °C, but required 72 h using **13** as ligand (see Supporting Information) compared to 18 h using phenanthroline **10** as ligand (Scheme 3). Nevertheless, this was deemed promising enough to employ in the full on‐pot procedure (Scheme 6).

In addition to the longer reaction times, a few further modifications were required compared to the racemic procedure. Primarily, the second oxidative Heck step needs to be carried out at a lower temperature of 50 °C for optimal enantioselectivity, whereas the first dehydrogenation step requires 120 °C to proceed. Secondly, in contrast to the phenanthroline **10** ligand system used in the racemic protocol (Tables 5 and 6), switching to PyOx ligand **13** results in noticeable Pd‐black formation after dehydrogenation (i, Scheme 6 A). Therefore, a second portion of Pd/ligand **13** was added together with the coupling partner **3 a** during the one‐pot procedure (Scheme 1 A). Thirdly, the less ligating solvent dimethylacetamide (DMA) was used in order to avoid issues with competitive ligation from DMF.^30^, ^31^

Although the one‐pot reaction proceeded to a satisfactory yield of 60 %, the enantioselectivity was moderate at 74:26 e.r. (vs. 90:10 e.r. when the oxidative Heck step is carried out separately).^10^ The moderate enantioselectivity was attributed to the presence of unligated Pd formed during the aerobic dehydrogenation step in the one‐pot reaction. As a result, we proceeded to investigate the telescoped reaction instead, whereby the reaction mixture is filtered through a short plug of silica to remove any unligated Pd prior to addition of the coupling partner **3 a** (Scheme 6 B). To our delight, the telescoped reaction provided a good 70 % yield of **2 ea** over two steps, in 88:12 e.r., which is comparable to the 90:10 e.r. achieved in the separate oxidative Heck procedure.^10^

## Conclusion

In conclusion, a Pd^II^ catalyst system has been used to successfully and efficiently catalyse two mechanistically distinct reactions: dehydrogenation of 2,2‐disubstituted cyclopentane‐1,3‐diones (**4**→**1**) and the subsequent oxidative Heck coupling (**1**→**2**) in a one‐pot procedure. Such auto‐tandem catalytic reactions maximise efficiency and cut down on time, cost and waste. The development of the optimal one‐pot conditions was initially a challenging prospect, as the optimal conditions for the dehydrogenation step was not suitable for the oxidative Heck step and vice versa. Initial optimisation studies were dogged with reproducibility issues, which was thought to derive from partial decomposition of the active Pd^II^ catalyst. This problem was solved by increasing the ligand loading (vs. Pd). Secondly, the use of arylboroxine as a coupling partner was no longer optimal in the one‐pot protocol as it was susceptible to side‐product formation, thought to be facilitated by hydrogen peroxide formation from the aerobic oxidation step. Changing from arylboroxine **3** to the less reactive ArBpin **9** coupling partner solved these issues and allowed for consistent and reproducible one‐pot dehydrogenation/oxidative Heck reactions.

The first example of a continuous flow auto‐tandem catalytic dehydrogenation followed by oxidative Heck coupling was then successfully demonstrated. Finally, the one‐pot vs. telescoped dehydrogenation/enantioselective oxidative Heck was investigated using chiral PyOx ligand **13**. The one‐pot reaction provided moderate 74:26 e.r. of **2 ea** while the telescoped reaction successfully achieved a comparable e.r. to the separate oxidative Heck protocol (88:12 e.r. vs. 90:10 e.r.), in a good 70 % yield over two steps.

## Experimental Section

### Representative batch procedure

A solution of 2‐benzyl‐2‐methylcyclopentane‐1,3‐dione **4 a** (20.3 mg, 0.100 mmol, 1.0 equiv), 1,10‐phenanthroline **10** (3.6 mg, 20.0 μmol, 0.2 equiv), Pd(OAc)_2_ (2.2 mg, 9.8 μmol, 0.1 equiv) and 4,4,5,5‐tetramethyl‐2‐phenyl‐1,3,2‐dioxaborolane **9 a** (60.4 mg, 0.296 mmol, 3.0 equiv) in DMF (1 mL) was allowed to stir at room temperature for 20 minutes before being heated to 120 °C under an O_2_ atmosphere (balloon) for 71 h. Upon completion of the reaction, 2:1 Et_2_O:EtOAc (30 mL) was added and the resulting mixture washed with H_2_O (2×10 mL) and brine (10 mL). The combined organic layers were dried over MgSO_4_ and solvent was removed under reduced pressure. The resulting crude product was purified by silica gel column chromatography (25:1→20:1 petroleum ether:EtOAc) to yield 2‐benzyl‐2‐methyl‐4‐ phenylcyclopent‐4‐ene‐1,3‐dione **2 aa** (19.8 mg, 0.072 mmol, 72 %) as a yellow crystalline solid.

Full experimental procedures, characterisation for all new compounds and copies of ^1^H and ^13^C NMR spectra are provided in the Supporting Information.

## Conflict of interest

The authors declare no conflict of interest.

## Supporting information

As a service to our authors and readers, this journal provides supporting information supplied by the authors. Such materials are peer reviewed and may be re‐organized for online delivery, but are not copy‐edited or typeset. Technical support issues arising from supporting information (other than missing files) should be addressed to the authors.

Supplementary

## References

[1] Review: Z. Sevcikova , M. Pour , D. Novak , J. Ulrichova , J. Vacek , Mini Rev. Med. Chem. 2014, 14, 322–331. 24605879 10.2174/1389557514666140306130207

[2a] T. Hirose , T. Sunazuka , T. Shirahata , D. Yamamoto , Y. Harigaya , I. Kuwajima , S. Omura , Org. Lett. 2002, 4, 501–503; 11843576 10.1021/ol017058i

[2b] T. Hirose , T. Sunazuka , D. Yamamoto , E. Kaji , S. Omura , Tetrahedron Lett. 2006, 47, 6761–6764;

[2c] S. Hosokawa , K. Sekiguchi , K. Hayase , Y. Hirukawa , S. Kobayashi , Tetrahedron Lett. 2000, 41, 6435–6439;

[2d] C. C. McComas , J. B. Perales , D. L. Van Vranken , Org. Lett. 2002, 4, 2337–2340; 12098241 10.1021/ol026015e

[2e] T. Sunazuka , T. Hirose , T. Shirahata , Y. Harigaya , M. Hayashi , K. Komiyama , S. Omura , A. B. Smith III , J. Am. Chem. Soc. 2000, 122, 2122–2123;

[2f] L. Wan , M. A. Tius , Org. Lett. 2007, 9, 647–650. 17286372 10.1021/ol062919e PMC2516479

[3] B. Sontag , M. Rüth , P. Spiteller , N. Arnold , W. Steglich , M. Reichert , G. Bringmann , Eur. J. Org. Chem. 2006, 1023–1033.

[4] H. A. Weber , D. C. Swenson , J. B. Gloer , D. Malloch , Tetrahedron Lett. 1992, 33, 1157–1160.

[5a] R. Antkowiak , W. Z. Antkowiak , I. Banczyk , L. Mikolajczyk , Can. J. Chem. 2003, 81, 118–124;

[5b] L. Mikołajczyk , W. Z. Antkowiak , Heterocycles 2009, 79, 423–426;

[5c] Z.-Y. Zhou , J.-K. Liu , Nat. Prod. Rep. 2010, 27, 1531–1570. 20694228 10.1039/c004593d

[6] L. Du , J. B. King , B. H. Morrow , J. K. Shen , A. N. Miller , R. H. Cichewicz , J. Nat. Prod. 2012, 75, 1819–1823. 23046341 10.1021/np300473h PMC3483373

[7] Review on enantioselective oxidative boron Heck:10.1039/c5ob01984b 26529247

[7a] A.-L. Lee , Org. Biomol. Chem. 2016, 14, 5357; for general reviews on oxidative Heck, see:26529247 10.1039/c5ob01984b

[7b] B. Karimi , H. Behzadnia , D. Elhamifar , P. F. Akhavan , F. K. Esfahani , A. Zamani , Synthesis 2010, 1399–1427;

[7c] Y. J. Su , N. Jiao , Curr. Org. Chem. 2011, 15, 3362–3388.

[8] Selected papers on oxidative Heck:

[8a] M. M. S. Andappan , P. Nilsson , M. Larhed , Chem. Commun. 2004, 218–219; 10.1039/b311492a 14737557

[8b] C. S. Cho , S. Uemura , J. Organomet. Chem. 1994, 465, 85–92;

[8c] A. L. Gottumukkala , J. F. Teichert , D. Heijnen , N. Eisink , S. van Dijk , C. Ferrer , A. van den Hoogenband , A. J. Minnaard , J. Org. Chem. 2011, 76, 3498–3501; 21428446 10.1021/jo101942f

[8d] K. S. Yoo , C. P. Park , C. H. Yoon , S. Sakaguchi , J. O'Neill , K. W. Jung , Org. Lett. 2007, 9, 3933–3935; 17760452 10.1021/ol701584f

[8e] K. S. Yoo , C. H. Yoon , K. W. Jung , J. Am. Chem. Soc. 2006, 128, 16384–16393; 17165795 10.1021/ja063710z PMC2602842

[8f] J. H. Delcamp , A. P. Brucks , M. C. White , J. Am. Chem. Soc. 2008, 130, 11270–11271; 18671350 10.1021/ja804120r

[8g] E. W. Werner , M. S. Sigman , J. Am. Chem. Soc. 2010, 132, 13981–13983; 20858011 10.1021/ja1060998 PMC3011814

[8h] C. Zheng , D. Wang , S. S. Stahl , J. Am. Chem. Soc. 2012, 134, 16496–16499; 22998540 10.1021/ja307371w PMC3495987

[8i] S. Sakaguchi , K. S. Yoo , J. O'Neill , J. H. Lee , T. Stewart , K. W. Jung , Angew. Chem. Int. Ed. 2008, 47, 9326–9329. 10.1002/anie.200803793 18972474

[9] Recent reviews on desymmetrisation of all-carbon quaternary centres

[9a] X.-P. Zeng , Z.-Y. Cao , Y.-H. Wang , F. Zhou , J. Zhou , Chem. Rev. 2016, 116, 7330–7396; 27251100 10.1021/acs.chemrev.6b00094

[9b] K. S. Petersen , Tetrahedron Lett. 2015, 56, 6523–6535. 26834295 10.1016/j.tetlet.2015.09.134 PMC4730901

[10] S. E. Walker , C. J. C. Lamb , N. A. Beattie , P. Nikodemiak , A.-L. Lee , Chem. Commun. 2015, 51, 4089–4092. 10.1039/c5cc00407a 25665602

[11] See also:

[11a] S. E. Walker , J. Boehnke , P. E. Glen , S. Levey , L. Patrick , J. A. Jordan-Hore , A.-L. Lee , Org. Lett. 2013, 15, 1886–1889; 23578003 10.1021/ol400539h

[11b] S. E. Walker , J. A. Jordan-Hore , D. G. Johnson , S. A. Macgregor , A.-L. Lee , Angew. Chem. Int. Ed. 2014, 53, 13876–13879. 10.1002/anie.201408054 PMC4502976 25302965

[12a] M. S. Manna , S. Mukherjee , Chem. Sci. 2014, 5, 1627–1633;

[12b] M. S. Manna , S. Mukherjee , Org. Biomol. Chem. 2015, 13, 18–24; 25335849 10.1039/c4ob01649a

[12c] M. S. Manna , S. Mukherjee , J. Am. Chem. Soc. 2015, 137, 130–133; 25551319 10.1021/ja5117556

[12d] M. S. Manna , R. Sarkar , S. Mukherjee , Chem. Eur. J. 2016, 22, 14912–14919; 27603592 10.1002/chem.201602253

[12e] Y. Zhi , K. Zhao , A. Wang , U. Englert , G. Raabe , D. Enders , Adv. Synth. Catal. 2017, 359, 1867–1871.

[13] K. Aikawa , T. Okamoto , K. Mikami , J. Am. Chem. Soc. 2012, 134, 10329–10332. 22670680 10.1021/ja3032345

[14] X. Dou , Y. Lu , T. Hayashi , Angew. Chem. Int. Ed. 2016, 55, 6739–6743. 10.1002/anie.201601709 27100902

[15] For other related desymmetrisations of **1** and related cores, see:

[15a] T. Das , P. Saha , V. K. Singh , Org. Lett. 2015, 17, 5088–5091; 26439369 10.1021/acs.orglett.5b02582

[15b] L. Yao , Q. Zhu , L. Wei , Z.-F. Wang , C.-J. Wang , Angew. Chem. Int. Ed. 2016, 55, 5829–5833. 10.1002/anie.201601083 27060603

[16] W. Kreiser , A. Wiggermann , A. Krief , D. Swinnen , Tetrahedron Lett. 1996, 37, 7119–7122.

[17] T. Diao , S. S. Stahl , J. Am. Chem. Soc. 2011, 133, 14566–14569. 21851123 10.1021/ja206575j PMC3173566

[18] Review:

[18a] A. V. Iosub , S. S. Stahl , ACS Catal. 2016, 6, 8201–8213; see also:28154785 10.1021/acscatal.6b02406 PMC5279950

[18b] Y. Izawa , C. Zheng , S. S. Stahl , Angew. Chem. Int. Ed. 2013, 52, 3672–3675; 10.1002/anie.201209457 PMC3634920 23423740

[18c] D. Pun , T. N. Diao , S. S. Stahl , J. Am. Chem. Soc. 2013, 135, 8213–8221; 23662607 10.1021/ja403165u PMC3796041

[18d] T. Diao , D. Pun , S. S. Stahl , J. Am. Chem. Soc. 2013, 135, 8205–8212; 23662700 10.1021/ja4031648 PMC3795849

[18e] T. Diao , T. J. Wadzinski , S. S. Stahl , Chem. Sci. 2012, 3, 887–891. 22690316 10.1039/C1SC00724F PMC3370690

[19] For a reviews on auto-tandem catalysis, see:

[19a] J. E. Camp , Eur. J. Org. Chem. 2017, 425–433;

[19b] N. Shindoh , Y. Takemoto , K. Takasu , Chem. Eur. J. 2009, 15, 12168–12179. 19847824 10.1002/chem.200901486

[20] J. Lee , J. Yu , S. H. Son , J. Heo , T. Kim , J.-Y. An , K.-S. Inn , N.-J. Kim , Org. Biomol. Chem. 2016, 14, 777–784. 26592753 10.1039/c5ob01911g

[21] Y. Moon , D. Kwon , S. Hong , Angew. Chem. Int. Ed. 2012, 51, 11333–11336. 10.1002/anie.201206610 23038616

[22] There are one-pot examples involving dehydrogenations to phenols. For oxidative Heck followed by dehydrogenation of cyclohexenones to form *meta*-substituted phenols, see ref. [18b]; in continuous flow, but different catalyst for each step:

[22a] J. H. Park , C. Y. Park , M. J. Kim , M. U. Kim , Y. J. Kim , G.-H. Kim , C. P. Park , Org. Process Res. Dev. 2015, 19, 812–818; one-pot dehydrogenation of cyclohexanones to phenols and oxidative Heck/cyclisation to form coumarins:

[22b] D. Kim , M. Min , S. Hong , Chem. Commun. 2013, 49, 4021–4023. 10.1039/c3cc41296b 23549621

[23] A. J. J. Lennox , G. C. Lloyd-Jones , Chem. Soc. Rev. 2014, 43, 412–443. 24091429 10.1039/c3cs60197h

[24] A. Lennox , G. Lloyd-Jones , New Trends in Cross-Coupling: Theory and Applications, The Royal Society of Chemistry, London, 2015, pp. 322–354.

[25] J. W. B. Fyfe , E. Valverde , C. P. Seath , A. R. Kennedy , J. M. Redmond , N. A. Anderson , A. J. B. Watson , Chem. Eur. J. 2015, 21, 8951–8964. 25959852 10.1002/chem.201500970

[26] Addition of water proved indispensable in our recent work, see: G. Barker , S. Webster , D. G. Johnson , R. Curley , M. Andrews , P. C. Young , S. A. Macgregor , A.-L. Lee , J. Org. Chem. 2015, 80, 9807–9816. 26118859 10.1021/acs.joc.5b01041

[27a] G. Jas , A. Kirschning , Chem. Eur. J. 2003, 9, 5708–5723; 14673841 10.1002/chem.200305212

[27b] C. Wiles , P. Watts , Eur. J. Org. Chem. 2008, 1655–1671.

[28] M. B. Plutschack , B. Pieber , K. Gilmore , P. H. Seeberger , Chem. Rev. 2017, 117, 11796–11893. 28570059 10.1021/acs.chemrev.7b00183

[29] The separate steps have been carried out in continuous flow, but not the auto-tandem catalytic dehydrogenation/oxidative Heck. For aerobic dehydrogenation in flow, see ref. [17]. For oxidative Heck in continuous flow, see: L. R. Odell , J. Lindh , T. Gustafsson , M. Larhed , Eur. J. Org. Chem. 2010, 2270–2274. See also ref. [22b] for a related reverse sequence.

[30] R. Díaz-Torres , S. Alvarez , Dalton Trans. 2011, 40, 10742–10750. 21927754 10.1039/c1dt11000d

[31] Under the less reactive PyOx/Pd(OAc)_2_ catalyst system used for the enantioselective reaction, the less reactive arylpinacol boronic ester couling partners **9** no longer couple efficiently. Therefore, the more reactive coupling parter arylboroxine **3** is used.

