# Supplementary material for: Auto‐Tandem Catalysis: PdII‐Catalysed Dehydrogenation/Oxidative Heck Reaction of Cyclopentane‐1,3‐diones
Source: Chemistry. 2017 Nov 30;23(72):18282–8. doi: 10.1002/chem.201704442 (PMC5767738; doi:10.1002/chem.201704442)
Supplement: Supplementary file 1 — Supplementary [file CHEM-23-18282-s001.pdf]

CHEMISTRY
A European Journal

Supporting Information

Auto-Tandem Catalysis: Pd^{II}-Catalysed Dehydrogenation/Oxidative Heck Reaction of Cyclopentane-1,3-diones

Claire J. C. Lamb, Bryan G. Nderitu, Gemma McMurdo, John M. Tobin, Filipe Vilela,* and Ai-Lan Lee*[a]

chem_201704442_sm_miscellaneous_information.pdf

Supporting Information

Contents

General Experimental Section S2

Optimisation Studies: Racemic One-Pot Reaction S3

Optimisation Studies: Enantioselective One-Pot Reaction S6

Flow Chemistry Optimisation S8

Experimental Procedures: S9

Synthesis of 2,2-disubstituted cyclopentane-1,3-dione starting materials S9

One-Pot Dehydrogenation/Oxidative Heck Procedures S10

Pinacol Boronic Ester Scope S10

2,2-Disubstituted Cyclopentane-1,3-dione Scope S23

Enantioselective Desymmetrisation Reactions S31

Continuous Flow S35

^{1}H and ^{13}C Spectra S37

References: S60

General Experimental Section

^{1}H NMR spectra were recorded on Bruker AV 300 and AV 400 spectrometers at 300 and 400 MHz respectively and referenced to residual solvent. ^{13}C NMR spectrum were recorded using the same spectrometers at 75 and 100 MHz respectively. Chemical shifts (δ in ppm) were referenced to tetramethylsilane (TMS) or to residual solvent peaks ($CDCl_3$ at δ_H 7.26). *J* values are given in Hz and s, d, dd, t, q, p and m abbreviations correspond to singlet, doublet, doublet of doublet, triplet, quartet, pentet and multiplet. Mass spectra were obtained at the EPSRC National Mass Spectrometry Service Centre in Swansea. Infrared spectra were obtained on Perkin-Elmer Spectrum 100 FT-IR Universal ATR Sampling Accessory, deposited neat or as a chloroform solution to a diamond/ZnSe plate. Br, v str, str, w represents broad, very strong, strong and weak respectively. Reactions under flow conditions were carried out using a commercial photochemical flow reactor equipped with a thermal reactor (easy-Photochem, Vapourtec Ltd.).

Flash column chromatography was carried out using Matrix silica gel 60 from Fisher Chemicals or Flurochem and TLC was performed using Merck silica gel 60 F254 precoated sheets and visualised by UV (254 nm) or stained by the use of aqueous acidic $KMnO_4$ as appropriate. DMF was obtained dry from a solvent purification system or purchased from Sigma Aldrich (wet) without further purification. DMA was purchased from Lancaster and was used as purchased or distilled over CaH and stored over molecular sieves. All aryl boronic acids or pinacol boronic esters were purchased from Sigma-Aldrich or Fluorochem. Unless otherwise stated, where petroleum ether is used in procedures, petroleum ether 40-60 °C is the solvent used. All one-pot reactions were run under an O_2 atmosphere provided by a balloon filled with O_2 supplied by BOC.

Optimisation Studies: Racemic One-Pot Reaction

Initial investigations into the optimisation of the one-pot dehydrogenation/oxidative Heck reaction focused on using aryl boroxines as the coupling partner within the oxidative Heck reaction. The use of $Pd(OAc)_2$ (10 mol%) and 1,10-phenanthroline **10** (10 mol%) promotes the dehydrogenation reaction within 48 h (See full paper, Table 1, entry 5). In an attempt to push the oxidative Heck yield higher, portion-wise addition of catalyst and ligand was investigated (**Table S1**).

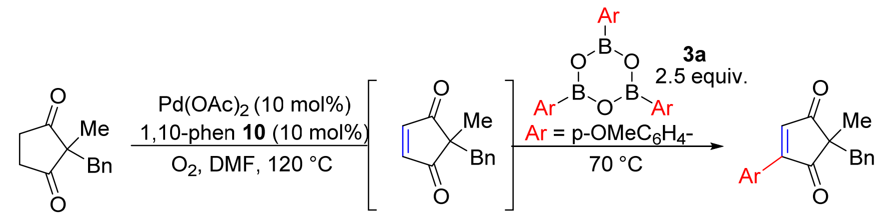

4a **1a** **2aa**

Entry[a]	$Pd(OAc)_2$	1,10-phen. **10** (mol%)	Time[c] (h)	Yield (%)[b]		
				Oxidative Heck **2aa**	Enedione **1a**	Dione **4a**
1	10	10	31.5 + 63.5	23	39	-
2	10	10	29.5 + 92.5	35	30	-
3[d]	10 + 5	10 + 5	29.5 + 65	49	8	-
4[d]	10 + 5	10 + 5	31.5 + 87.5	45	7	24
5[d, e]	10 + 5	10 + 5	31.4 + 72	40	10	-
6[d]	10 +10	10 + 10	31 + 67.5	50	7.5	6.5

[a]Reactions carried out under dry conditions; [b]Determined by ^{1}H NMR analysis using internal standard 1,3,5-trimethyoxybenzene; [c]Delayed addition of boroxine, previously dehydrated in another flask and added. Boroxine added after about 30 h and left to reaction for the second time stated; [d]Portion-wise addition of $Pd(OAc)_2$ and 1,10-phenanthroline as shown. Second portion added at the same time as boroxine; [e]Oxidative Heck reaction carried out at 100 °C.

Table S1: Investigations into portion-wise addition of $Pd(OAc)_2$ and 1,10-phenanthroline

A study into the effects of portion-wise addition of boroxine (Table S2) was carried out in parallel with the study in Table S1.

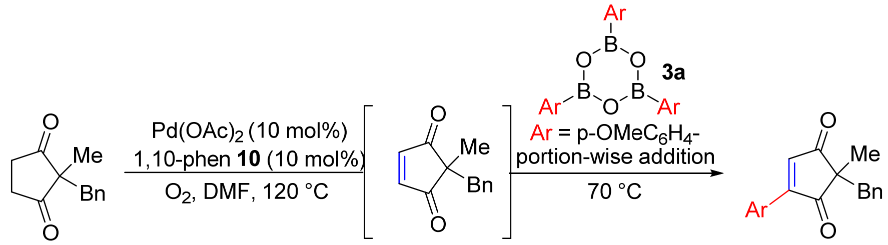

4a **1a** **2aa**

Entry[a]	$Pd(OAc)_2$	1,10-phen **10** (mol%)	Time (h)	Boroxine (equiv.)	Yield (%)[b]		
					Ox. Heck **2aa**	Endione **1a**	Dione **4a**
1	10	10	31 + 62	2 (30 h) then 1.5 (45 h)	58	-	-
2	10	10	30 + 64.5	3.5 (30 h)	50	15	7
3	10 + 5	10 + 5	30 + 64.5	3.5 (30 h)	38	4	28
4	10 + 5	10 + 5	30 + 64.5	2 (30 h) then 1.5 (45 h)	60	-	-
5[c]	10	10	24 + 43	2 (24 h) then 1.5 (43 h)	49	-	11

[a]Reactions carried out under dry conditions; [b]Determined by ^1H NMR analysis with 1,3,5-trimethoxybenzene as internal standard; [c]Double concentration 0.2 mmol **4a** in 1 mL

Table S2 : Investigations into portion-wise addition of boroxine

The issue of inconsistent dehydrogenation as discussed in the full paper resurfaced during both of the portion-wise addition studies **(Table S1 and S2)**. It was postulated that decreasing the reaction temperature for the oxidative Heck reaction was having a negative effect on the dehydrogenation step if it was not complete by the time the temperature was decreased.

Investigations into increasing the temperature of the oxidative Heck reaction:

Initially, increasing the temperature of just the oxidative Heck step was investigated. It was not known whether the oxidative Heck step would tolerate the higher catalyst and ligand loading identified as optimal for the dehydrogenation step, or whether the reaction could proceed at such elevated temperatures (Table S3). Pleasingly, the oxidative Heck step proceeds smoothly at the increased temperature of 120 °C (Table S3, entry 3).

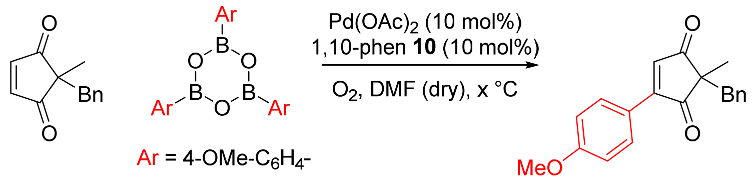

1a **3a** **2aa**

Entry	Temp (°C)	Time (h)	Conv. (%)[b]	Yield (%)[a]
1	70	48	100	-
2	100	30	100	82
3	120	24	95	75

[a]Isolated yield; [b]Conversion determined by ^1H NMR.

Table S3: Oxidative Heck reactions at increased temperature a catalyst/ligand loading

We then proceeded to study the effect of increasing the oxidative Heck reaction temperature during the full one pot process.

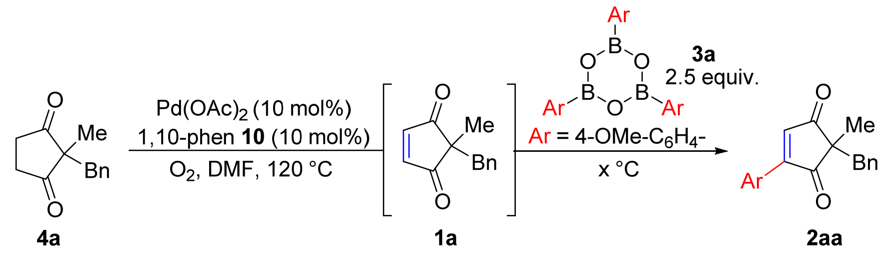

Entry[a]	Time (h)	Temp (°C)	Yield (%)[b]		
			Ox. Heck **2aa**	Endione **1a**	Dione **4a**
1	31 + 44	100	44	-	25
2	31 + 43	120	44	-	-

[a]Reactions carried out under dry conditions; [b]Determined by ^{1}H NMR analysis with 1,3,5-trimethoxybenzene as internal standard.

Table S4: Effects of increasing the temperature of the oxidative Heck portion of the one-pot reaction

All further attempts at optimising the full one-pot procedure with aryl boroxines was unsuccessful. However, turning our attention to aryl pinacol boronic esters as the coupling partner proved pivotal. For comparison, phenyl boroxine **3b** was tested under the optimised conditions for phenyl pinacol boronic ester **9b** (Scheme S1) which proved that the aryl pinacol boronic esters gave superior yields.

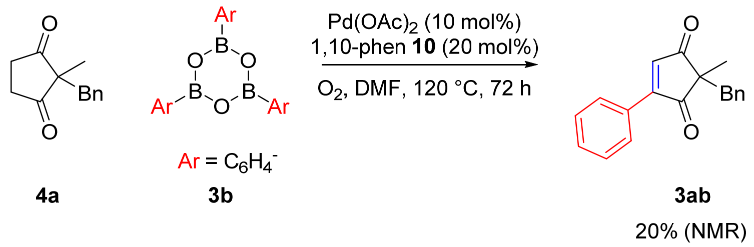

Scheme S1: Application of optimised conditions for aryl pinacol boronic esters to aryl boroxines

Our initially "optimised" pinacol boronic ester conditions turned out to be only applicable to phenyl pinacol boronic ester **9b**. To overcome this lack of substrate scope, we postulated that other pinacol boronic esters were having trouble transmetallating and that residual water in bench top solvent could be advantageous. A screen of two different pinacol boronic esters with dry and wet conditions was therefore carried out (Table S5):

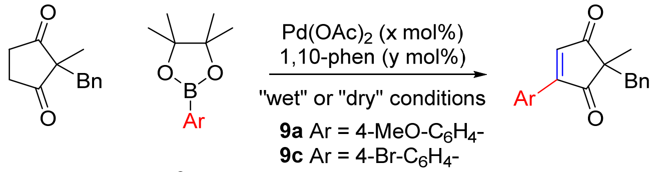

4a **9** **2a**

entry	Ar	Dry or wet Conditions[c]	$Pd(OAc)_2$ (x mol%)	Ligand (y mol%)	Product	Yield (%)
1	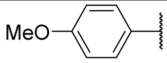	dry	10	20	**2aa**	43[a]
2	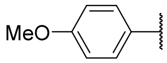	dry	15	30	**2aa**	45[a]
**3**	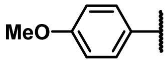	**wet**	**15**	**30**	**2aa**	**77[b]**
4	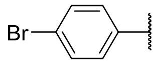	dry	10	20	**2ac**	34[b]
5	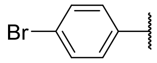	dry	15	30	**2ac**	35[b]
**6**	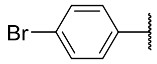	wet	10	20	**2ac**	32[b]
**7**	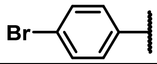	**wet**	**15**	**30**	**2ac**	**68[b]**

[a]Determined by ^{1}H NMR with 1,3,5-trimethoxybenzene as internal standard; [b]Isolated yield; [c]Dry conditions carried out with DMF from SPS, glassware dried and back filled with Ar during reagent additions. Wet conditions with bench top DMF from Sigma Aldrich with non-dried glassware.

Table S5: A screen of dry and wet conditions with electron-withdrawing and donating pinacol boronic esters

The screen highlighted the importance of residual water within the reaction solvent, significantly improving the yield when applied to electron-donating *p*-methoxylphenyl pinacol boronic ester **9a** (table S5, entry 2 vs. entry 3) and electron-withdrawing *p*-bromophenyl pincol boronic ester **9c** (Table S5, entry 5 v entry 7).

Attempts Using Stahl Conditions.

We have also investigated the reaction using Stahl's aerobic dehydrogenation conditions.[1] The reaction for the first step **4a→1a** does work using the Stahl conditions (93% NMR yield after 17 h) but these conditions are not conducive to the auto-tandem catalysis protocol as they are not suitable for the second oxidative Heck step **1→2**. For example, attempting the one-pot transformation **4a→2ah** results in only 8% desired product **2ah** under Stahl conditions, and 85% unreacted starting material:

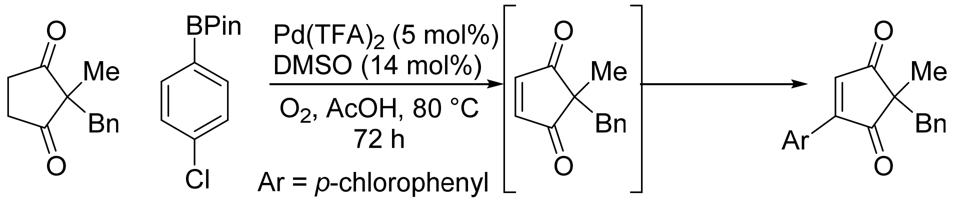

4a 85%[a] **9h** **1a** 2%[a] **2ah** 8%[a]

[a] Determined by ^{1}H NMR analysis using 1,3,5-trimethoxybenzene as internal standard

Scheme S2: Attempt at Using Stahl's Dehydrogenation Conditions for One-Pot Reaction

Optimisation Studies: Enantioselective One-Pot Reaction

In order for the one-pot desymmetrisation reaction to be carried out enantioselectively, the initial dehydrogenation reaction would also need to occur in the presence of a chiral catalyst. (*S*)-^{t}Bu pyridyloxazole [(*S*)-^{t}BuPyOx] type ligands were investigated to see if they could facilitate such a reaction.

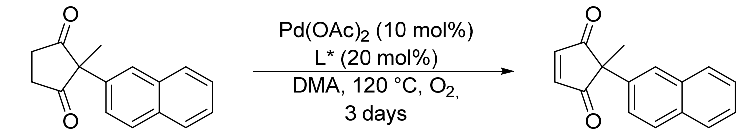

4e **1e**

Entry	L*	Conversion (%)[a]
1	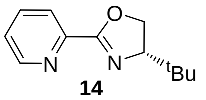 **14**	90%
2	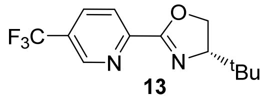 **13**	98%

[a]Conversion determined by ^{1}H NMR

Table S6: Dehydrogenation reaction using (*S*)-PyOx ligands

PyOx **13** was carried forward to the enantioselective one-pot and telescoped dehydrogenation/oxidative Heck reactions.

The main initial reason for the setup of the telescoped reaction (Scheme 6B) was to allow a direct comparison with the conditions optimised for the one-pot reaction shown in Scheme 6A. For the telescoped reaction, we also investigated using Stahl conditions for the 1st step (to avoid the need for ligand **13**), but this resulted in poor yield (35%) and e.r. (69:31 e.r.) for the overall reaction. Repeating Step 1 in Scheme 6B in the absence of ligand **13** only results in poor 11% conversion to **1e**. Racemic ligand **10** could not be used in place of **13** for the first step of the telescoped reaction as a full purification would have been required to remove all ligand after the first step.

Flow Chemistry Optimisation

Investigations into altering the flow rate of both the reaction mixture and oxygen through the flow apparatus (Figure S2) identified that a flow rate of 0.4 mL min^{-1} for both the reaction mixture and O_2 was optimal (Table S7, entry 2).

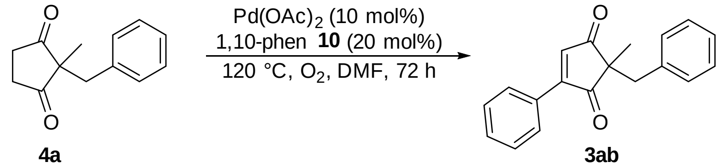

4a **3ab**

Entry	Reactor volume (mL)	Reaction volume (mL)	Flow rate: Reaction (mL/min)	Flow rate: O_2 (mL/min)	Residency time (min)	Yield (%)		
						**3ab**	**1a**	**4a**
1	2	1.5	0.1	0.1	20.0 (10.0)	33[a]	35[a]	Trace[a]
2	2	1.5	0.4	0.4	5.0 (2.5)	45[b]	20%[b]	-
3	2	1.5	0.6	0.6	3.3 (1.7)	35[a]	29[a]	-

[a] Determined by ^{1}H NMR with 1,3,5-trimethoxybenzene as internal standard; [b]isolated yield

Table S7: Flow chemistry study of one-pot dehydrogenation/oxidative Heck reaction

We also attempted to increase the temperature of the reaction, but because this is a bipasic gas-liquid reaction, the constraints of the commercial instrument being used meant that we could not approach >120 °C without significant technical problems – i.e. flash back through T-junctions (resulting in inaccurate residency times), reaction mixture not flowing through the reactor at all. This resulted in great loss of solvent and poor conversions to the final dehydrogenated coupled product.

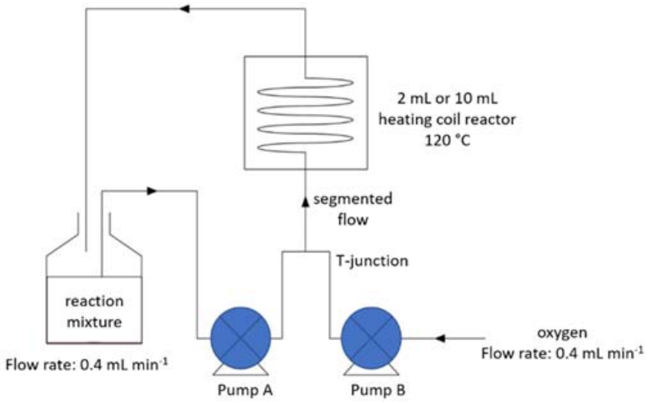

Figure S1: Diagram of the flow chemistry apparatus set up

Experimental Procedures:

Synthesis of 2,2-disubstituted cyclopentane-1,3-dione starting materials

All 2,2-disubstituted cyclopentane-1,3-diones were synthesised according to known literature procedures.

Substrates **3a**,[2] **3b**,[2] **3f**[3] and **3g**[3] were synthesised through the following known general procedure:

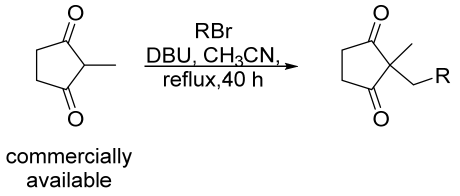

Substrates **3c**,[2] **3d**,[3] **3e**[2] and **3i**[3] were synthesised through the following known general procedure:

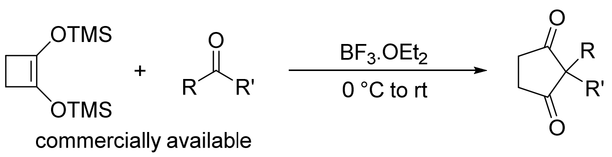

Substrate **3h**[2] was synthesised from a known literature procedure:

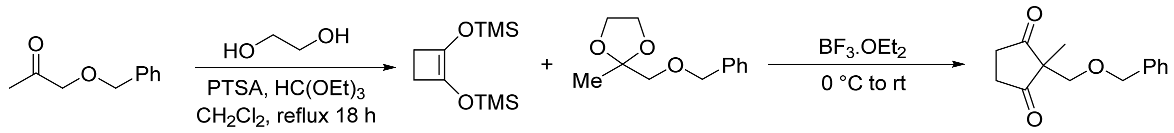

One-Pot Dehydrogenation/Oxidative Heck Procedures

Pinacol Boronic Ester Scope

2-Benzyl-4-(4-methoxyphenyl)-2-methylcyclopent-4-ene-1,3-dione (2aa)[3]

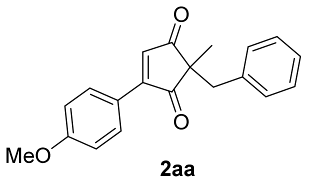

2aa

Conditions B: 2-Benzyl-2-methylcyclopentane-1,3-dione **4a** (20.4 mg, 0.101 mmol, 1.0 equiv.), 1,10-phenanthroline (5.4 mg, 29.97 µmol, 0.3 equiv.), $Pd(OAc)_2$ (3.4 mg, 15.14 µmol, 0.15 equiv.) and 2-(4-methoxyphenyl)-4,4,5,5- tetramethyl-1,3,2-dioxaborolane **9a** (70 µL, 0.305 mmol, 3.0 equiv.) were premixed and stirred in DMF (1 mL) for 20 minutes at room temperature before being heated at 120 °C in an O_2 atmosphere (balloon) for 72.5 h. Upon completion, 2:1 Et_2O:EtOAc (30 mL) was added to the reaction and the reaction mixture was washed with H_2O (3×10 mL) and brine (10 mL). The combined organic layers were dried over $MgSO_4$ and solvent was removed under reduced pressure. The resulting crude was purified by silica gel column chromatography (20:1 → 5:1 hexane:EtOAc) to yield 2-benzyl-4-(4-methoxyphenyl)-2-methylcyclopent-4-ene-1,3-dione **2aa** (23.7 mg, 0.077 mmol, 77%) as a yellow powder.

Mp: 90-92 °C (hexane/EtOAc) (literature mp: 89-91 °C);[3] R_f: 0.36 in 3:1 petroleum ether/EtOAc; ν_{max}/cm^{-1}: 3080 w, 3012 w, 2920 w, 1733 w, 1715 v w, 1684 v str, 1604 m, 1564 w, 1508 m, 1422 w, 755 w, 702 m; ^{1}H NMR (400 MHz, $CDCl_3$) δ = 7.75 (d, J = 8.9 Hz, 2H, Ar-H), 7.13 – 7.04 (m, 3H, Ar-H), 7.00 – 6.97 (m, 3H, Ar-H), 6.96 (s, 1H, =CH), 6.91 (d, J = 8.9 Hz, 2H, Ar-H), 3.84 (s, 3H, OCH_3), 3.06 (d, J = 13.4 Hz , 1H, C*H*HPh), 3.03 (d, J = 13.4 Hz, 1H, CH*H*Ph), 1.32 (s, 3H, CH_3); ^{13}C NMR (101 MHz, $CDCl_3$) δ 206.1(C), 205.5 (C), 162.4 (C), 156.2 (C), 138.8 (CH), 136.1 (C), 131.1 (CH), 129.8 (CH), 128.4 (CH), 127.0 (CH), 121.6 (C), 114.5 (CH), 55.5 (CH_3), 54.0 (C), 41.6 (CH_2), 19.8 (CH_3).

2-Benzyl-2-methyl-4-phenylcyclopent-4-ene-1,3-dione (2ab)[3]

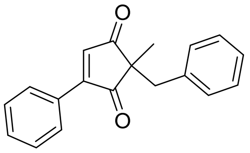

2ab

Conditions A: 2-Benzyl-2-methylcyclopentane-1,3-dione **4a** (20.3 mg, 0.100 mmol, 1.0 equiv.), 1,10-phenanthroline (3.6 mg, 19.98 μmol, 0.2 equiv.), $Pd(OAc)_2$ (2.2 mg, 9.8 μmol, 0.1 equiv.) and 4,4,5,5-tetramethyl-2-phenyl-1,3,2-dioxaborolane **9b** (60.4 mg, 0.296 mmol, 3.0 equiv.) were premixed and stirred in DMF (1 mL) for 20 minutes at room temperature before being heated to 120 °C under an O_2 atmosphere (balloon) for 71 h. Upon completion, 2:1 Et_2O:EtOAc (30 mL) was added to the reaction and the reaction mixture was washed with H_2O (2×10 mL) and brine (10 mL). The combined organic layers were dried over $MgSO_4$ and solvent was removed under reduced pressure. The resulting crude was purified by silica gel column chromatography (25:1 → 20:1 petroleum ether:EtOAC) to yield 2-benzyl-2-methyl-4- phenylcyclopent-4-ene-1,3-dione **2ab** (19.8 mg, 0.072 mmol, 72%) as a yellow crystalline solid.

Mp: 90-91 °C (petroleum ether/EtOAc) (literature mp: 91-93 °C);[3] R_f: 0.6 in 5:1 petrol ether: EtOAc; ν_{max}/ cm^{-1}:3083 w, 2920 w, 1732 w, 1684 v str, 1586 w, 1598 w, 1568 w, 760 w, 700 str; ^1H NMR (300 MHz, $CDCl_3$) δ = 7.70 – 7.65 (m, 2H, Ar-H), 7.46 – 7.36 (m, 3H, Ar-H), 7.16 – 7.07 (m, 3H, Ar-H), 7.04 (s, 1H, =CH), 7.00 – 6.95 (m, 2H, Ar-H), 3.07 (d, J = 13.1 Hz, 1H, C*H*HPh), 3.06 (d, J = 13.1 Hz, 1H, CH*H*Ph), 1.34 (s, 3H, CH_3); ^{13}C NMR (101 MHz, $CDCl_3$) δ 206.5 (C), 205.7 (C), 157.2 (C), 141.2 (CH), 135.9 (C), 131.5 (CH), 129.8 (CH), 129.13 (CH), 129.12 (CH), 128.9 (CH), 128.4 (CH), 127.1 (CH), 54.1 (C), 41.7 (CH_2), 19.7 (CH_3).

2-Benzyl-4-(4-bromophenyl)-2-methylcyclopent-4-ene-1,3-dione (2ac)

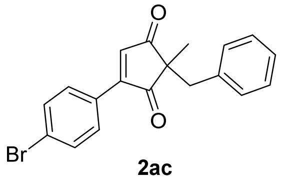

2ac

Conditions B: 2-Benzyl-2-methylcyclopentane-1,3-dione **4a** (20.3 mg, 0.100 mmol, 1.0 equiv.), 1,10-phenanthroline (5.5 mg, 30.5 µmol, 0.31 equiv.), $Pd(OAc)_2$ (3.5 mg, 15.59 µmol, 0.16 equiv.) and 2-(4-bromophenyl)-4,4,5,5-tetramethyl-1,3,2-dioxaborolane **9c** (84.9 mg, 0.300 mmol, 3.0 equiv.) were premixed and stirred in DMF (1 mL) for 20 minutes at room temperature before being heated at 120 °C in an O_2 atmosphere (balloon) for 72.5 h. Upon completion, 2:1 Et_2O:EtOAc (30 mL) was added to the reaction and the reaction mixture was washed with H_2O (2×10 mL) and brine (10 mL). The combined organic layers were dried over $MgSO_4$ and solvent was removed under reduced pressure. The resulting crude was purified by silica gel column chromatography (40:1 → 30:1 petroleum ether:EtOAC) to yield 2-benzyl-4-(4-bromophenyl)-2-methylcyclopent-4-ene-1,3-dione **2ac** (24.2 mg, 0.068 mmol, 68%) as a yellow oil.

R_f: 0.7 in 5:1 petroleum ether:EtOAc; ν_{max}/cm^{-1}: 3030 w, 2926 w, 2360, 1742 w, 1694 v str, 1589 m, 1557 w, 1485 m, 755 w, 830 m, 701 m; ^{1}H NMR (300 MHz, $CDCl_3$) δ 7.59 – 7.50 (m, 4H, Ar-H), 7.17 – 7.06 (m, 3H, Ar-H), 7.02 (s, 1H, =CH), 6.99 – 6.92 (m, 2H, Ar-H), 3.08 (d, *J* = 13.2 Hz, 1H, C*H*HPh), 3.03 (d, *J* = 13.2 Hz, 1H, CH*H*Ph) 1.33 (s, 3H, CH_3); ^{13}C NMR (75 MHz, $CDCl_3$) δ 206.3 (C), 205.5 (C), 155.9 (C), 141.2 (CH), 135.8 (C), 132.3 (CH), 130.5 (CH), 129.7 (CH), 128.5 (CH), 127.8 (C), 127.2 (CH), 126.4 (C), 54.1 (C), 41.8 (CH_2), 19.6 (CH_3); HRMS (TOF MS ASAP+) *m/z calc.* for $C_{19}H_{15}BrO_2$: 355.0334 $[M+H]^+$ found: 355.0333.

2-Benzyl-2-methyl-4-(naphthalen-2-yl)cyclopent-4-ene-1,3-dione (2ad)[3]

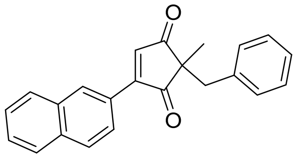

2ad

2-Benzyl-2-methylcyclopentane-1,3-dione **4a** (20.3 mg, 0.100 mmol, 1.0 equiv.), 1,10-phenanthroline (3.6 mg, 20.0 μmol, 0.2 equiv.), $Pd(OAc)_2$ (2.3 mg, 10.2 μmol, 0.10 equiv.) and 4,4,5,5-tetramethyl-2-(naphthalen-2-yl)-1,3,2-dioxaborolane **9d** (76.4 mg, 0.300 mmol, 3.0 equiv.) were premixed and stirred in DMF (1 mL) for 20 minutes at room temperature before being heated at 120 °C in an O_2 atmosphere (balloon) for 69 h. Upon completion, 2:1 Et_2O:EtOAc (30 mL) was added to the reaction and the reaction mixture was washed with H_2O (3×10 mL) and brine (10 mL). The combined organic layers were dried over $MgSO_4$ and solvent was removed under reduced pressure. To aid in purification, the crude was then mixed in EtOAc (5 mL) and sat. K_2CO_3 (5 mL) for 0.5 h to remove phenolic side products, the two phases were separated and the organic layer was washed with H_2O (2 x 10 mL). The combined organic layers were dried over $MgSO_4$ and solvent was removed by reduced pressure. The resulting crude was purified by silica gel column chromatography (25:1 → 20:1 petroleum ether:EtOAc) to yield 2-benzyl-2-methyl-4-(naphthalen-2-yl)cyclopent-4-ene-1,3-dione **2ad** (26.7 mg, 0.086 mmol, 86%) as a yellow powder.

Mp: 124-126 °C (petroleum ether:EtOAc) (literature mp: 126-128 °C);[3] R_f: 0.5 in 5:1 petroleum ether:EtOAc; ν_{max}/cm^{-1}: 3058 w, 2918 w, 1736 w, 1690 v str, 1600 m, 1581 m, 1565 m, 1495 w, 776 w, 749 m, 699 m; ^{1}H NMR (300 MHz, $CDCl_3$) δ 8.53 – 8.45 (m, 1H, Ar-H), 7.99 – 7.90 (m, 1H, Ar-H), 7.82 (m, 2H, Ar-H), 7.61 – 7.50 (m, 3H, Ar-H), 7.16 (s, 1H, =CH), 7.14 – 6.98 (m, 5H, Ar-H), 3.13 (d, *J* = 13.2 Hz, 1H, C*H*HPh), 3.07 (d, *J* = 13.2 Hz, 1H, CH*H*Ph), 1.38 (s, 3H, CH_3); ^{13}C NMR (75 MHz, $CDCl_3$) δ 206.8 (C), 205.7 (C), 156.7 (C), 141.1 (CH), 135.9 (C), 134.5 (C), 133.0 (C), 130.6 (CH), 129.8 (CH), 129.5 (CH), 128.7 (CH), 128.5 (CH), 128.2 (CH), 127.8 (CH), 127.1 (CH), 127.0 (CH), 126.3 (C), 125.0 (CH), 54.2 (C), 41.7 (CH_2), 19.8 (CH_3); HRMS (TOF MS ASAP+) *m/z calc.* for $C_{23}H_{19}O_2$: 327.1385 $[M+H]^+$; found: 327.1381.

2-Benzyl-2-methyl-4-(*p*-tolyl)cyclopent-4-ene-1,3-dione (2ae)

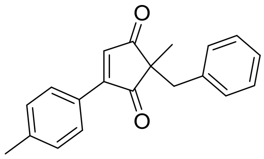

2ae

Conditions A: 2-Benzyl-2-methylcyclopentane-1,3-dione **4a** (20.4 mg, 0.101 mmol, 1.0 equiv.), 1,10-phenanthroline (3.6 mg, 0.02 μmol, 0.30 equiv.), $Pd(OAc)_2$ (2.2 mg, 0.098 μmol, 0.10 equiv.) and 4,4,5,5-Tetramethyl-2-(p-tolyl)-1,3,2-dioxaborolane **9e** (65.5 mg, 0.300 mmol, 3.0 equiv.) were premixed and stirred in DMF (1 mL) for 20 minutes at room temperature before being heated at 120 °C in an O_2 atmosphere (balloon) for 72 h. Upon completion, 2:1 Et_2O:EtOAc (30 mL) was added to the reaction and the reaction mixture was washed with H_2O (3×10 mL) and brine (10 mL). The combined organic layers were dried over $MgSO_4$ and solvent was removed under reduced pressure. The resulting crude was purified by silica gel column chromatography (30:1 petroleum ether:EtOAc) to yield 2-benzyl-2-methyl-4-(*p*-tolyl)cyclopent-4-ene-1,3-dione **2ae** (23.7 mg, 0.077 mmol, 77%) as a yellow oil.

R_f = 0.41 in 5:1 petroleum ether:EtOAc; ν_{max}/cm^{-1} = 3027, 2920, 1683, 1605, 1495, 1357, 1266, 1211, 1181, 1030, 953, 729, 695, 590, 568; ^{1}H NMR (300 MHz, $CDCl_3$) δ 7.61 (d, *J* = 8.0 Hz, 2H, Ar-H), 7.21 (d, *J* = 8.0 Hz, 2H, Ar-H), 7.15 – 7.03 (m, 3H, Ar-H), 7.00 (s, 1H, =CH), 7.00 – 6.94 (m, 2H, Ar-H), 3.07 (d, *J* = 13.3 Hz, 1H, Ar-C*H*H), 3.03 (d, *J* = 13.3 Hz, 1H, Ar-CH*H*), 2.37 (s, 3H, Ar-CH_3), 1.33 (s, 3H, CH_3); ^{13}C NMR (75 MHz, $CDCl_3$) δ = 206.6 (C), 205.7 (C), 156.9 (C), 142.1 (C), 140.2 (CH), 135.8 (C), 129.7 (CH), 129.6 (CH), 129.0 (CH), 128.3 (CH), 126.9 (CH), 126.1 (C), 53.9 (C), 41.5 (CH_2), 21.6 (CH_3), 19.6 (CH_3); HRMS (FTMS + p NSI) *m/z calc.* for $C_{20}H_{19}O_2$: 291.1380 $[M+H]^+$; found: 291.1382.

2-Benzyl-2-methyl-4-(*m*-tolyl)cyclopent-4-ene-1,3-dione (2af)

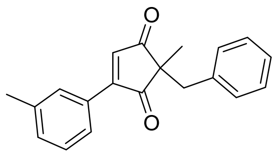

2af

Conditions A: 2-Benzyl-2-methylcyclopentane-1,3-dione **4a** (20.4 mg, 0.101 mmol, 1.0 equiv.), 1,10-phenanthroline (3.6 mg, 0.02 µmol, 0.30 equiv.), $Pd(OAc)_2$ (2.2 mg, 0.098 µmol, 0.10 equiv.) and 4,4,5,5-tetramethyl-2-(m-tolyl)-1,3,2-dioxaborolane **9f** (65.3 mg, 0.300 mmol, 3.0 equiv.) were premixed and stirred in DMF (1 mL) for 20 minutes at room temperature before being heated at 120 °C in an O_2 atmosphere (balloon) for 72 h. Upon completion, 2:1 Et_2O:EtOAc (30 mL) was added to the reaction and the reaction mixture was washed with H_2O (3×10 mL) and brine (10 mL). The combined organic layers were dried over $MgSO_4$ and solvent was removed under reduced pressure. The resulting crude was purified by silica gel column chromatography (30:1 petroleum ether:EtOAc) to yield 2-benzyl-2-methyl-4-(*m*-tolyl)cyclopent-4-ene-1,3-dione **2af** (19.8 mg, 0.068 mmol, 68%) as a yellow oil.

R_f = 0.70 in 5:1 petroleum ether:EtOAc; ν_{max}/cm^{-1} = 3069, 2968, 2922, 1734, 1694,1609, 1581, 1487, 1450, 1377, 1324, 1377, 1324, 1307, 1235, 1135, 1120, 1097,1071, 1603, 919, 907, 885, 825, 814, 801, 723, 689,655, 590 565; ^{1}H NMR (300 MHz, $CDCl_3$) δ 7.50 – 7.44 (m, 2H, Ar-H), 7.32 – 7.23 (m, 3H, Ar-H), 7.16 – 7.05 (m, 3H, Ar-H), 7.02 (s, 1H, =CH), 7.01 – 6.95 (m, 2H, Ar-H), 3.08 (d, *J* = 13.3 Hz, 1H, PhC*H*H), 3.03 (d, *J* = 13.3 Hz, 1H, PhCH*H*), 2.37 (s, 3H, $ArCH_3$), 1.33 (s, 3H, CH_3); ^{13}C NMR (75 MHz, $CDCl_3$) δ = 206.44 (C), 205.69 (C), 157.29 (C), 141.04 (CH), 138.53 (C), 135.81 (C), 132.2 (CH), 129.7 (CH), 129.5 (CH), 128.9 (C), 128.7 (CH), 128.3 (CH), 126.9 (CH), 126.2 (CH), 53.92 (C), 41.5 (CH_2), 21.4 (CH_3), 19.7 (CH_3); HRMS (FTMS + p NSI) *m/z calc.* for $C_{20}H_{19}O_2$: 291.1380 $[M+H]^+$; found: 291.1382.

2-Benzyl-2-methyl-4-(*o*-tolyl)cyclopent-4-ene-1,3-dione (2ag)[3]

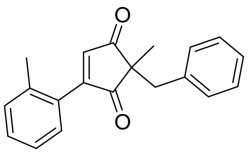

2ag

Conditions A: 2-Benzyl-2-methylcyclopentane-1,3-dione **4a** (20.4 mg, 0.101 mmol, 1.0 equiv.), 1,10-phenanthroline (3.6 mg, 0.02 µmol, 0.30 equiv.), $Pd(OAc)_2$ (2.2 mg, 0.098 µmol, 0.10 equiv.) and 4,4,5,5-tetramethyl-2-(o-tolyl)-1,3,2-dioxaborolane **9g** (66 µL, 0.300 mmol, 3.0 equiv.) were premixed and stirred in DMF (1 mL) for 20 minutes at room temperature before being heated at 120 °C in an O_2 atmosphere (balloon) for 72 h. A further portion of $Pd(OAc)_2$ (1.1 mg, 0.049 µmol, 0.05 equiv.) was added and the reaction was left to stir for a further 24 h. Upon completion, 2:1 Et_2O:EtOAc (30 mL) was added to the reaction and the reaction mixture was washed with H_2O (3×10 mL) and brine (10 mL). The combined organic layers were dried over $MgSO_4$ and solvent was removed under reduced pressure. The resulting crude was purified by silica gel column chromatography (30:1 petroleum ether:EtOAc) to yield 2-benzyl-2-methyl-4-(*o*-tolyl)cyclopent-4-ene-1,3-dione **2ag** (12.8 mg, 0.044 mmol, 44%) as a yellow oil.

R_f = 0.65 in 5:1 petroleum ether:EtOAc; ν_{max}/cm^{-1} = 3029, 2927,1745, 1697, 1635, 1605, 1495, 1452, 1373, 1329, 1303, 1237, 1203,1137, 1092, 1071, 1055, 1043, 902, 859, 803, 790, 750, 726, 700, 667, 640, 593, 562; ^1H NMR (300 MHz, $CDCl_3$) δ 7.32 – 7.26 (m, 1H, Ar-H), 7.23 – 7.12 (m, 5H, Ar-H), 7.01 – 6.95 (m, 3H, Ar-H), 6.88 (s, 1H, =CH), 3.11 (d, J = 13.0 Hz, 1H, Ph-CH*H*), 3.05 (d, J = 13.0 Hz, 1H, Ph-C*H*H), 1.91 (s, 3H, Ph-CH_3), 1.36 (s, 3H, CH_3); ^{13}C NMR (75 MHz, $CDCl_3$) δ = 206.2 (C), 205.9 (C), 157.3 (C), 144.9 (CH), 136.5 (C), 135.8 (C), 130.8 (CH), 130.1 (CH), 129.8 (CH), 129.3 (C), 128.7 (CH), 128.4 (CH), 127.1 (CH), 125.6 (CH), 52.9 (C), 41.5 (CH_2), 20.1 (CH_3), 19.6 (CH_3).

2-Benzyl-4-(4-chlorophenyl)-2-methylcyclopent-4-ene-1,3-dione (2ah)

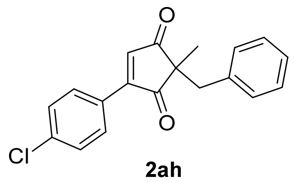

2ah

Conditions B: 2-Benzyl-2-methylcyclopentane-1,3-dione **4a** (20.3 mg, 0.100 mmol, 1.0 equiv.), 1,10-phenanthroline (5.5 mg, 30.5 μmol, 0.31 equiv.), $Pd(OAc)_2$ (3.5 mg, 15.59 μmol, 0.16 equiv.) and 2-(4-chlorophenyl)-4,4,5,5-tetramethyl- 1,3,2-dioxaborolane **9h** (71.5 mg, 0.300 mmol, 3.0 equiv.) were premixed and stirred in DMF (1 mL) for 20 minutes at room temperature before being heated at 120 °C in an O_2 atmosphere (balloon) for 69 h. Upon completion, 2:1 Et_2O:EtOAc (30 mL) was added to the reaction and the reaction mixture was washed with H_2O (3×10 mL) and brine (10 mL). The combined organic layers were dried over $MgSO_4$ and solvent was removed under reduced pressure. The resulting crude was purified by silica gel column chromatography (30:1 → 20:1 petroleum ether:EtOAC) to yield 2-benzyl-4-(4-chlorophenyl)- 2-methylcyclopent-4-ene-1,3-dione **2ah** (26.7 mg, 0.086 mmol, 86%) as a yellow oil.

R_f: 0.48 in 5:1 petroleum ether/EtOAc; ν_{max}/cm^{-1}:2925 w, 1741 w, 1694 v str, 1595 m, 1559 w, 1489 m, 834 m, 756 m, 701 m; ^1H NMR (300 MHz, $CDCl_3$) δ 7.67 – 7.60 (d, J = 8.6 Hz, 2H, Ar-H), 7.41 – 7.34 (d, J = 8.6 Hz, 2H, Ar-H), 7.13 – 7.06 (m, 3H, Ar-H), 7.02 (s, 1H, =CH), 6.98 – 6.92 (m, 3H, Ar-H), 3.06 (apparent s, 2H, CH_2Ph), 1.33 (s, 2H, CH_3); ^{13}C NMR (75 MHz, $CDCl_3$) δ 206.3 (C), 205.5 (C), 155.8 (C), 141.3 (CH), 137.9 (C), 135.8 (C), 130.4 (CH), 129.7 (CH), 129.3 (CH), 128.5 (CH), 127.4 (C), 127.2 (CH), 54.1 (C), 41.8 (CH_2), 19.6 (CH_3); HRMS (TOF MS ASAP+) *m/z* calc. for $C_{19}H_{15}ClO_2$: 3110839 $[M+H]^+$ found: 311.0842.

2-Benzyl-4-(4-fluorophenyl)-2-methylcyclopent-4-ene-1,3-dione (2ai)

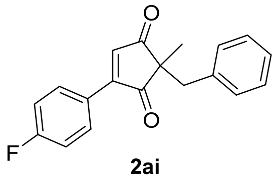

2ai

Conditions B: 2-Benzyl-2-methylcyclopentane-1,3-dione **4a** (20.3 mg, 0.100 mmol, 1.0 equiv.), 1,10-phenanthroline (5.5 mg, 30.5 μmol, 0.3 equiv.), $Pd(OAc)_2$ (3.5 mg, 15.6 μmol, 0.16 equiv.) and 2-(4-fluorophenyl)-4,4,5,5-tetramethyl- 1,3,2-dioxaborolane **9i** (67.8 mg, 0.305 mmol, 3.0 equiv.) were premixed and stirred in DMF (1 mL) for 20 minutes at room temperature before being heated at 120 °C in an O_2 atmosphere (balloon) for 69 h. Upon completion, 2:1 Et_2O:EtOAc (30 mL) was added to the reaction and the reaction mixture was washed with H_2O (3×10 mL) and brine (10 mL). The combined organic layers were dried over $MgSO_4$ and solvent was removed under reduced pressure. The resulting crude was purified by silica gel column chromatography (20:1 hexane:EtOAc) to yield 2-benzyl-4-(4-fluorophenyl)-2-methylcyclopent-4-ene-1,3-dione **2ai** (20.6 mg, 70.0 mmol, 70%) as a yellow oil.

R_f: 0.7 in 3:1 petrol ether:EtOAc; ν_{max}/cm^{-1}: 2928 w, 1742 w, 1694 v str, 1601 m, 1505 str, 1452 w, 841 str, 753 m, 701 m; ^{1}H NMR (300 MHz, $CDCl_3$) δ 7.76 – 7.68 (m, 2H, Ar-H), 7.15 – 7.04 (m, 5H, Ar-H), 7.00 (s, 1H, =CH), 6.99 – 6.93 (m, 2H, Ar-H), 3.06 (apparent s, 2H, CH_2Ph), 1.33 (s, 3H, CH_3); ^{13}C NMR (75 MHz, $CDCl_3$) δ 206.5 (C), 205.4 (C), 164.7 (d, ^1J = 253.6 Hz, C), 155.8 (C), 140.7 (CH), 135.9 (C), 131.40 (d, ^3J = 8.9 Hz, CH), 129.8 (CH), 128.5 (CH), 127.2 (CH), 125.3 (d, ^4J = 3.4 Hz, C), 116.2 (d, ^2J = 21.6 Hz, CH), 54.1 (C), 41.8 (CH_2), 19.7 (CH_3); ^{19}F NMR (376 MHz, $CDCl_3$) δ -107.03 – -107.21 (m, Ar-F); HRMS (TOF MS ASAP+) *m/z calc.* for $C_{19}H_{15}FO_2$: 295.1134 $[M+H]^+$; found: 295.1129.

2-Benzyl-4-(3,4-dimethoxyphenyl)-2-methylcyclopent-4-ene-1,3-dione (2aj)[3]

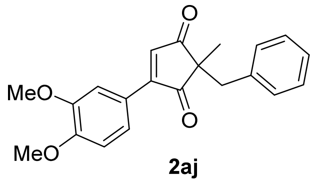

2aj

Conditions B: 2-Benzyl-2-methylcyclopentane-1,3-dione **4a** (20.3 mg, 0.100 mmol, 1.0 equiv.), 1,10-phenanthroline (5.4 mg, 30.0 µmol, 0.3 equiv.), $Pd(OAc)_2$ (3.4 mg, 15.5 µmol, 0.16 equiv.) and 2-(3,4-dimethoxyphenyl)-4,4,5,5- tetramethyl-1,3,2-dioxaborolane **9j** (80.2 mg, 0.304 mmol, 3.0 equiv.) were premixed and stirred in DMF (1 mL) for 20 minutes at room temperature before being heated at 120 °C in an O_2 atmosphere (balloon) for 71 h. Upon completion, 2:1 Et_2O:EtOAc (30 mL) was added to the reaction and the reaction mixture was washed with H_2O (3×10 mL) and brine (10 mL). The combined organic layers were dried over $MgSO_4$ and solvent was removed under reduced pressure. The resulting crude was purified by silica gel column chromatography (4:1→2:1 hexane:EtOAc) to yield 2-benzyl-4-(3,4-dimethoxyphenyl)-2-methylcyclopent-4-ene-1,3-dione **2aj** (19.1 mg, 56.8 mmol, 57%) as a yellow oil.

R_f: 0.3 in 3:1 petrol ether:EtOAc; ^{1}H NMR (300 MHz, $CDCl_3$) δ 7.44 (dd, *J* = 8.5, 2.0 Hz, 1H, Ar-H), 7.31 (d, *J* = 2.0 Hz, 1H, Ar-H), 7.15 – 7.06 (m, 3H, Ar-H), 7.01 – 6.94 (m, 3H, ArH and =CH), 6.88 (d, *J* = 8.5 Hz, 1H, Ar-H), 3.92 (s, 3H, OCH_3), 3.90 (s, 3H, OCH_3), 3.05 (apparent s, 2H, CH_2Ph), 1.33 (s, 3H, CH_3); ^{13}C NMR (75 MHz, $CDCl_3$) δ 207.1 (C), 205.4 (C), 156.2 (C), 152.2 (C), 149.3 (C), 139.0 (CH), 136.1 (C), 129.9 (CH), 128.4 (CH), 127.1 (CH), 123.4 (CH), 121.9 (C), 111.9 (CH), 111.3 (CH), 56.2 (CH_3), 54.2 (C), 41.7 (CH_2), 19.9 (CH_3); HRMS (FTMS + p NSI) *m/z calc.* for $C_{21}H_{21}O_4$: 377.1434 $[M+H]^+$; found: 377.1438

2-Benzyl-4-(3-hydroxyphenyl)-2-methylcyclopent-4-ene-1,3-dione (2ak)

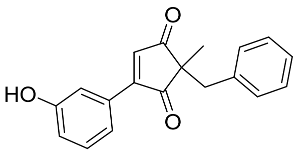

2ak

Conditions B: 2-Benzyl-2-methylcyclopentane-1,3-dione **4a** (20.4 mg, 0.101 mmol, 1.0 equiv.), 1,10-phenanthroline (5.4 mg, 30.0 μmol, 0.3 equiv.), $Pd(OAc)_2$ (3.4 mg, 15.1 μmol, 0.15 equiv.) and 2-(3,4-dimethoxyphenyl)-4,4,5,5-tetramethyl- 1,3,2-dioxaborolane **9k** (66.1 mg, 0.300 mmol, 2.98 equiv.) were premixed and stirred in DMF (1 mL) for 20 minutes at room temperature before being heated at 120 °C in an O_2 atmosphere (balloon) for 71 h. Upon completion, 2:1 Et_2O:EtOAc (30 mL) was added to the reaction and the reaction mixture was washed with H_2O (3×10 mL) and brine (10 mL). The combined organic layers were dried over $MgSO_4$ and solvent was removed under reduced pressure. The resulting crude was purified by silica gel column chromatography (10:1→5:1 hexane:EtOAc + 1% toluene) to yield 2- benzyl-4-(3-hydroxyphenyl)-2-methylcyclopent-4-ene-1,3-dione **2ak** (22.3 mg, 76.3 mmol, 78% yield at ~80% purity, NMR yield 63% with internal standard 1,3,5-trimethoxybenzene) as a yellow oil. A small portion of the material was successfully purified for characterisation (8.5 mg).

R_f: 0.24 in 3:1 petrol ether:EtOAc; ν_{max}/cm^{-1}: 3280 br, 3076 w, 2919 w, 1729 m, 1675 v str, 1588 m, 1571 m, 1489 m, 884 m, 795 str, 700 m; ^{1}H NMR (300 MHz, $CDCl_3$) δ 7.30 – 7.26 (m, 2H, Ar-H and $CDCl_3$), 7.22 (dt, *J* = 7.8, 1.3 Hz, 1H, Ar-H), 7.15 – 7.07 (m, 3H, Ar-H), 7.03 (s, 1H, =CH), 6.98 – 6.91 (m, 3H, Ar-H), 5.24 (s, 1H, OH), 3.10 (d, *J* = 13.4 Hz, 1H, C*H*HPh), 3.06 (d, *J* = 13.1 Hz, 1H, CH*H*Ph), 1.34 (broad s, 3H, CH_3); ^{13}C NMR (75 MHz, $CDCl_3$) δ 206.5 (C), 205.7 (C), 156.7 (C), 155.9 (C), 141.5 (CH), 135.9 (C), 130.4 (C), 130.3 (CH), 129.8 (CH), 128.5 (CH), 127.2 (CH), 121.7 (CH), 118.7 (CH), 115.9 (CH), 54.2 (C), 41.7 (CH_2), 19.7 (CH_3); HRMS (FTMS + p NSI) *m/z calc.* for $C_{19}H_{17}O_3$: 293.1172 $[M+H]^+$; found: 293.1176.

N-(4-(4-Benzyl-4-methyl-3,5-dioxocyclopent-1-en-1-yl)phenyl)acetamide (2al)[3]

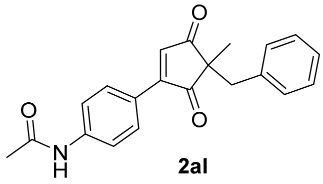

2al

Conditions A: 2-Benzyl-2-methylcyclopentane-1,3-dione **4a** (20.3 mg, 0.100 mmol, 1.0 equiv.), 1,10-phenanthroline (3.6 mg, 19.98 μmol, 0.2 equiv.), $Pd(OAc)_2$ (2.2 mg, 9.8 μmol, 0.10 equiv.) and N-(4-(4,4,5,5-tetramethyl-1,3,2-dioxaborolan-2-yl)phenyl) acetamide **9l** (78.0 mg, 0.299 mmol, 2.9 equiv.) were premixed and stirred in DMF (1 mL) for 20 minutes at room temperature before being heated at 120 °C in an O_2 atmosphere (balloon) for 72.5 h. Upon completion, 2:1 Et_2O:EtOAc (30 mL) was added to the reaction and the reaction mixture was washed with H_2O (2×10 mL) and brine (10 mL). The combined organic layers were dried over $MgSO_4$ and solvent was removed under reduced pressure. The resulting crude was purified by silica gel column chromatography (5:1 → 2:1 Hexanes:EtOAc) to yield N-(4-(4-benzyl-4-methyl-3,5-dioxocyclopent-1-en-1-yl)phenyl)acetamide **2al** (15.6 mg, 0.047 mmol, 47%) as a yellow oil.

R_f: 0.27 in 1;1 petroleum ether:EtOAc; ν_{max}/cm^{-1}: 3306 br, 3031 w, 2925 w, 1739 w, 1689 v str, 1592 str, 1508 str, 1410 m, 755 w, 700 m; ^1H NMR (400 MHz, $CDCl_3$) δ 7.72 (d, J = 8.3 Hz, 2H, Ar-H), 7.56 (d, J = 8.3 Hz, 2H, Ar-H), 7.43 (s, 1H, NH), 7.13 – 7.05 (m, 3H, Ar-H), 6.99 (s, 1H, =CH), 6.98 – 6.92 (m, 2H, Ar-H), 3.08 (d, J = 13.7 Hz, 1H, C*H*HPh), 3.01 (d, J, = 13.7 Hz, 1H, CH*H*Ph), 2.19 (s, 3H, CH_3C=O), 1.32 (s, 3H, CH_3); ^{13}C NMR (75 MHz, $CDCl_3$) δ 206.8 (C), 205.6 (C), 168.6 (C), 156.0 (C), 140.9 (C), 139.9 (CH), 135.9 (C), 130.3 (CH), 129.8 (CH), 128.4 (CH), 127.1 (CH), 124.6 (C), 119.5 (CH), 54.1 (C), 41.7 (CH_2), 24.9 (CH_3), 19.7 (CH_3); HRMS (NSI) *m/z* calc. for $C_{21}H_{19}NO_3$: 334.1442 $[M+H]^+$ found: 334.1438.

4-(4-Acetylphenyl)-2-benzyl-2-methylcyclopent-4-ene-1,3-dione (2am)[3]

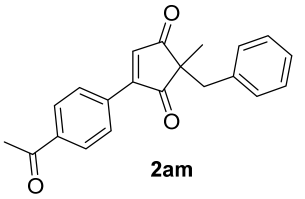

2am

Conditions B: 2-Benzyl-2-methylcyclopentane-1,3-dione **4a** (20.3 mg, 0.100 mmol, 1.0 equiv.), 1,10-phenanthroline (5.4 mg, 29.97 µmol, 0.30 equiv.), $Pd(OAc)_2$ (3.4 mg, 15.15 µmol, 0.15 equiv.) and 1-(4-(4,4,5,5-tetramethyl-1,3,2-dioxaborolan-2- yl)phenyl)ethan-1-one **9m** (74.2 mg, 0.300 mmol, 3.0 equiv.) were premixed and stirred in DMF (1 mL) for 20 minutes at room temperature before being heated at 120 °C in an O_2 atmosphere (balloon) for 72.5 h. Upon completion, 2:1 Et_2O:EtOAc (30 mL) was added to the reaction and the reaction mixture was washed with H_2O (3×10 mL) and brine (10 mL). The combined organic layers were dried over $MgSO_4$ and solvent was removed under reduced pressure. The resulting crude was stirred in EtOAc (5mL) and HCl (0.1 M, 5 mL) overnight. The aqueous layer was washed with EtOAC (10 mL) and the combined organic layers were then stirred in sat. K_2CO_3 (15 mL) for 30 minutes at 30 °C. The above acid followed by base wash was carried out to remove any co-eluting pinacol boronic ester and corresponding phenol. The aqueous layer was washed with EtOAc and the organic layer was dried over $MgSO_4$ and solvent was removed under reduced pressure. The resulting crude was purified by silica gel column chromatography (5:1 → 3:1 hexanes:EtOAc) to yield 4-(4-acetylphenyl)-2-benzyl-2-methylcyclopent-4-ene-1,3-dione **2am** (10.7 mg, 0.018 mmol, 18%) as a yellow oil.

R_f: 0.36 in 5:1 petroleum ether/EtOAc; ^{1}H NMR (300 MHz, $CDCl_3$) δ 7.93 – 7.86 (d, J = 8.2 Hz, 2H, Ar-H), 7.70 – 7.64 (d, J = 8.2 Hz 2H, Ar-H), 7.06 – 7.00 (m, 3H, Ar-H), 6.95 – 6.84 (m, 2H, Ar-H), 3.00 (apparent s, 2H, CH_2Ph), 2.54 (s, 3H, C(O)CH_3), 1.28 (s, 3H, CH_3); ^{13}C NMR (75 MHz, $CDCl_3$) δ 206.0 (C), 205.5 (C), 197.4 (C), 156.1 (C), 142.5 (CH), 138.9 (C), 135.8 (C), 133.2 (C), 129.8 (CH), 129.3 (CH), 128.6 (CH), 128.5 (CH), 127.3 (CH), 54.2 (C), 42.0 (CH_2), 26.8 (CH_3), 19.5 (CH_3); HRMS (FTMS p NSI) *m/z* calc. for $C_{21}H_{19}O_3$: 319.1329 $[M+H]^+$ found: 319.1333.

2,2-Disubstituted Cyclopentane-1,3-dione Scope

Conditions B were used for the 2,2-disusbstituted cyclopentane-1,3-dione scope.

4-(4-Chlorophenyl)-2-methyl-2-(naphthalen-2-ylmethyl)cyclopent-4-ene-1,3-dione (2bh)

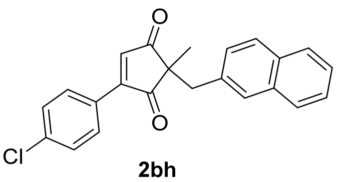

2bh

Conditions B: 2-methyl-2-(naphthalen-2-ylmethyl)cyclopentane-1,3-dione **1b** (25.2mg, 0.100 mmol, 1.0 equiv.), 1,10-phenanthroline (5.3 mg, 29.41 μmol, 0.3 equiv.), $Pd(OAc)_2$ (3.4 mg, 15.14 μmol, 0.15 equiv.) and 2-(4-chlorophenyl)-4,4,5,5-tetramethyl-1,3,2-dioxaborolane **9h** (71.7 mg, 0.301 mmol, 3.0 equiv.) were premixed and stirred in DMF (1 mL) for 20 minutes at room temperature before being heated at 120 °C in an O_2 atmosphere (balloon) for 72 h. Upon completion, 2:1 Et_2O:EtOAc (30 mL) was added to the reaction and the reaction mixture was washed with H_2O (3×10 mL) and brine (10 mL). The combined organic layers were dried over $MgSO_4$ and solvent was removed by reduced pressure. The resulting crude was stirred in EtOAc (5mL) and HCl (0.1 M, 5 mL) over night. The aqueous layer was washed with EtOAC (10 mL) and the combined organic layers were then stirred in sat. K_2CO_3 (15 mL) for 30 minutes at 30 °C. The above acid followed by base wash was carried out to remove any co-eluting pinacol boronic ester and corresponding phenol. The aqueous layer was washed with EtOAC and the organic layer was dried over $MgSO_4$ and solvent was removed under reduced pressure. The resulting crude was purified by silica gel column chromatography (25:1 hexane:EtOAc) to yield 4-(4-chlorophenyl)-2-methyl-2-(naphthalen-2-ylmethyl)cyclopent-4-ene-1,3-dione **2bh** (28.8 mg, 0.080 mmol, 77%) as a yellow solid.

M.p.= 96-98 °C; R_f = 0.54 (5:1 petroleum ether:EtOAc); ν_{max}/cm^{-1} = 2027, 1740, 1694, 1595, 1559, 1508, 1488, 1452, 1405, 1373, 1328, 1294, 1231, 1110, 1091, 1049, 1014, 988, 862, 833, 780, 750, 733, 667, 620, 560; ^{1}H NMR (300 MHz, $CDCl_3$) δ 7.71 – 7.65 (m, 2H, Ar-H), 7.62 – 7.55 (m, 3H, Ar-H), 7.46 – 7.42 (s, 1H, Ar-H), 7.41 – 7.35 (m, 2H, Ar-H), 7.31 (d, *J* = 8.6 Hz, 2H, Ar-H), 7.07 (dd, *J* = 8.4, 1.8 Hz, 1H, Ar-H), 6.88 (s, 1H, =CH), 3.25 (d, J = 13.5 Hz, 1H, C<u>H</u>H), 3.21 (d, J = 13.5 Hz, 1H, CH<u>H</u>),1.39 (s, 3H, CH_3); ^{13}C NMR (75 MHz, $CDCl_3$) δ = 206.3 (C), 205.3 (C), 155.7 (C), 141.0 (CH), 137.7 (C), 133.3 (CH), 133.2 (C), 132.3 (C), 130.2 (CH), 129.1 (CH), 128.5 (CH), 128.0 (CH), 127.7 (CH), 127.7 (CH), 127.5 (CH), 127.2 (C), 126.1 (CH), 125.8 (CH), 54.1 (C), 41.7 (CH_2), 19.7 (CH_3); HRMS (APCI) *m/z calc.* for $C_{23}H_{17}ClO_2$: 361.0990 [M+H]$^+$; found: 361.0993.

4-(4-Chlorophenyl)-2-methyl-2-phenylcyclopent-4-ene-1,3-dione (2ch)

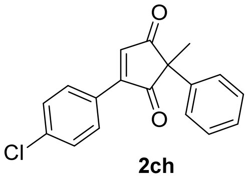

2ch

2-Methyl-2-phenylcyclopentane-1,3-dione **4c** (18.8 mg, 0.100 mmol, 1.0 equiv.), 1,10-phenanthroline (5.3 mg, 29.4 µmol, 0.3 equiv.), $Pd(OAc)_2$ (3.4 mg, 15.1 µmol, 0.15 equiv.) and 2-(4-chlorophenyl)-4,4,5,5- tetramethyl-1,3,2-dioxaborolane **9h** (71.5 mg, 0.300 mmol, 3.0 equiv.) were premixed and stirred in DMF (1 mL) for 20 minutes at room temperature before being heated at 120 °C in an O_2 atmosphere (balloon) for 72 h. Upon completion, 2:1 Et_2O:EtOAc (30 mL) was added to the reaction and the reaction mixture was washed with H_2O (3×10 mL) and brine (10 mL). The combined organic layers were dried over $MgSO_4$ and solvent was removed under reduced pressure. The resulting crude was purified by silica gel column chromatography (25:1 hexane:EtOAc) to yield 4-(4-chlorophenyl)-2-methyl-2-phenylcyclopent-4-ene-1,3-dione **2ch** (22.3 mg, 0.075 mmol, 75%) as a yellow solid.

M.p. 105-109 °C; R_f = 0.59 in 5:1 petroleum ether:EtOAc; ν_{max}/cm^{-1} = 3048, 2980, 1739, 1689,1588, 1484, 1443 1405, 1374, 1316, 1294, 1252,1202, 1184, 1091, 1056, 1038, 1013, 916, 894, 836, 800, 776, 763, 724, 712, 701, 585; ^1H NMR (300 MHz, $CDCl_3$) δ 7.94 (d, *J* = 8.8 Hz, 2H, Ar-H), 7.47 (d, *J* = 8.8 Hz, 2H, Ar-H), 7.42 (s, 1H, Alkene-H), 7.37 – 7.24 (m, 5H, Ar-H), 1.65 (s, 3H, CH_3); ^{13}C NMR (75 MHz, $CDCl_3$) δ = 203.9 (C), 203.2 (C), 154.9 (C), 140.4 (CH), 138.2 (C), 137.3 (C), 130.6 (CH), 129.4 (CH), 128.9 (CH), 127.8 (CH), 127.4 (C), 126.4 (CH), 56.1 (C), 19.9 (CH_3); HRMS (APCI) *m/z calc.* for $C_{18}H_{13}ClO_2$: 297.0677 $[M+H]^+$; found: 297.0679.

4-(4-Chlorophenyl)-2-(2-methoxyphenyl)-2-methylcyclopent-4-ene-1,3-dione (2dh)

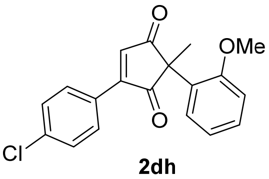

2dh

2-(2-Methoxyphenyl)-2-methylcyclopentane-1,3-dione **4d** (21.7 mg, 0.100 mmol, 1.0 equiv.), 1,10-phenanthroline (5.4 mg, 30.0 µmol, 0.3 equiv.), $Pd(OAc)_2$ (3.4 mg, 15.1 µmol, 0.15 equiv.) and 2-(4-chlorophenyl)-4,4,5,5- tetramethyl-1,3,2-dioxaborolane **9h** (71.6 mg, 0.300 mmol, 3.0 equiv.) were premixed and stirred in DMF (1 mL) for 20 minutes at room temperature before being heated at 120 °C in an O_2 atmosphere (balloon) for 72 h. Upon completion, 2:1 Et_2O:EtOAc (30 mL) was added to the reaction and the reaction mixture was washed with H_2O (3×10 mL) and brine (10 mL). The combined organic layers were dried over $MgSO_4$ and solvent was removed under reduced pressure. The resulting crude was purified by silica gel column chromatography (15:1 → 10:1 hexane:EtOAc) to yield 4-(4-chlorophenyl)-2-(2-methoxyphenyl)-2-methylcyclopent-4-ene-1,3-dione **2dh** (25.1 mg, 0.077 mmol, 77%) as a yellow solid.

M.p. 180-184; R_f= 0.55 in 5:1 petroleum ether:EtOAc; ν_{max}/cm^{-1} = 3058, 2995, 2975, 1740, 1692, 1590, 1559, 1492, 1461, 1435, 1405, 1372, 1294, 1282, 1259, 1246, 1200, 1180, 1128, 1090, 1063, 1042, 1025, 1014, 972, 922, 838, 813, 781, 752, 722, 702, 629, 594; ^{1}H NMR (300 MHz, $CDCl_3$) δ 7.94 (d, *J* = 8.8 Hz, 2H, Ar-H), 7.48 (d, *J* = 8.8 Hz, 2H, Ar-H), 7.42 (dd, *J* = 7.7, 1.6 Hz, 1H, Ar-H), 7.33 – 7.29 (m, 1H, Ar-H), 7.28 (s, 1H, Alkene-H), 7.05 (td, *J* = 7.6, 1.2 Hz, 1H, Ar-H), 6.77 (dd, *J* = 8.2, 1.1 Hz, 1H, Ar-H), 3.54 (s, 3H, OCH_3), 1.64 (s, 3H, CH_3); ^{13}C NMR (75 MHz, $CDCl_3$) δ = 205.4 (C), 205.2 (C), 156.1 (C), 152.3 (C), 137.6 (C), 137.2 (CH), 130.4 (CH), 129.6 (CH), 129.3 (CH), 129.1 (CH), 128.1 (C), 125.8 (C), 121.4 (CH), 110.8 (CH), 55.3 (CH_3), 54.7 (C), 19.2 (CH_3); HRMS (APCI) *m/z calc.* for $C_{19}H_{15}ClO_3$: 327.0792 $[M+H]^+$; found: 327.0786.

4-(4-Chlorophenyl)-2-methyl-2-(naphthalene-2-yl)cyclopent-4-ene-1,3-dione (2eh)[3]

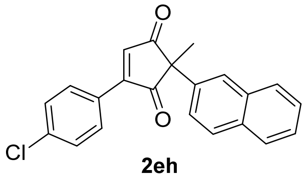

2eh

2-Methyl-2-(naphthalene-2-yl)cyclopentane-1,3-dione **4e** (23.8 mg, 0.099 mmol, 1.0 equiv.), 1,10-phenanthroline (5.4 mg, 30.0 μmol, 0.3 equiv.), $Pd(OAc)_2$ (3.4 mg, 15.1 μmol, 0.15 equiv.) and 2-(4-chlorophenyl)-4,4,5,5- tetramethyl-1,3,2-dioxaborolane **9h** (72.0mg, 0.302 mmol, 3.0 equiv.) were premixed and stirred in DMF (1 mL) for 20 minutes at room temperature before being heated at 120 °C in an O_2 atmosphere (balloon) for 71 h. Upon completion, 2:1 Et_2O:EtOAc (30 mL) was added to the reaction and the reaction mixture was washed with H_2O (3×10 mL) and brine (10 mL). The combined organic layers were dried over $MgSO_4$ and solvent was removed under reduced pressure. The resulting crude was purified by silica gel column chromatography (30:1 hexane:EtOAc) to yield 4-(4-chlorophenyl)-2-methyl-2-(naphthalene-2-yl)cyclopent-4-ene-1,3-dione **2eh** (24.4 mg, 0.0703 mmol, 71%) as a yellow solid.

Mp: 134-136 °C (hexane/EtOAc) (literature mp: 130-135 °C);[3] Rf: 0.41 in 5:1 petroleum ether:EtOAc; ^{1}H NMR (400 MHz, $CDCl_3$) δ 7.95 (d, *J* = 8.7 Hz, 2H, Ar-H), 7.86 – 7.76 (m, 4H, Ar-H), 7.52 – 7.45 (m, 6H, Ar-H), 1.75 (s, 3H, CH_3); ^{13}C NMR (75 MHz, $CDCl_3$) δ 203.9 (C), 203.3 (C), 155.0 (C), 140.5 (CH), 138.4 (C), 134.7 (C), 133.3 (C), 132.7 (C), 130.8 (CH), 129.5 (CH), 128.9 (CH), 128.3 (CH), 127.7 (CH), 127.5 (C), 126.6 (CH), 125.8 (CH), 124.1 (CH), 56.4 (C), 20.2 (CH_2), 1.2 (CH_3); HRMS (TOP MS ASAP+) *m/z calc.* for $C_{22}H_{16}ClO_2$: 347.0833 $[M+H]^+$; found: 347.0835.

4-(4-Chlorophenyl)-2-isobutyl-2-methylcyclopent-4-ene-1,3-dione (2fh)

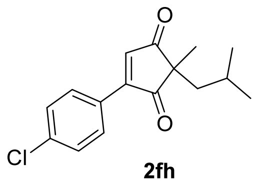

2fh

2-Isobutyl-2-methylcyclopentane-1,3-dione **4f** (16.8 mg, 0.100 mmol, 1.0 equiv.), 1,10-phenanthroline (5.4 mg, 30.0 μmol, 0.3 equiv.), $Pd(OAc)_2$ (3.4 mg, 15.1 μmol, 0.15 equiv.) and 2-(4-chlorophenyl)-4,4,5,5- tetramethyl-1,3,2-dioxaborolane **9h** (71.5 mg, 0.300 mmol, 3.0 equiv.) were premixed and stirred in DMF (1 mL) for 20 minutes at room temperature before being heated at 120 °C in an O_2 atmosphere (balloon) for 72 h. Upon completion, 2:1 Et_2O:EtOAc (30 mL) was added to the reaction and the reaction mixture was washed with H_2O (3×10 mL) and brine (10 mL). The combined organic layers were dried over $MgSO_4$ and solvent was removed under reduced pressure. The resulting crude was purified by silica gel column chromatography (20:1 hexane:EtOAc) to yield 4-(4-chlorophenyl)-2-isobutyl-2-methylcyclopent-4-ene-1,3-dione **2fh** (22.1 mg, 0.080 mmol, 80%) as a yellow oil.

R_f = 0.69 in 5:1 petroleum ether:EtOAc; ν_{max}/cm^{-1} = 2960, 2929, 2871, 1741, 1694, 1595, 1487,1453, 1428, 1388, 1293, 1276, 1246, 1137, 1092, 1-39, 1014, 895, 834, 793, 777, 756, 705, 567; ^{1}H NMR (300 MHz, $CDCl_3$) δ 7.91 (d, *J* = 8.7 Hz, 2H, Ar-H), 7.47 (d, *J* = 8.7 Hz, 2H, Ar-H), 7.33 (s, 1H, =CH), 1.74 (d, *J* = 1.8 Hz, 1H, C<u>H</u>H), 1.72 (d, *J* = 1.8 Hz, 1H, CH<u>H</u>), 1.49 - 1.34 (m, 1H, -C<u>H</u>(CH_3)$_2$), 1.21 (s, 3H, CH_3), 0.81 (d, J = 6.6 Hz, 3H, CH_3), 0.76 (d, *J* = 6.6 Hz, 3H, CH_3); ^{13}C NMR (75 MHz, $CDCl_3$) δ = 206.9 (C), 206.2 (C), 154.7 (C), 140.2 (CH), 138.0 (C), 130.5 (CH), 129.7 (CH), 127.6 (C), 51.9 (C), 44.2 (CH_2), 25.5 (CH), 24.0 (CH_3), 23.9 (CH_3), 21.7 (CH_3); HRMS (FTMS + p NSI) *m/z calc.* for $C_{16}H_{16}ClO_2$: 277.0990 [M+H]$^+$; found: 277.0993.

Methyl 2-(3-(4-chlorophenyl)-1-methyl-2,5-dioxocyclopent-3-en-1-yl) (2gh)

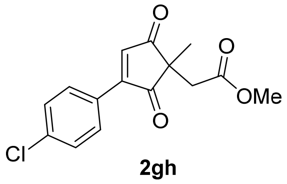

2gh

Methyl 2-(1-methyl-2,5-dioxocyclopentyl)acetate **4g** (18.4 mg, 0.100 mmol, 1.0 equiv.), 1,10-phenanthroline (5.3 mg, 29.4 μmol, 0.3 equiv.), $Pd(OAc)_2$ (3.3 mg, 14.7 μmol, 0.15 equiv.) and 2-(4-chlorophenyl)-4,4,5,5- tetramethyl-1,3,2-dioxaborolane **9h** (71.5 mg, 0.300 mmol, 3.0 equiv.) were premixed and stirred in DMF (1 mL) for 20 minutes at room temperature before being heated at 120 °C in an O_2 atmosphere (balloon) for 72 h. Upon completion, 2:1 Et_2O:EtOAc (30 mL) was added to the reaction and the reaction mixture was washed with H_2O (3×10 mL) and brine (10 mL). The combined organic layers were dried over $MgSO_4$ and solvent was removed under reduced pressure. The resulting crude was purified by silica gel column chromatography (6:1 hexane:EtOAc) to yield methyl-2-(3-(4-chlorophenyl)-1-methyl-2,5-dioxocyclopent-3-en-1-yl)acetate **2gh** (19.7 mg, 0.068 mmol, 67%) as a yellow oil.

M.p. 137-140 °C; R_f = 0.57 in 5:1 petroleum ether:EtOAc; ν_{max}/cm^{-1} = 3095, 3067, 2951, 1731, 1690, 1596, 1558, 1460, 1436, 1407, 1350, 1321, 1295, 1251,1209, 1118, 1091, 1056, 1017, 1009, 915, 897, 854, 843, 831, 716, 661, 595, 557; ^{1}H NMR (300 MHz, $CDCl_3$) δ 7.91 (d, J = 8.8 Hz, 2H, Ar-H), 7.45 (d, J = 8.8 Hz, 2H, Ar-H), 7.35 (s, 1H, =CH), 3.56 (s, 3H, CH_3), 2.93 (apparent s, 2H, CH_2), 1.24 (s, 3H, CH_3); ^{13}C NMR (75 MHz, $CDCl_3$) δ = 204.7 (C), 204.0 (C), 170.9 (C), 154.4 (C), 139.5 (CH), 137.8 (C), 130.5 (CH), 129.3 (CH), 127.8 (C), 52.1 (CH_3), 49.3 (C), 37.9 (CH_2), 20.9 (CH_3); HRMS (APCI) *m/z calc.* for $C_{15}H_{13}ClO_4$: 293.0575 [M+H]$^+$; found: 293.0578.

2-((Benzyloxy)methyl)-4-(4-chlorophenyl)-2-methylcyclopent-4-ene-1,3-dione (2hh)

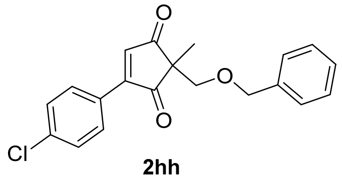

2hh

2-((Benzyloxy)methyl)-2-methylcyclopentane-1,3-dione **4h** (23.4 mg, 0.101 mmol, 1.0 equiv.), 1,10-phenanthroline (5.3 mg, 29.4 µmol, 0.3 equiv.), $Pd(OAc)_2$ (3.4 mg, 15.1 µmol, 0.15 equiv.) and 2-(4-chlorophenyl)-4,4,5,5- tetramethyl-1,3,2-dioxaborolane **9h** (71.4 mg, 0.299 mmol, 3.0 equiv.) were premixed and stirred in DMF (1 mL) for 20 minutes at room temperature before being heated at 120 °C in an O_2 atmosphere (balloon) for 71 h. Upon completion, 2:1 Et_2O:EtOAc (30 mL) was added to the reaction and the reaction mixture was washed with H_2O (3×10 mL) and brine (10 mL). The combined organic layers were dried over $MgSO_4$ and solvent was removed under reduced pressure. The resulting crude was purified by silica gel column chromatography (15:1 hexane:EtOAc). Solvent was removed under reduced pressure and co-eluted *p*-chlorophenol was removed by stirring product fractions over carbonate on polymer support beads (177 mg, 0.62 mmol) in DCM (0.5 mL) overnight. Removal of the polymer beads through filtration yielded 2-((benzyloxy)methyl)-4-(4-chlorophenyl)-2-methylcyclopent-4-ene-1,3-dione **2hh** (18.5 mg, 0.054 mmol, 54%) as a yellow oil.

R_f: 0.4 in 5:1 petroleum ether:EtOAc; ν_{max}/cm^{-1}: 2858 w, 1746 w, 1698 s, 1595 m, 1559 w, 1486 m, 1453 m, 1290 m, 1092 s, 836 m, 778 w, 697 m; ^1H NMR (400 MHz, $CDCl_3$) δ = 7.92 (d, *J* = 8.7 Hz, 2H, Ar-H), 7.47 (d, *J* = 8.7 Hz, 2H, Ar-H), 7.40 (s, 1H, =CH), 7.30 – 7.23 (m, 3H, Ar-H), 7.14 – 7.10 (m, 2H, Ar-H), 4.39 (s, 2H, CH_2Ph), 3.69 (d, J = 8.5 Hz, 1H, C<u>H</u>HOBn), 3.69 (d, J = 8.5 Hz, 1H, CH<u>H</u>OBn), 1.11 (s, 3H, CH_3); ^{13}C NMR (101 MHz, $CDCl_3$) δ = 205.3 (C), 204.4 (C), 156.0 (C), 141.6 (CH), 138.0 (C), 137.6 (C), 130.7 (CH), 129.4 (CH), 128.5 (CH), 127.9 (CH), 127.8 (2 × CH), 127.4 (CH), 73.6 (CH_2), 72.5 (CH_2), 53.0 (C), 15.4 (CH_3); HRMS (TOP MS ASAP+) *m/z calc.* for $C_{20}H_{18}ClO_3$: 341.0944 $[M+H]^+$; found: 341.0941.

3-(4-Chlorophenyl)-7'-methoxy-2',3'-dihydrospiro[cyclopentane-1,1'-inden]-3-ene-2,5-dione (2ih)

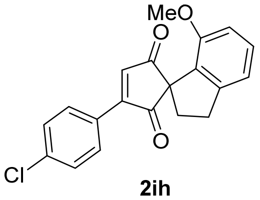

2ih

7'-Methoxy-2',3'-dihydrospiro[cyclopentane-1,1'-indene]-2,5-dione **4i** (23.0 mg, 0.099 mmol, 1.0 equiv.), 1,10-phenanthroline (5.3 mg, 30.1 μmol, 0.3 equiv.), $Pd(OAc)_2$ (3.4 mg, 15.1 μmol, 0.15 equiv.) and 2-(4-chlorophenyl)-4,4,5,5- tetramethyl-1,3,2-dioxaborolane **9h** (71.9 mg, 0.301 mmol, 3.0 equiv.) were premixed and stirred in DMF (1 mL) for 20 minutes at room temperature before being heated at 120 °C in an O_2 atmosphere (balloon) for 71 h. Upon completion, 2:1 Et_2O:EtOAc (30 mL) was added to the reaction and the reaction mixture was washed with H_2O (3×10 mL) and brine (10 mL). The combined organic layers were dried over $MgSO_4$ and solvent was removed under reduced pressure. The resulting crude was purified by silica gel column chromatography (8:1 hexane:EtOAc). Solvent was removed under reduced pressure and co-eluted *p*-chlorophenol was removed by stirring product fractions over carbonate on polymer support beads (47.2 mg, 0.165 mmol) in DCM (0.5 mL) for 1 h. Removal of the polymer beads through filtration yielded 3-(4-chlorophenyl)-7'-methoxy-2',3'-dihydrospiro[cyclopentane-1,1'-inden]-3-ene-2,5-dione **2ih** (22.9 mg, 0.068 mmol, 68%) as a yellow amorphous solid.

R_f: 0.4 in 5:1 petroleum ether:EtOAc; ν_{max}/cm^{-1}: 2933 w, 2850 w, 1741 w, 1695 s, 1560 m, 1479 m, 1091 m, 822 m, 733 m, 703 m; ^{1}H NMR (300 MHz, $CDCl_3$) δ = 7.93 (d, *J* = 8.8 Hz, 2H, Ar-H), 7.48 (d, *J* = 8.7 Hz, 2H, Ar-H), 7.37 (s, 1H, =CH), 7.22 (t, *J* = 7.8 Hz, 1H, Ar-H), 6.92 (dd, *J* = 7.5, 0.9 Hz, 1H, Ar-H), 6.60 (d, *J* = 8.0 Hz, 1H, Ar-H), 3.57 (s, 3H, OCH_3), 3.34 – 3.11 (m, 2H, CH_2), 2.50 – 2.32 (m, 2H, CH_2); ^{13}C NMR (75 MHz, $CDCl_3$) δ = 205.0 (C), 204.4 (C), 155.3 (C), 155.1 (C), 148.3 (C), 140.1 (CH), 137.8 (C), 130.6 (CH), 130.5 (CH), 129.4 (CH), 128.2 (C), 128.1 (C), 117.6 (CH), 108.6 (CH), 62.9 (C), 55.5 (CH_3), 34.5 (CH_2), 32.42 (CH_2); HRMS (FTMS p NSI) *m/z calc.* for $C_{20}H_{16}ClO_3$: 339.0782 $[M+H]^+$; found: 339.0786.

Enantioselective Desymmetrisation Reactions

4-(4-Methoxyphenyl)-2-methyl-2-(naphthalen-2-yl)cyclopent-4-ene-1,3-dione[3] (2ea)

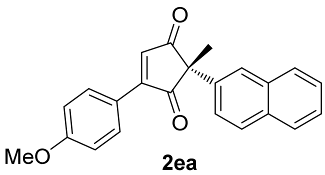

2ea

One-pot dehydrogenation/oxidative Heck desymmetrisation procedure

2-Methyl-2-(naphthalene-2-yl)cyclopentane-1,3-dione **4e** (23.8 mg, 0.099 mmol, 1.0 equiv.), (S)-4-(tert-butyl)-2-(5-(trifluoromethyl)pyridin-2-yl)-4,5-dihydrooxazole **13** (5.4 mg, 20.0 µmol, 0.2 equiv.), and $Pd(OAc)_2$ (2.2 mg, 9.8 µmol, 0.10 equiv.) were premixed and stirred in DMA (0.5 mL) for 20 minutes at room temperature before being heated at 120 °C in an O_2 atmosphere (balloon) for 72 h. $Pd(OAc)_2$ (2.2 mg, 9.8 µmol, 0.10 equiv.) and (S)-^{t}BuPyOx **13** (3.0mg, 11.4 µmol, 0.11 equiv.) were pre-mixed in DMA (0.1 mL) for 1 h. Para-methoxyphenyl boronic acid was dehydrated under vacuum with a heat gun to the corresponding boroxine **3a**, and **3a** was then added to the reaction (47.0 mg, 0.309 mmol, 3 equiv.) along with the catalyst mixture and DMA (0.4 mL). The reaction mixture was stirred at 50 °C under an atmosphere of O_2 (balloon) for 94 h. Upon completion, 2:1 Et_2O:EtOAc (30 mL) was added to the reaction and the reaction mixture was washed with H_2O (3×10 mL) and brine (10 mL). The combined organic layers were dried over $MgSO_4$ and solvent was removed under reduced pressure. The resulting crude was purified by silica gel column chromatography (10:1 hexane:EtOAc) was removed by reduced pressure and co-eluted *p*-MeO-phenol was removed by stirring product fractions over carbonate on polymer support beads (177 mg, 0.62 mmol) in DCM (0.5 mL) overnight. Removal of the polymer beads through filtration yielded 4-(4-methoxyphenyl)-2-methyl-2-(naphthalen-2-yl)cyclopent-4-ene-1,3-dione **2ea** (20.4 mg, 0.06 mmol, 60%) as a yellow powder.

M.p. 139 – 142 °C (hexane/EtOAc) (literature: 144 – 146 °C)[3]; R_f: 0.25 in 5:1 petroleum ether:EtOAc; νmax/cm^{-1}: 2919 w, 2833 w, 1733 w, 1683 s, 1600 m, 1579 m, 1506 m, 1239 s, 1099 m, 824 m,800 m, 775 m, 748 m; ^{1}H NMR (300 MHz, CDCl3) δ = 8.04 (d, *J* = 9.0 Hz, 2H), 7.84 – 7.76 (m, 4H), 7.50 (dd, *J* = 8.8, 2.0 Hz, 1H), 7.48 – 7.42 (m, 2H), 7.39 (s, 1H), 7.00 (d, *J* = 9.0 Hz, 2H), 3.88 (s, 3H), 1.74 (s, 3H); ^{13}C NMR (75 MHz, $CDCl_3$) δ = 204.7 (C), 203.5 (C), 162.8 (C), 155.6 (C), 138.1 (CH), 135.2 (C), 133.4 (C), 132.7 (C), 131.5 (CH), 128.8 (CH), 128.3 (CH), 127.6 (CH), 126.5 (CH), 126.4 (CH), 125.8 (CH), 124.3 (CH), 121.7 (C), 114.7 (CH), 56.5 (C), 55.7 (CH_3), 20.14 (CH_3).

$[\alpha]_D^{21}$ = +47.1 (*c* 0.34, $CHCl_3$); 74:26 e.r.; HPLC (CHIRALPAK IA, hexane:2-propanol: 95:5, flow rate: 1.0 mL min^{-1}, detection UV 254 nm, 25 °C) t_R of major isomer: 41.8 min, t_R of minor isomer: 71.7 min.

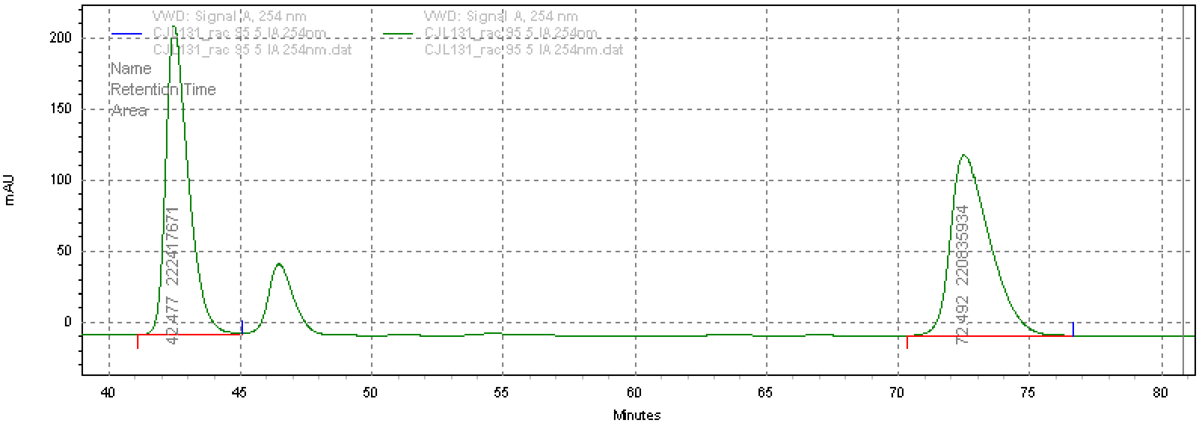

VWD: Signal A,
254 nm Results

Retention Time	Area	Area %	Height	Height %
42.477	222417671	50.18	3639814	63.10
72.492	220835934	49.82	2128496	36.90
Totals	443253605	100.00	5768310	100.00

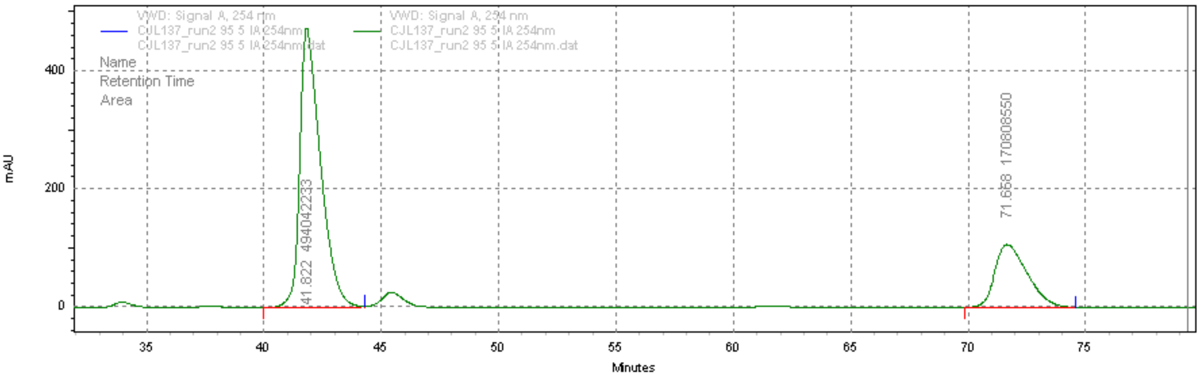

VWD: Signal A,
254 nm Results

Retention Time	Area	Area %	Height	Height %
41.822	494042233	74.31	7937449	81.63
71.658	170808550	25.69	1786744	18.37
Totals	664850783	100.00	9724193	100.00

Telescoped dehydrogenation/oxidative Heck desymmetrisation procedure

2-Methyl-2-(naphthalene-2-yl)cyclopentane-1,3-dione **4e** (23.8 mg, 0.099 mmol, 1.0 equiv.), (S)-4-(tert-butyl)-2-(5-(trifluoromethyl)pyridin-2-yl)-4,5-dihydrooxazole **13** (5.4 mg, 20.0 µmol, 0.2 equiv.), and $Pd(OAc)_2$ (2.2 mg, 9.8 µmol, 0.10 equiv.) were premixed and stirred in DMA (0.5 mL) for 20 minutes at room temperature before being heated at 120 °C in an O_2 atmosphere (balloon) for 72 h. The reaction mixture was filtered through a short silica plug with 2:1 hexane:EtOAc and the solvent was removed by reduced pressure. $Pd(OAc)_2$ (2.2 mg, 9.8 µmol, 0.10 equiv.) and (S)-^{t}BuPyOx **13** (3.0mg, 11.4 µmol, 0.11 equiv.) were pre-mixed in DMA (0.2 mL) for 1 h. Para-methoxyphenyl boronic acid was dehydrated under vacuum with a heat gun to the corresponding boroxine **3a**, and this was added to the reaction (47.0 mg, 0.309 mmol, 3 equiv.) along with the catalyst mixture and DMA (0.8 mL). The reaction mixture was stirred at 50 °C under an atmosphere of O_2 (balloon) for 94 h. Upon completion, 2:1 Et_2O:EtOAc (30 mL) was added to the reaction and the reaction mixture was washed with H_2O (3×10 mL) and brine (10 mL). The combined organic layers were dried over $MgSO_4$ and solvent was removed by reduced pressure. The resulting crude was purified by silica gel column chromatography (10:1 hexane:EtOAC) was removed under reduced pressure and co-eluted *p*-MeO-phenol was removed by stirring product fractions over carbonate on polymer support beads (177 mg, 0.62 mmol) in DCM (0.5 mL) overnight. Removal of the polymer beads through filtration yielded 4-(4-methoxyphenyl)-2-methyl-2-(naphthalen-2-yl)cyclopent-4-ene-1,3-dione **2ea** (23.7 mg, 0.07 mmol, 70%, 88:12 e.r.) as a yellow powder.

See one-pot procedure for characterisation.

$[\alpha]_D^{21}$ = +100.0 (*c* 0.68, $CHCl_3$); 88:12 e.r.; HPLC (CHIRALPAK IA, hexane:2-propanol: 95:5, flow rate: 1.0 mL min^{-1}, detection UV 254 nm, 25 °C) t_R of major isomer: 41.9 min, t_R of minor isomer: 72.3 min.

The (*S*)-stereochemistry was assigned by analogy based on the absolute stereochemistry determination by comparison with a known structure (preussidone), as described in ref. 2.[2]

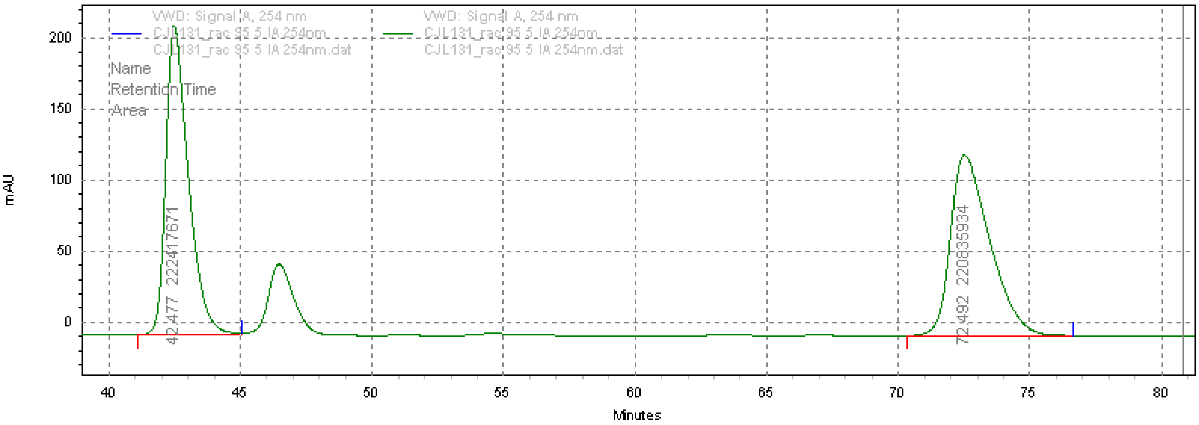

VWD: Signal A, 254 nm Results

Retention Time	Area	Area %	Height	Height %
42.477	222417671	50.18	3639814	63.10
72.492	220835934	49.82	2128496	36.90
Totals	443253605	100.00	5768310	100.00

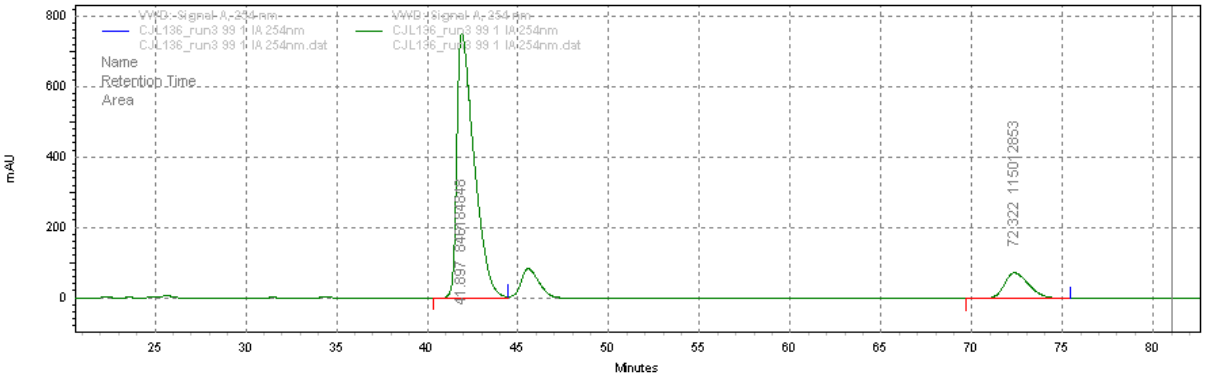

VWD: Signal A, 254 nm Results

Retention Time	Area	Area %	Height	Height %
41.897	846184848	88.03	12616201	91.14
72.322	115012853	11.97	1226107	8.86
Totals	961197701	100.00	13842308	100.00

Continuous Flow

2-Benzyl-4-(4-chlorophenyl)-2-methylcyclopent-4-ene-1,3-dione (2ah)

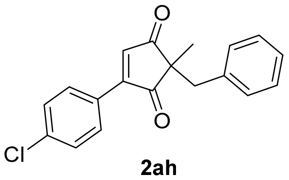

2ah

0.3 Mmol procedure:

A commercial flow reactor (easy-Photochem; Vapourtec Ltd.) was used for the continuous flow reactions, this was used to control all parameters. 2-Benzyl-2-methylcyclopentane-1,3-dione **4a** (60.7 mg, 0.3 mmol, 1.0 equiv.), 1,10-phenanthroline **10** (16.2 mg, 0.030 mmol, 0.3 equiv.), $Pd(OAc)_2$ (10.1 mg, 0.015 mmol, 0.15 equiv.) and 2-(4-chlorophenyl)-4,4,5,5- tetramethyl-1,3,2-dioxaborolane **9h** (214.4 mg, 0 mmol, 3.0 equiv.) were stirred in DMF (2 mL) for 20 minutes at room temperature in a round bottom flask. The homogeneous reaction mixture was pumped at a rate of 0.4 mL min^{-1} and O_2 was pumped at a rate of 0.4 mL min^{-1}. The two phases met at a T-juntion to achieve a segmented flow, which passed through a thermal reactor (2 mL volume) at a temperature of 120 °C. The reaction was cycled and to these continuous flow conditions for 72 h. Upon completion, 2:1 Et_2O:EtOAc (30 mL) was added to the reaction and the reaction mixture was washed with H_2O (2×10 mL) and brine (10 mL). The combined organic layers were dried over $MgSO_4$ and solvent was removed under reduced pressure. The resulting crude was purified by silica gel column chromatography (30:1 → 20:1 petrol:EtOAc) yielded 2-benzyl-4-(4-chlorophenyl)- 2-methylcyclopent-4-ene-1,3-dione **2ah** (48.8 mg, 0.157 mmol, 52%) as a yellow oil.

1.0 Mmol procedure:

A commercial flow reactor (easy-Photochem; Vapourtec Ltd.) was used for the continuous flow reactions, this was used to control all parameters. 2-Benzyl-2-methylcyclopentane-1,3-dione **4a** (202.2 mg, 1.00 mmol, 1.0 equiv.), 1,10-phenanthroline **10** (54.1 mg, 0.300 mmol, 0.3 equiv.), $Pd(OAc)_2$ (33.8 mg, 0.151 mmol, 0.15 equiv.) and 2-(4-chlorophenyl)-4,4,5,5- tetramethyl-1,3,2-dioxaborolane **9h** (718.1 mg, 3.01 mmol, 3.0 equiv.) were stirred in DMF (7.5 mL) for 20 minutes at room temperature in a round bottom flask. The homogeneous reaction mixture was pumped at a rate of 0.4 mL min^{-1} and O_2 was pumped at a rate of 0.4 mL min^{-1}. The two phases met at a T-juntion to achieve a segmented flow, which passed through a thermal reactor (10 mL volume) at a temperature of 120 °C. The reaction was cycled and to these continuous flow conditions for 72 h. Upon completion, 2:1 Et_2O:EtOAc (30 mL) was added to the reaction and the reaction mixture was washed with H_2O (2×10 mL) and brine (10 mL). The combined organic layers were dried over $MgSO_4$ and solvent was removed under reduced

pressure. The resulting crude was purified by silica gel column chromatography (30:1 → 20:1 petrol:EtOAc) yielded 2-benzyl-4-(4-chlorophenyl)- 2-methylcyclopent-4-ene-1,3-dione **2ah** (170.9 mg, 0.55 mmol, 55%) as a yellow oil.

R_f: 0.48 in 5:1 petroleum ether/EtOAc; ν_{max}/cm^{-1}:2925 w, 1741 w, 1694 v str, 1595 m, 1559 w, 1489 m, 834 m, 756 m, 701 m; ^1H NMR (300 MHz, $CDCl_3$) δ 7.67 – 7.60 (d, J = 8.6 Hz, 2H, Ar-H), 7.41 – 7.34 (d, J = 8.6 Hz, 2H, Ar-H), 7.13 – 7.06 (m, 3H, Ar-H), 7.02 (s, 1H, =CH), 6.98 – 6.92 (m, 3H, Ar-H), 3.06 (apparent s, 2H, CH_2Ph), 1.33 (s, 2H, CH_3); ^{13}C NMR (75 MHz, $CDCl_3$) δ 206.3 (C), 205.5 (C), 155.8 (C), 141.3 (CH), 137.9 (C), 135.8 (C), 130.4 (CH), 129.7 (CH), 129.3 (CH), 128.5 (CH), 127.4 (C), 127.2 (CH), 54.1 (C), 41.8 (CH_2), 19.6 (CH_3); HRMS (TOF MS ASAP+) *m/z* calc. for $C_{19}H_{15}ClO_2$: 3110839 $[M+H]^+$ found: 311.0842.

^{1}H and [13]C Spectra

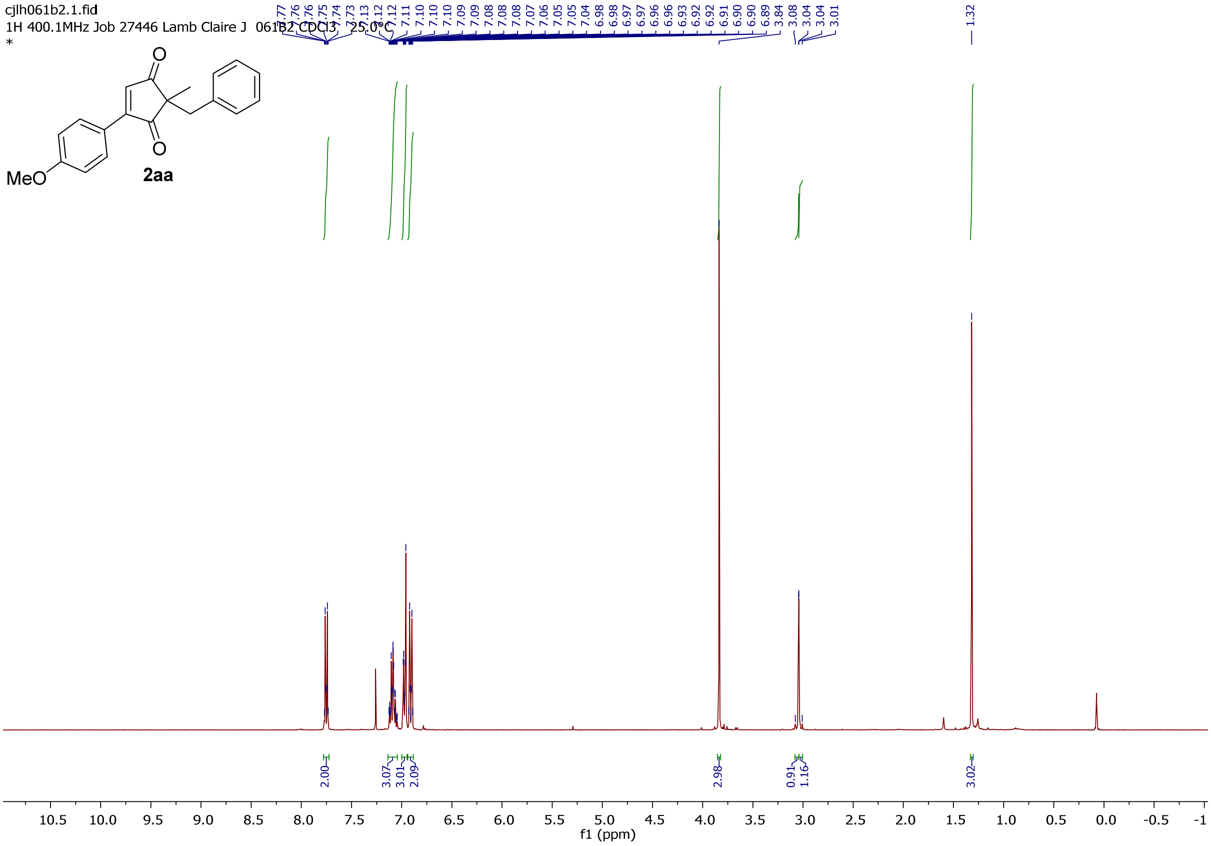

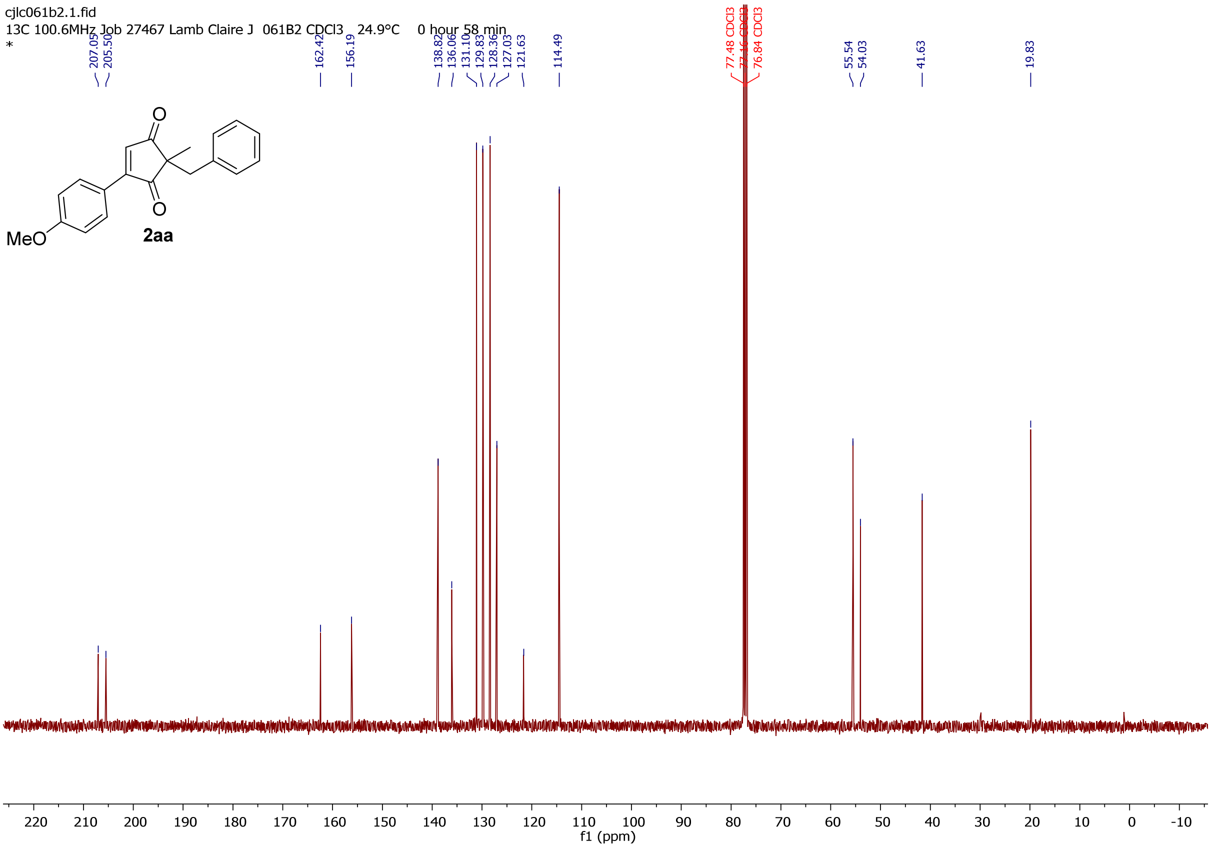

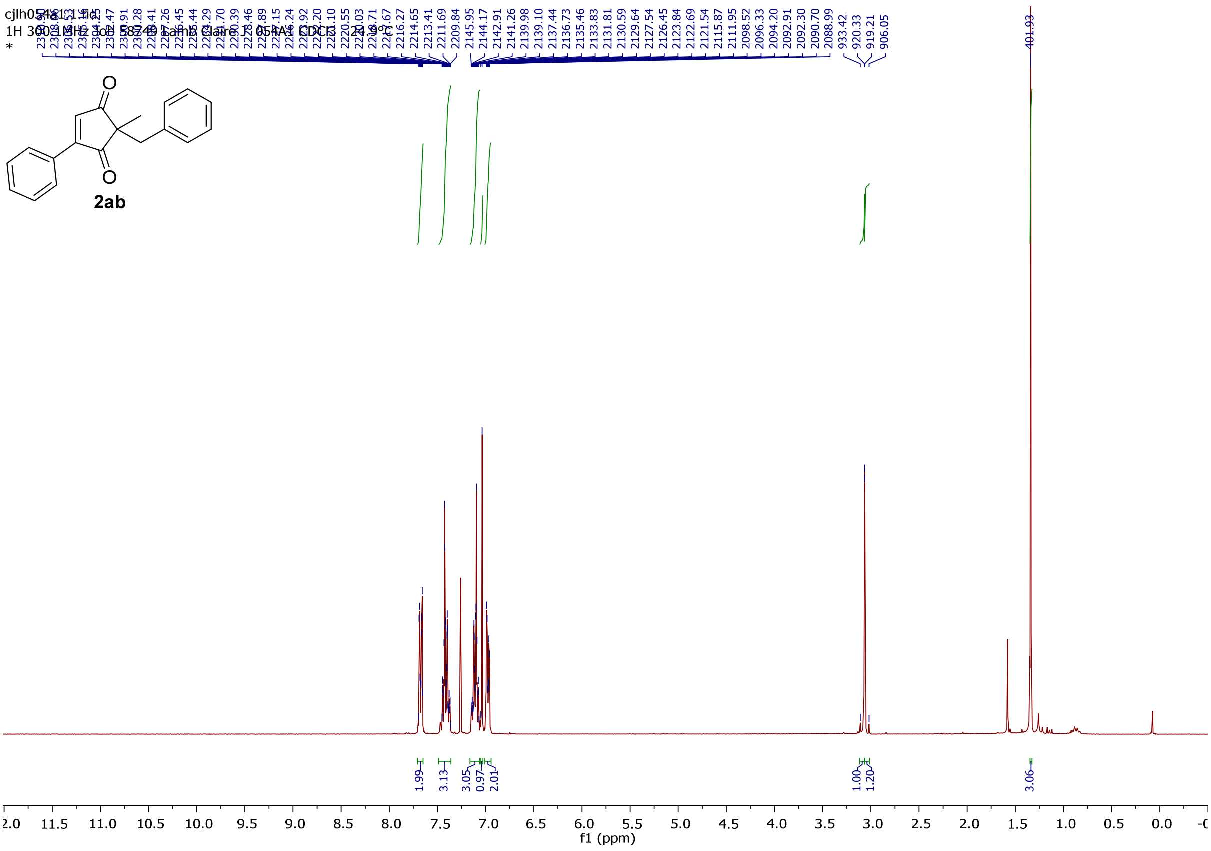
cjlh054a1.1.fid
2ab
933.42
920.33
919.21
906.05
401.93
1.99
3.13
3.05
0.97
2.01
1.00
1.20
3.06
f1 (ppm)

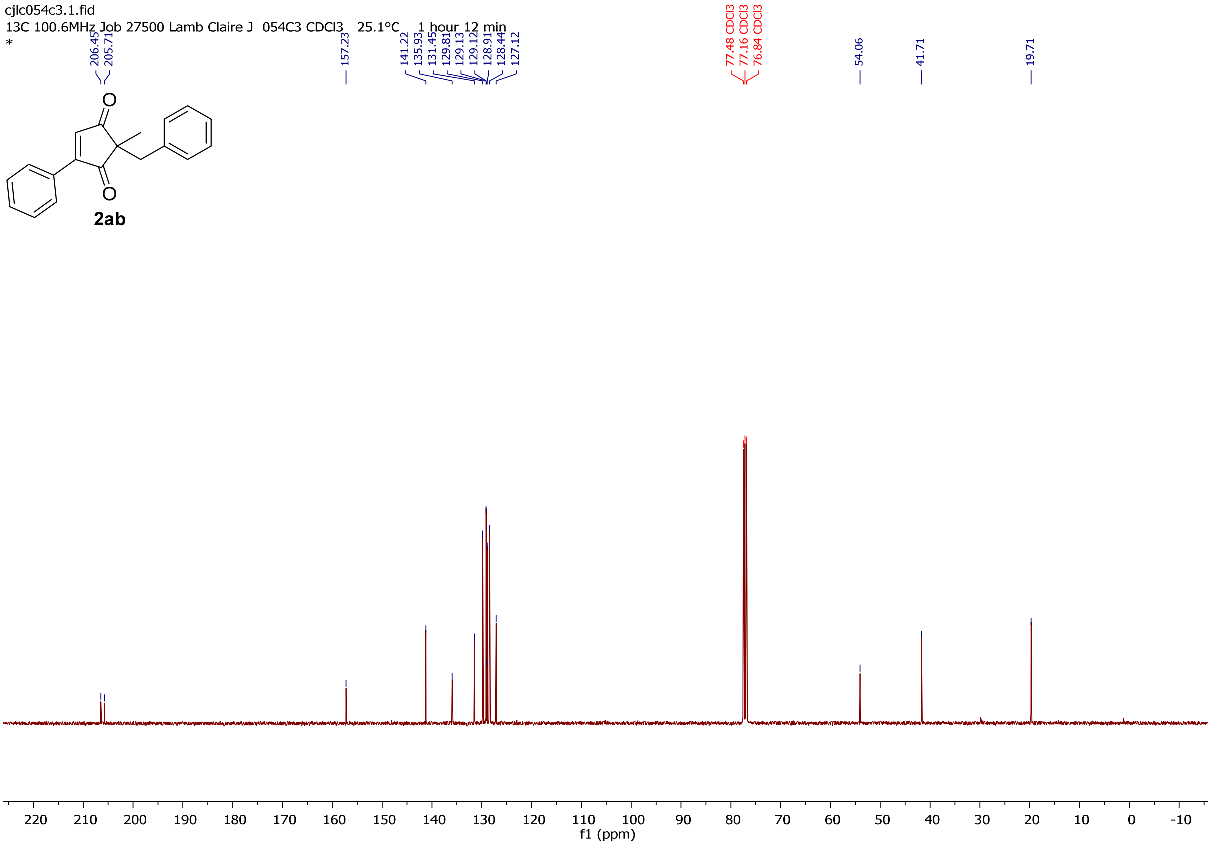
cjlc054c3.1.fid
13C 100.6MHz Job 27500 Lamb Claire J 054C3 CDCl3 25.1°C 1 hour 12 min
206.45
205.71
157.23
141.22
135.93
131.45
129.81
129.13
129.12
128.91
128.44
127.12
77.48 CDCl3
77.16 CDCl3
76.84 CDCl3
54.06
41.71
19.71
2ab
f1 (ppm)

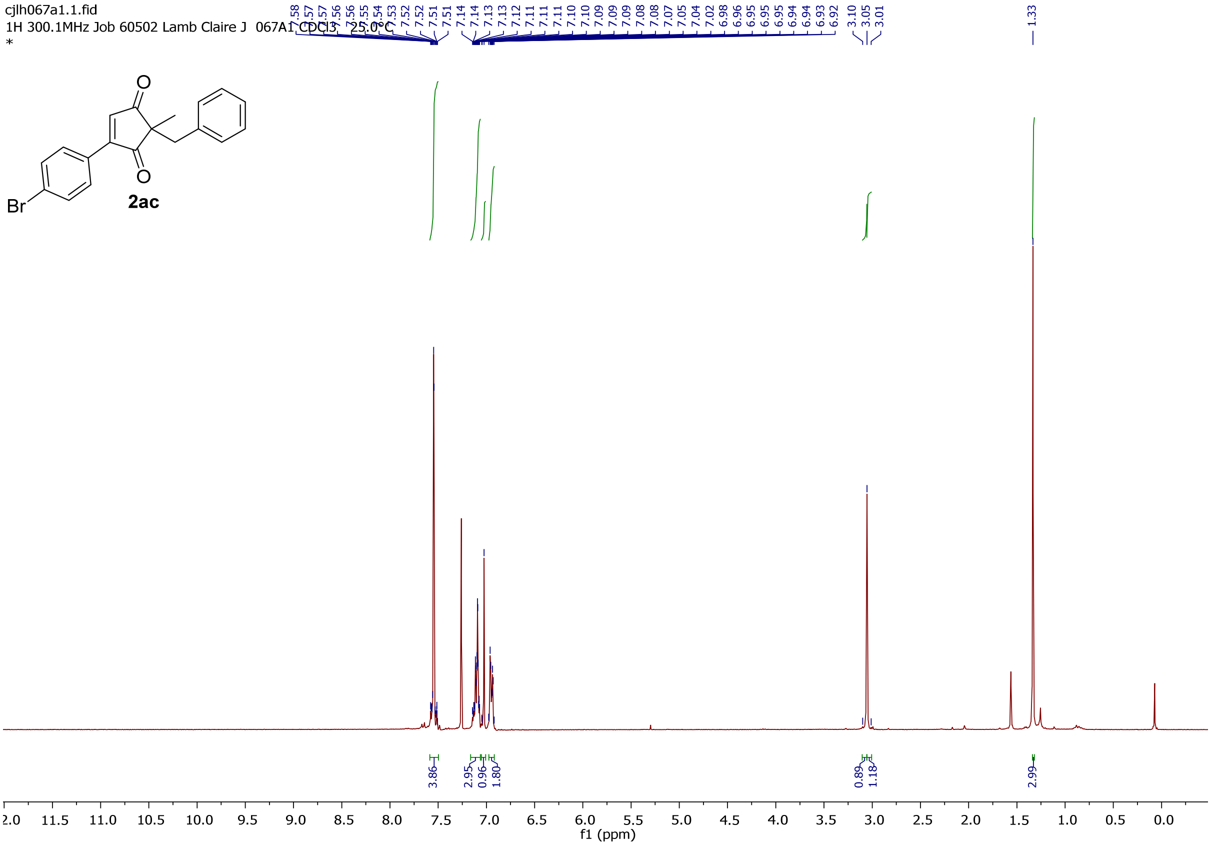
cjlh067a1.1.fid
1H 300.1MHz Job 60502 Lamb Claire J 067A1 CDCl3 25.0°C
*
Br
O
O
2ac
7.58 7.57 7.57 7.56 7.56 7.55 7.54 7.53 7.52 7.52 7.51 7.51 7.14 7.14 7.13 7.13 7.12 7.11 7.11 7.11 7.10 7.10 7.09 7.09 7.09 7.08 7.08 7.07 7.05 7.04 7.02 6.98 6.96 6.95 6.95 6.95 6.94 6.94 6.93 6.92
3.10 3.05 3.01
1.33
3.86 2.95 0.96 1.80 0.89 1.18 2.99
f1 (ppm)

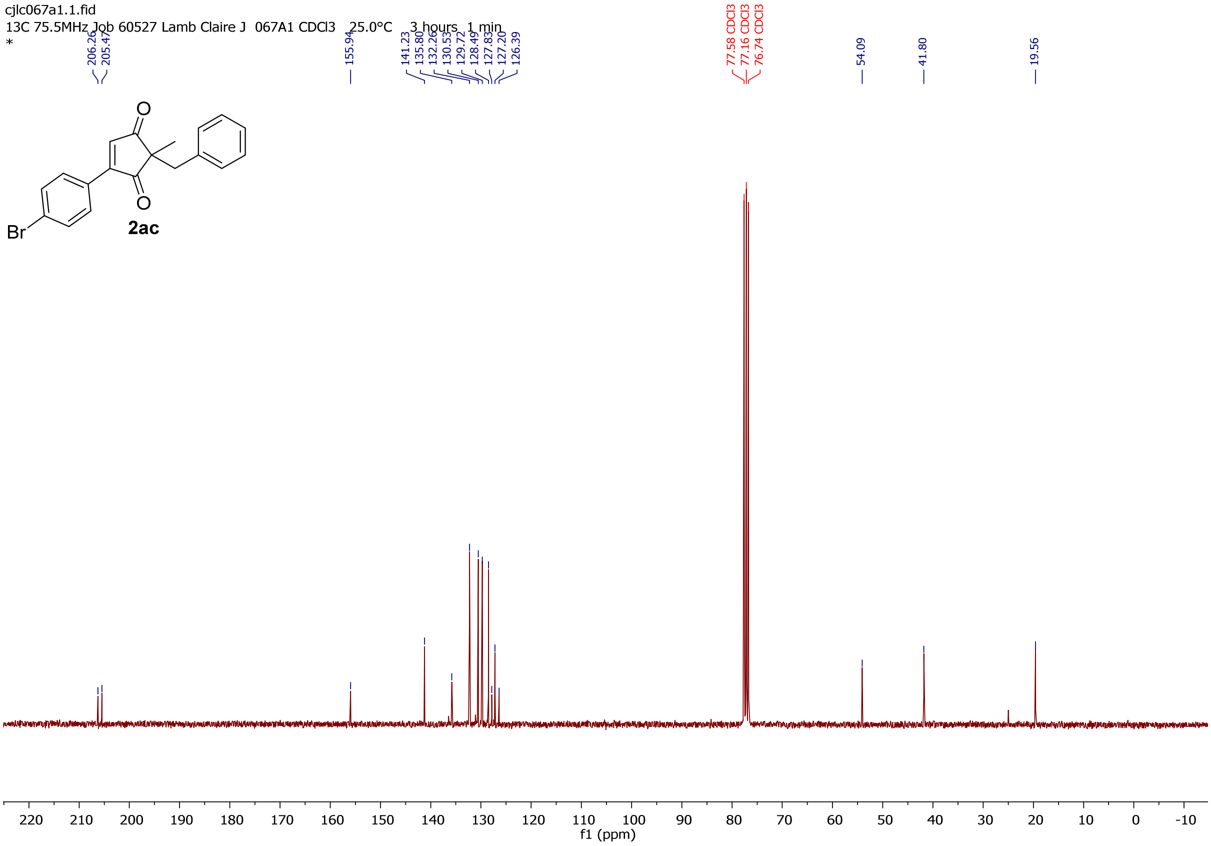
cjlc067a1.1.fid
13C 75.5MHz Job 60527 Lamb Claire J 067A1 CDCl3 25.0°C 3 hours 1 min
*
Br
O
O
2ac
206.26 205.47
155.94
141.23 135.80 132.26 130.53 129.72 128.49 127.83 127.20 126.39
77.58 CDCl3 77.16 CDCl3 76.74 CDCl3
54.09
41.80
19.56
f1 (ppm)

cjlh073a2.1.fid
1H 300.1MHz Job 62836 Lamb Claire J 073A2 CDCl3 24.9°C
*

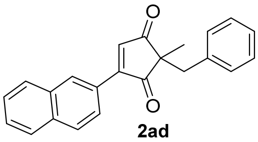

2ad

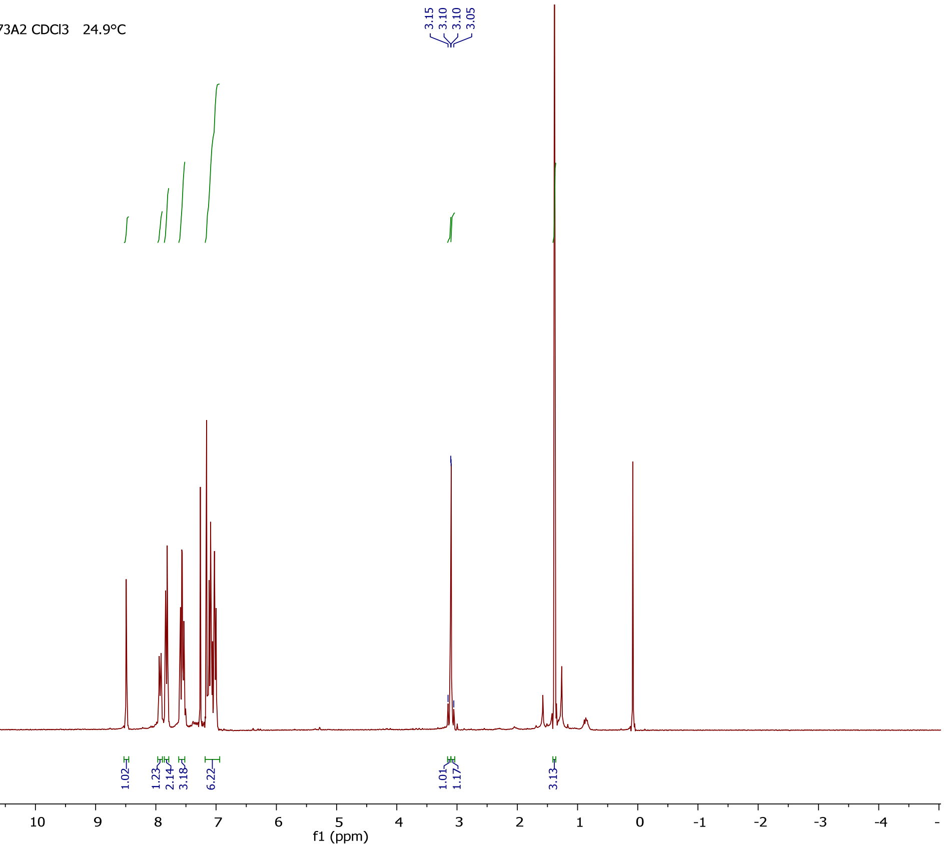

cjlc073a2.1.fid
13C 75.5MHz Job 62844 Lamb Claire J 073A2 CDCl3 25.0°C 3 hours 1 min
*

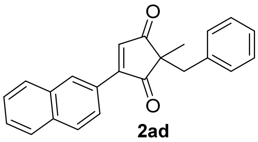

2ad

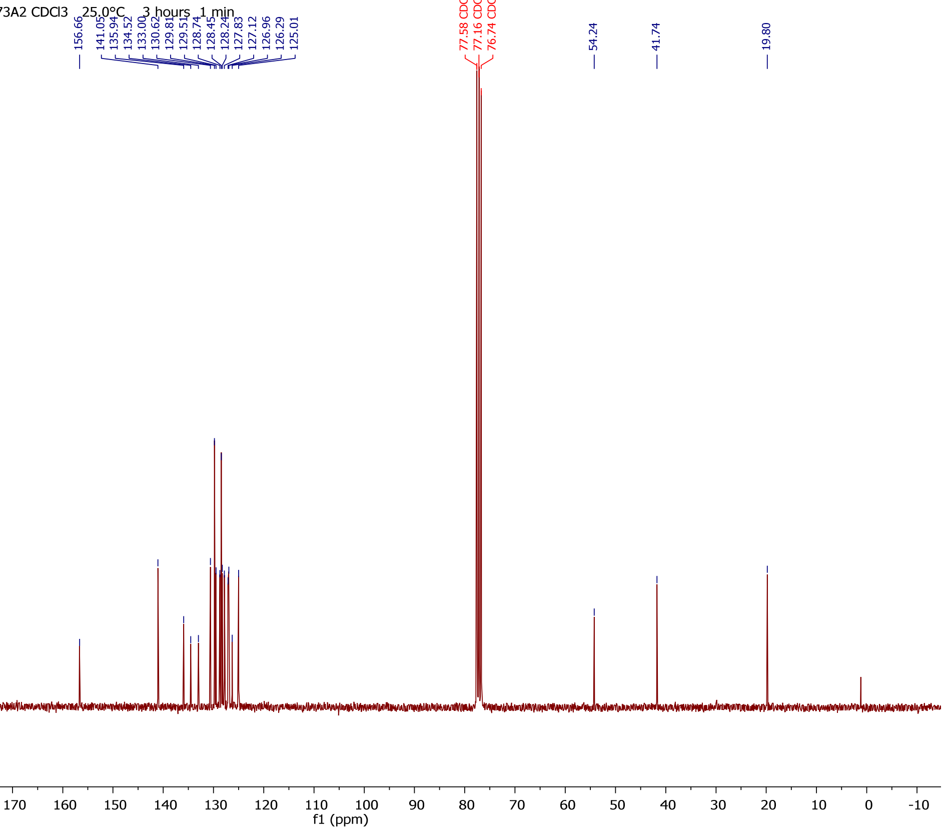

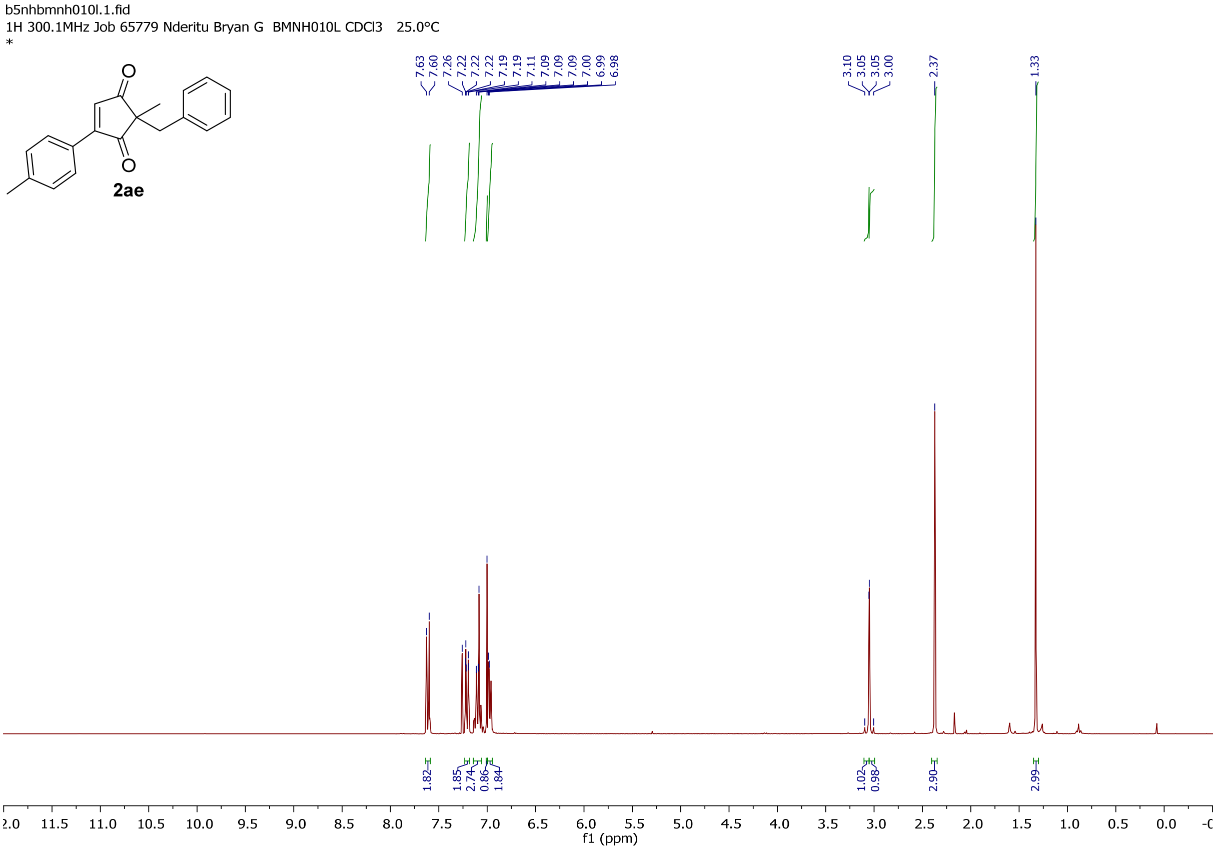
b5nhbmnh010l.1.fid
1H 300.1MHz Job 65779 Nderitu Bryan G BMNH010L CDCl3 25.0°C
*
2ae
7.63 7.60 7.26 7.22 7.22 7.22 7.19 7.19 7.11 7.09 7.09 7.09 7.00 6.99 6.98
3.10 3.05 3.05 3.00
2.37
1.33
1.82 1.85 2.74 0.86 1.84 1.02 0.98 2.90 2.99
f1 (ppm)

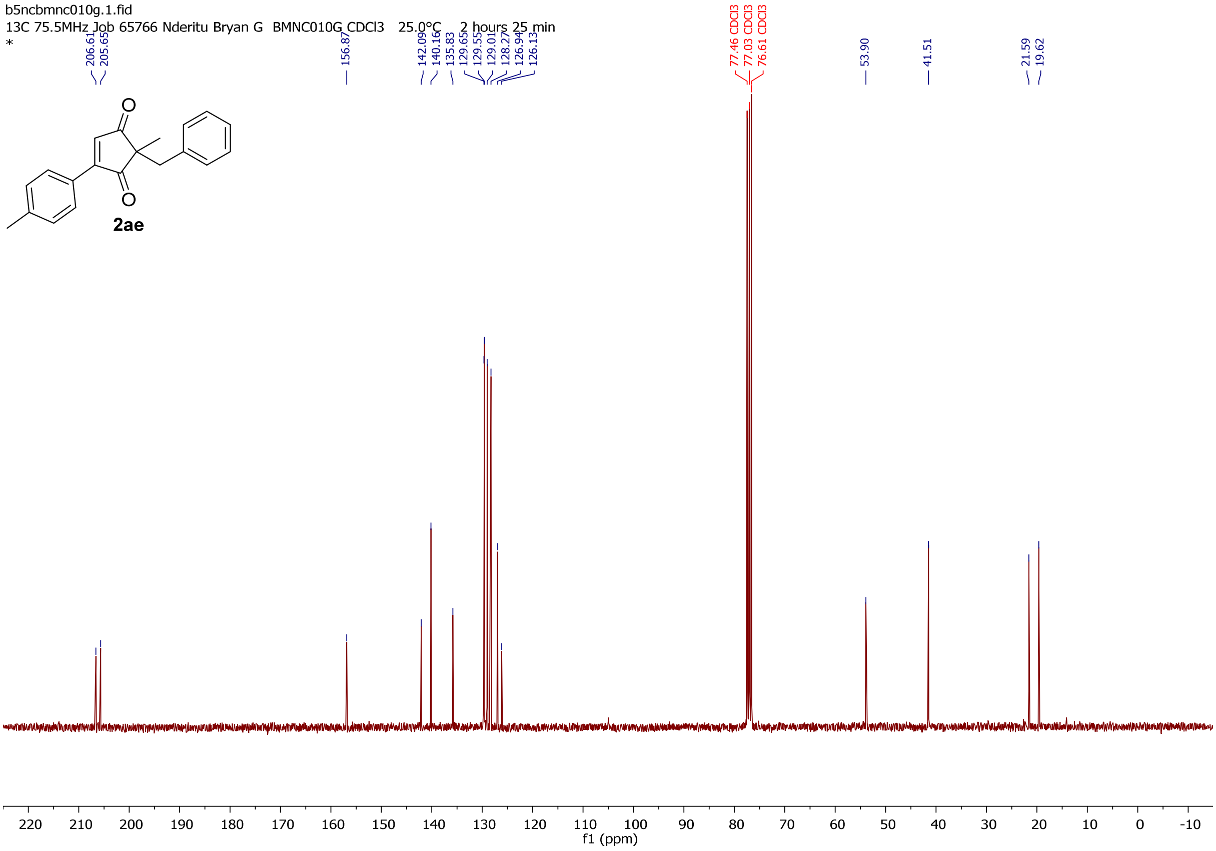
b5ncbmnc010g.1.fid
13C 75.5MHz Job 65766 Nderitu Bryan G BMNC010G CDCl3 25.0°C 2 hours 25 min
*
2ae
206.61 205.65
156.87
142.09 140.16 135.83 129.65 129.55 129.01 128.27 126.94 126.13
77.46 CDCl3 77.03 CDCl3 76.61 CDCl3
53.90
41.51
21.59 19.62
f1 (ppm)

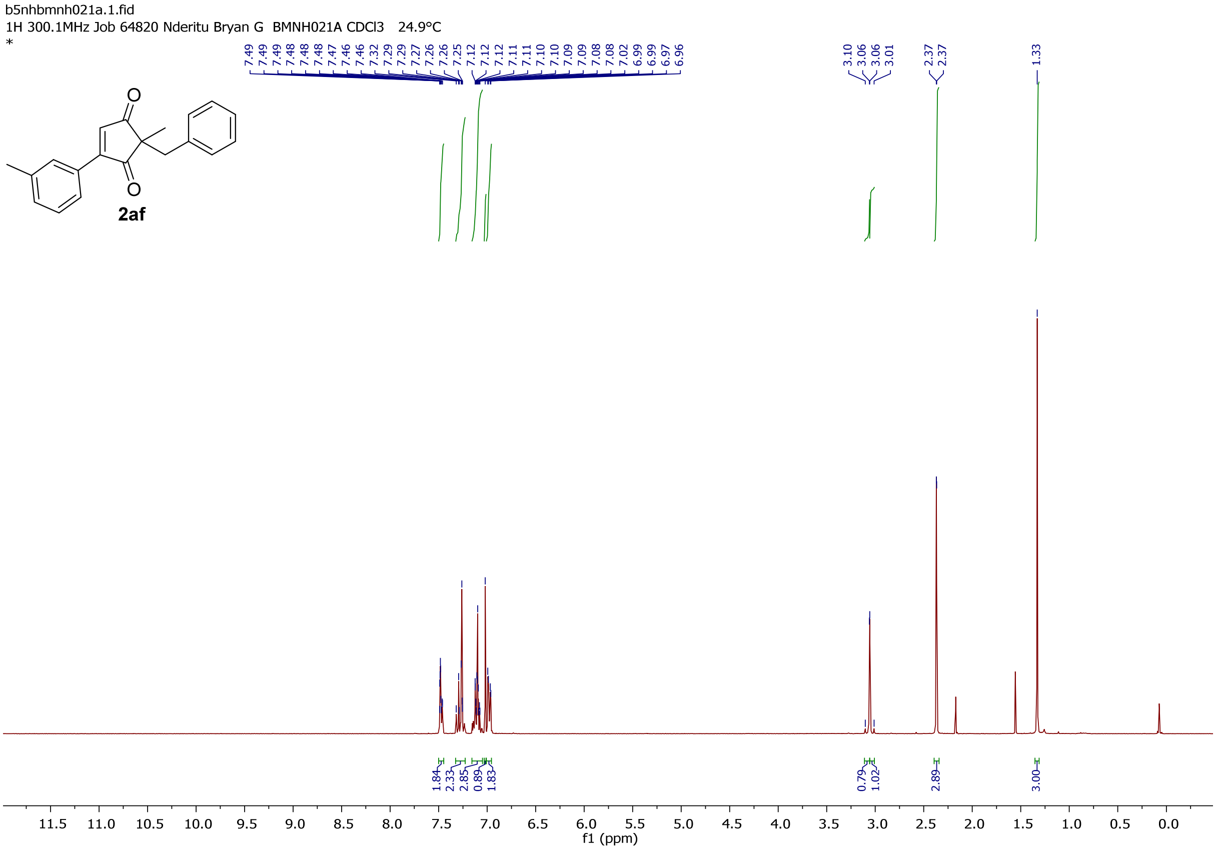

b5nhbmnh021a.1.fid
1H 300.1MHz Job 64820 Nderitu Bryan G BMNH021A CDCl3 24.9°C
*
7.49 7.49 7.49 7.48 7.48 7.48 7.47 7.46 7.46 7.32 7.29 7.29 7.27 7.26 7.26 7.25 7.12 7.12 7.12 7.11 7.11 7.10 7.10 7.09 7.09 7.08 7.08 7.02 6.99 6.99 6.97 6.96
3.10 3.06 3.06 3.01
2.37 2.37
1.33
2af
1.84 2.33 2.85 0.89 1.83
0.79 1.02
2.89
3.00
f1 (ppm)

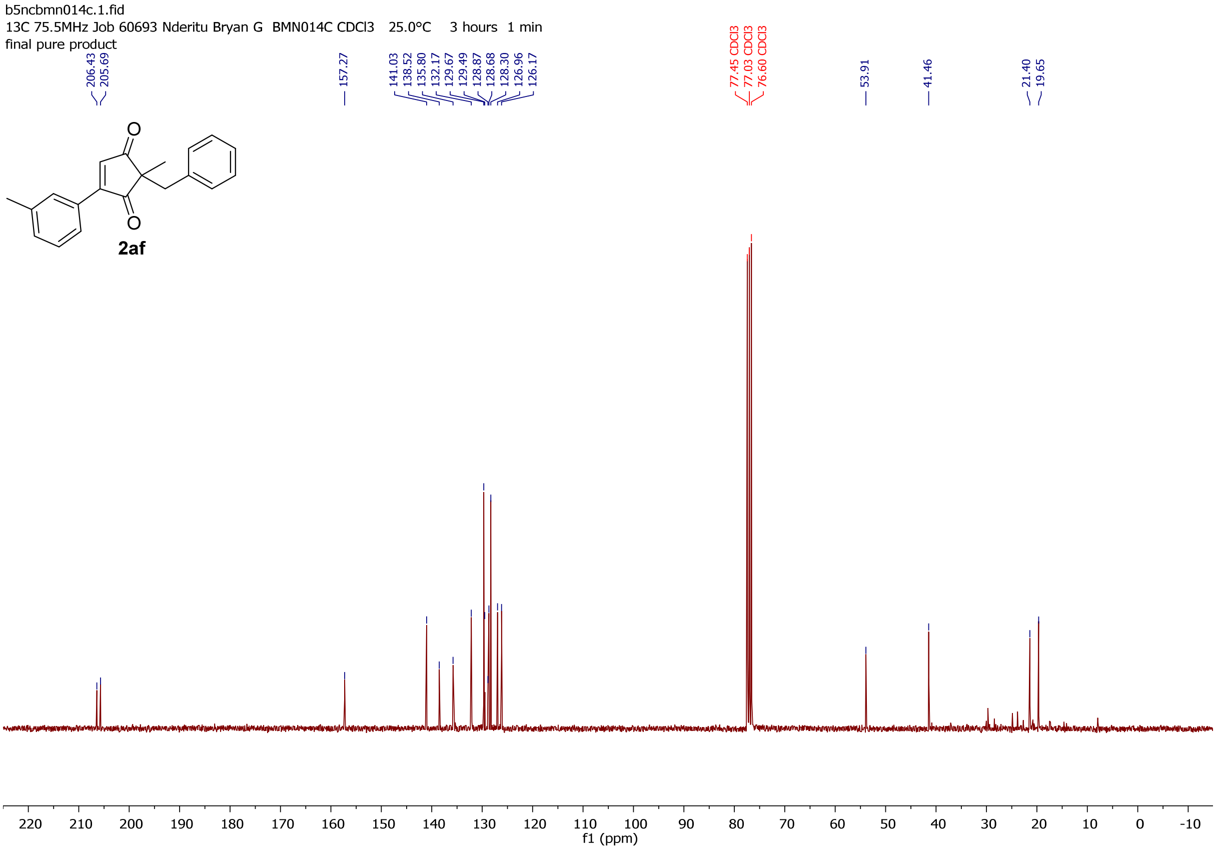

b5ncbmn014c.1.fid
13C 75.5MHz Job 60693 Nderitu Bryan G BMN014C CDCl3 25.0°C 3 hours 1 min
final pure product
206.43 205.69
157.27
141.03 138.52 135.80 132.17 129.67 129.49 128.87 128.68 128.30 126.96 126.17
77.45 CDCl3 77.03 CDCl3 76.60 CDCl3
53.91
41.46
21.40 19.65
2af
f1 (ppm)

b5nhbmn016.1.fid
1H 300.1MHz Job 60902 Nderitu Bryan G BMN016 CDCl3 24.9°C
o-tolyl product

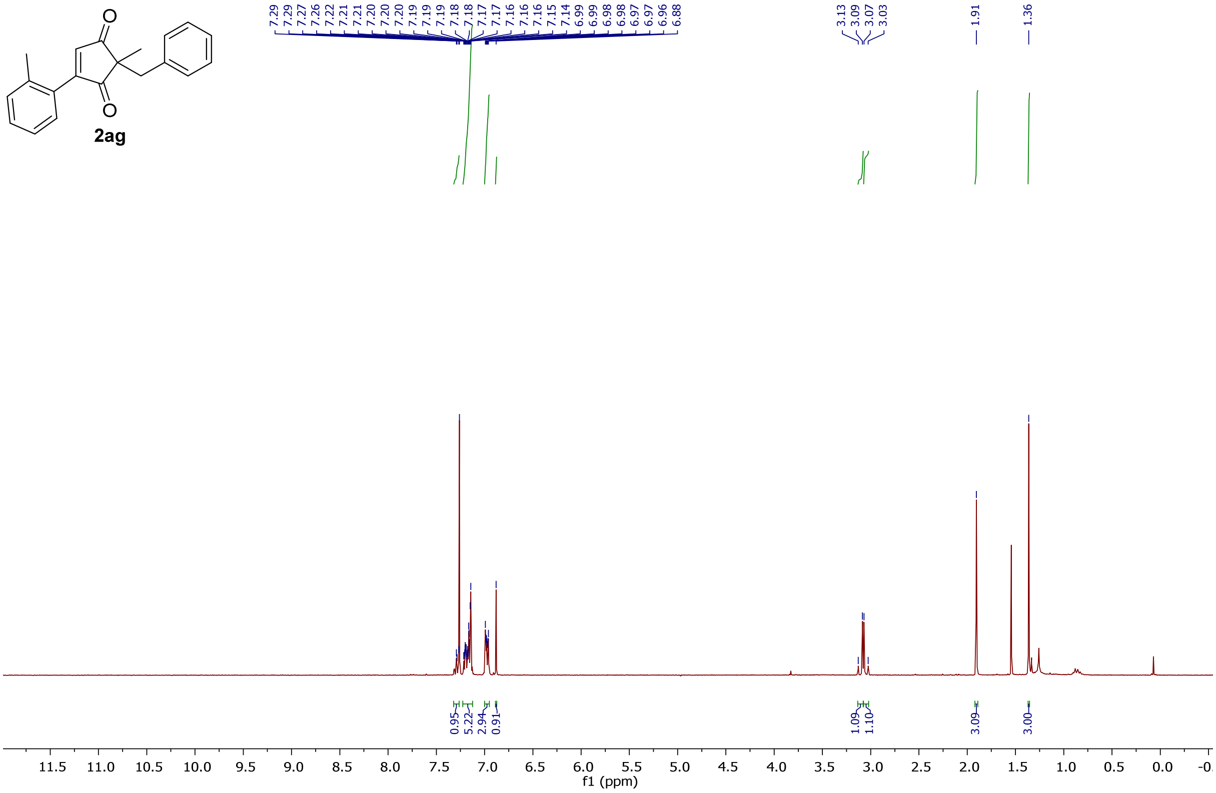

b5ncbmn023c.1.fid
13C 75.5MHz Job 62075 Nderitu Bryan G BMN023C CDCl3 25.0°C 3 hours 1 min
O-tolyl

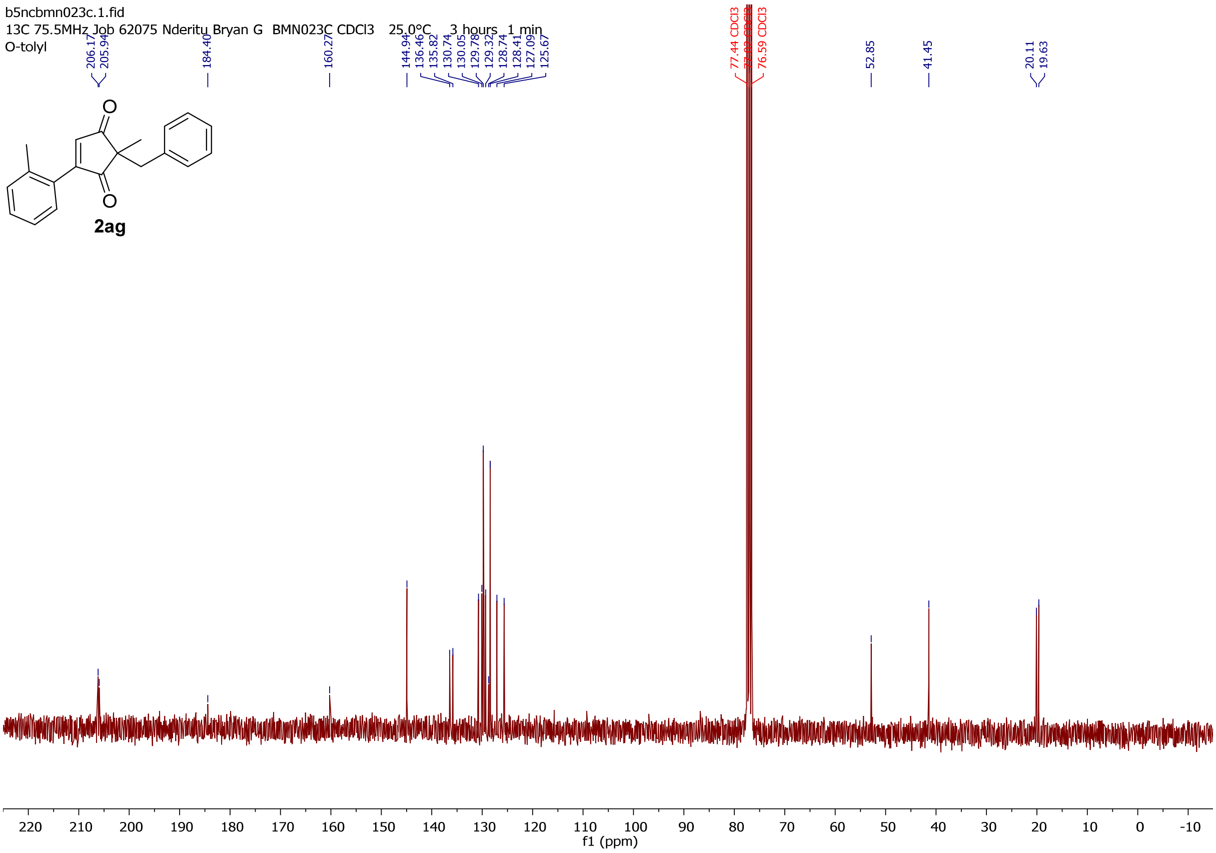

cjlh070a1.1.fid
1H 300.1MHz Job 62313 Lamb Claire J 070A1 CDCl3 25.0°C
Fractions 10-14

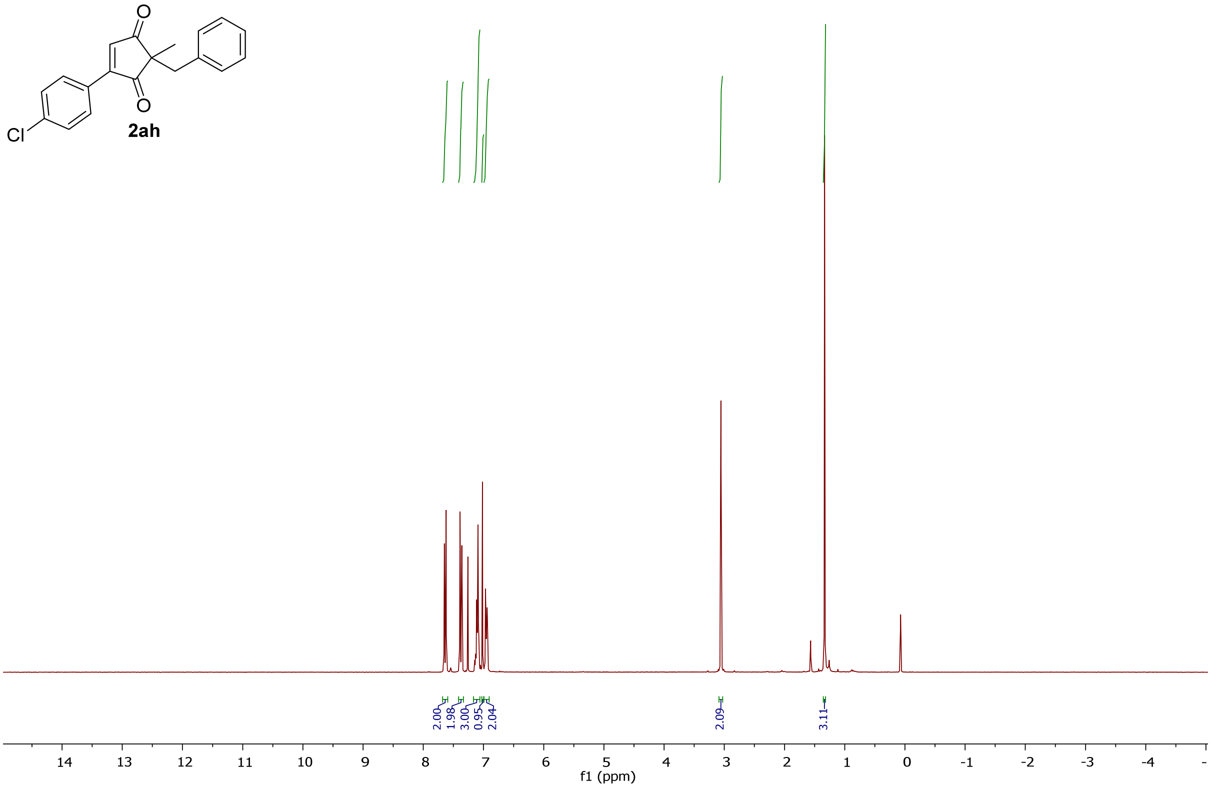

cjlc070ap.1.fid
13C 75.5MHz Job 62624 Lamb Claire J 070AP CDCl3 25.0°C 3 hours 1 min
*

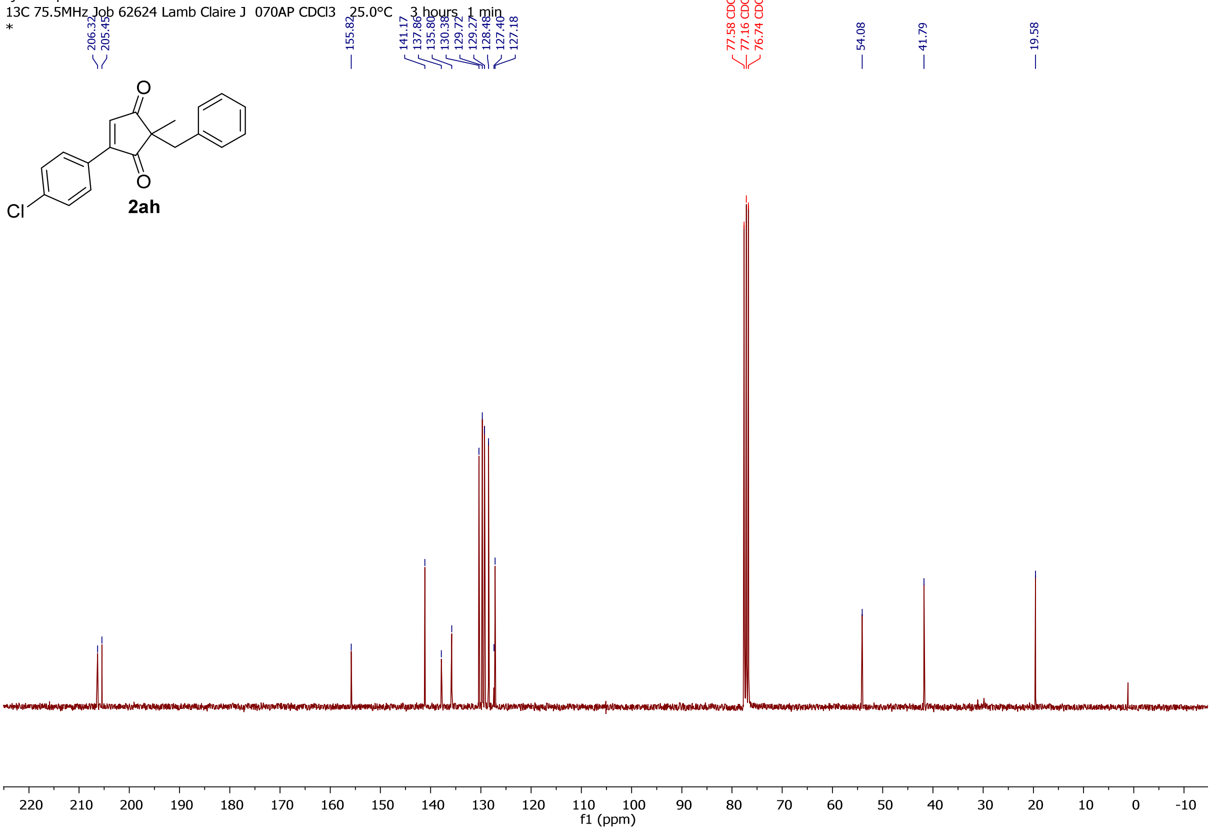

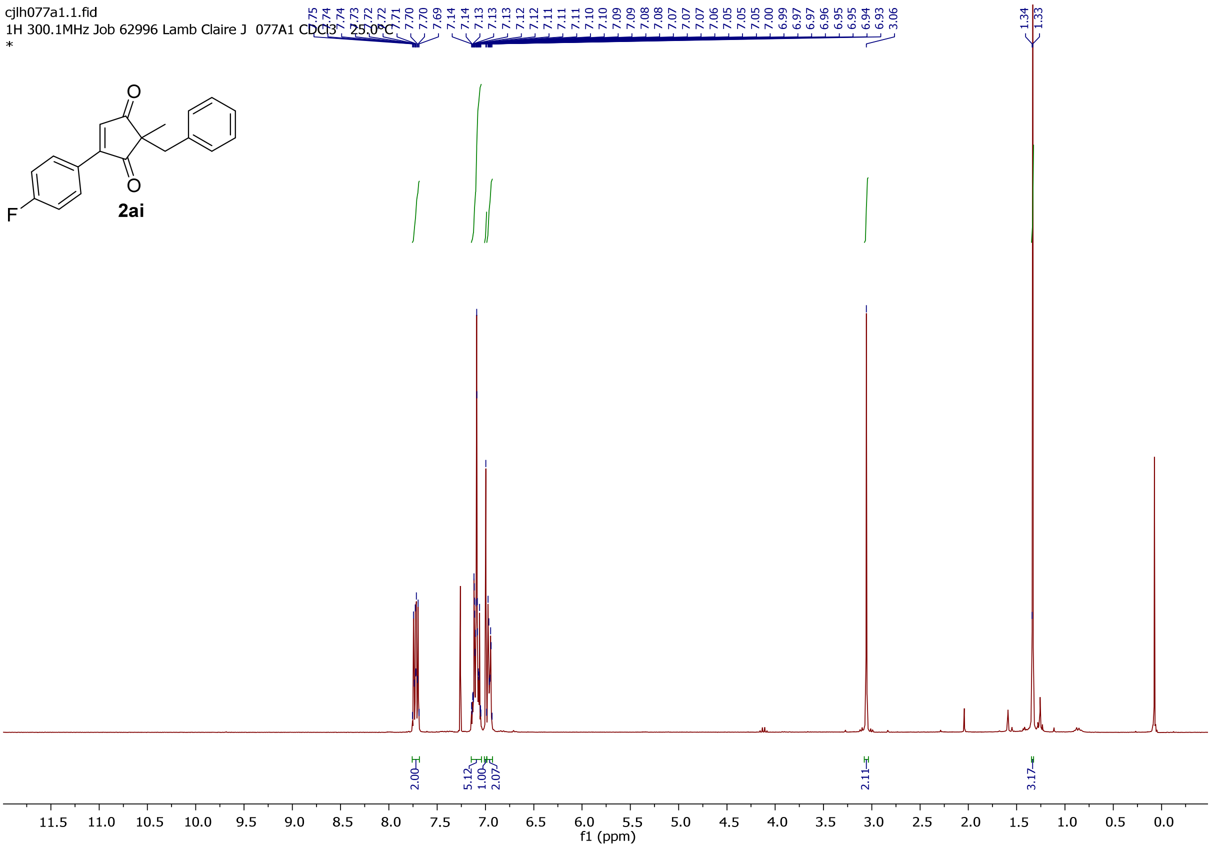
cjlh077a1.1.fid
1H 300.1MHz Job 62996 Lamb Claire J 077A1 CDCl3 25.0°C
*
7.75 7.74 7.74 7.73 7.72 7.72 7.71 7.70 7.70 7.69 7.14 7.13 7.13 7.13 7.12 7.11 7.11 7.10 7.09 7.09 7.08 7.08 7.07 7.07 7.07 7.06 7.05 7.05 7.05 7.00 6.99 6.97 6.97 6.96 6.95 6.95 6.94 6.93 3.06 1.34 1.33
F
O
O
2ai
2.00 5.12 1.00 2.07 2.11 3.17
11.5 11.0 10.5 10.0 9.5 9.0 8.5 8.0 7.5 7.0 6.5 6.0 5.5 5.0 4.5 4.0 3.5 3.0 2.5 2.0 1.5 1.0 0.5 0.0
f1 (ppm)

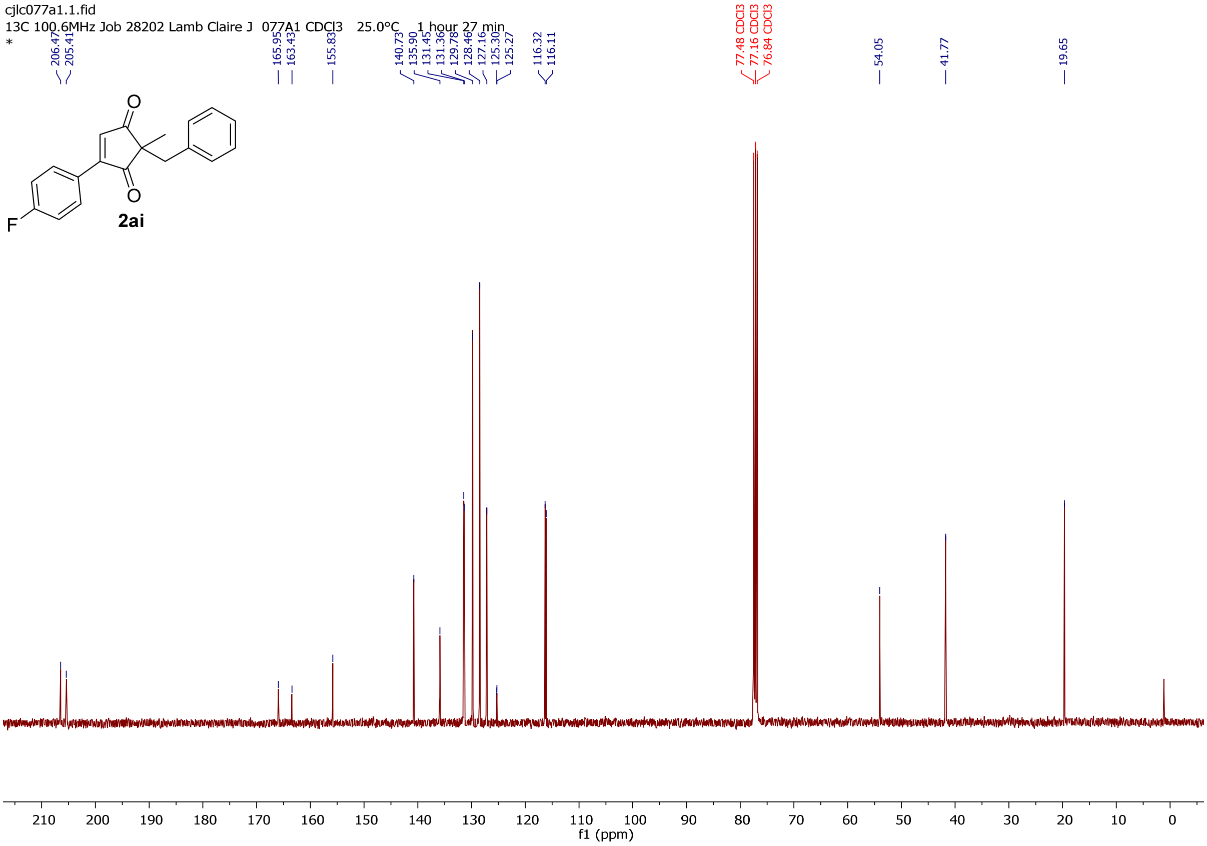
cjlc077a1.1.fid
13C 100.6MHz Job 28202 Lamb Claire J 077A1 CDCl3 25.0°C 1 hour 27 min
*
206.47 205.41 165.95 163.43 155.83 140.73 135.90 131.45 131.36 129.78 128.46 127.16 125.30 125.27 116.32 116.11 77.48 CDCl3 77.16 CDCl3 76.84 CDCl3 54.05 41.77 19.65
F
O
O
2ai
210 200 190 180 170 160 150 140 130 120 110 100 90 80 70 60 50 40 30 20 10 0
f1 (ppm)

cjlf077a1.1.fid
19F 376.5MHz Job 28197 Lamb Claire J 077A1 CDCl3 24.9°C 0hours 1min
*

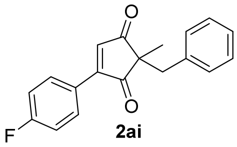

2ai

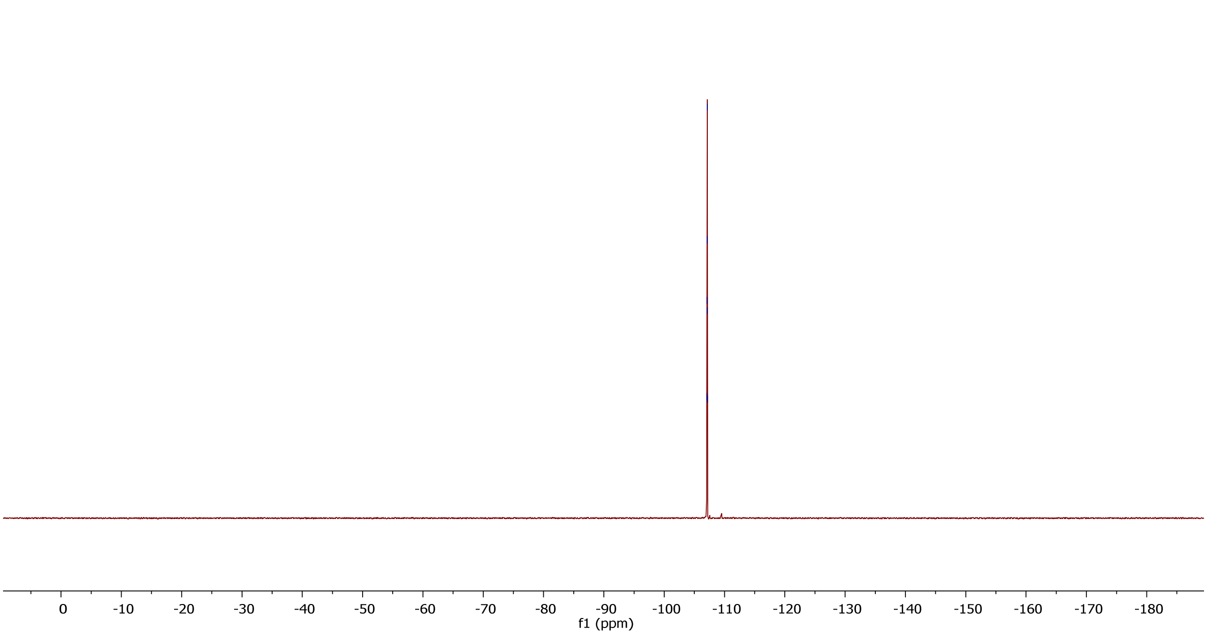

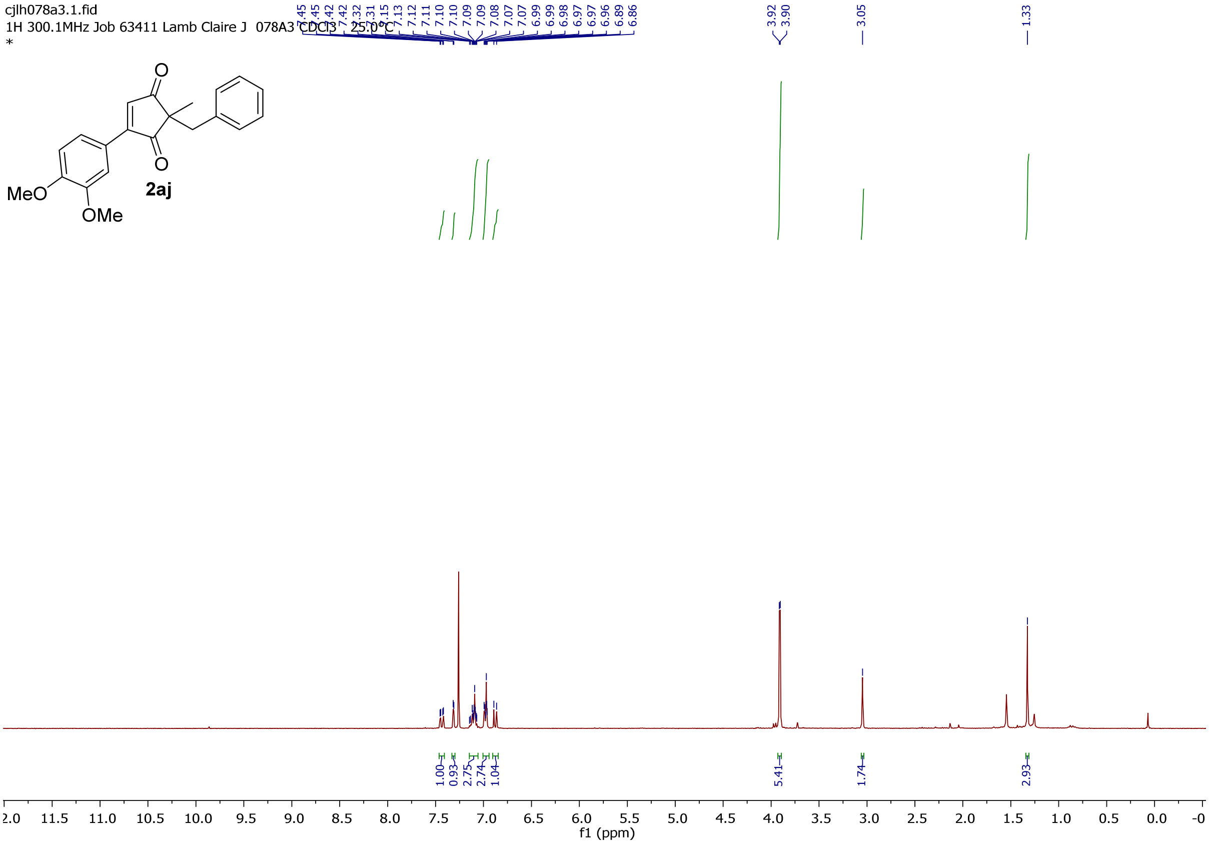
cjlh078a3.1.fid
1H 300.1MHz Job 63411 Lamb Claire J 078A3 CDCl3 25.0°C
*
7.45 7.45 7.42 7.32 7.31 7.15 7.13 7.12 7.11 7.10 7.09 7.09 7.08 7.07 7.07 6.99 6.99 6.98 6.97 6.97 6.96 6.89 6.86
3.92 3.90
3.05
1.33
O
O
MeO
OMe
2aj
1.00 0.93 2.75 2.74 1.04
5.41
1.74
2.93
f1 (ppm)

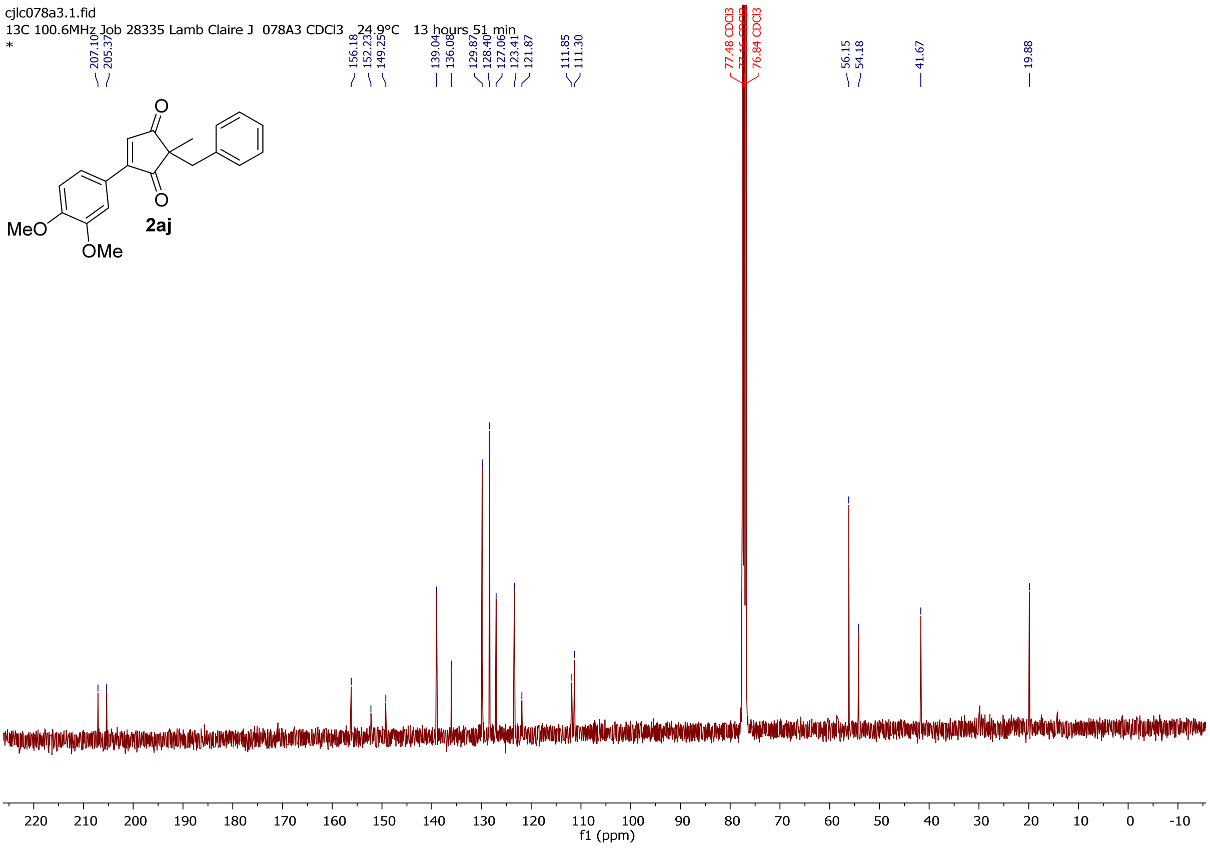
cjlc078a3.1.fid
13C 100.6MHz Job 28335 Lamb Claire J 078A3 CDCl3 24.9°C 13 hours 51 min
*
207.10 205.37
156.18 152.23 149.25
139.04 136.08 129.87 128.40 127.06 123.41 121.87
111.85 111.30
77.48 CDCl3
76.84 CDCl3
56.15 54.18
41.67
19.88
O
O
MeO
OMe
2aj
f1 (ppm)

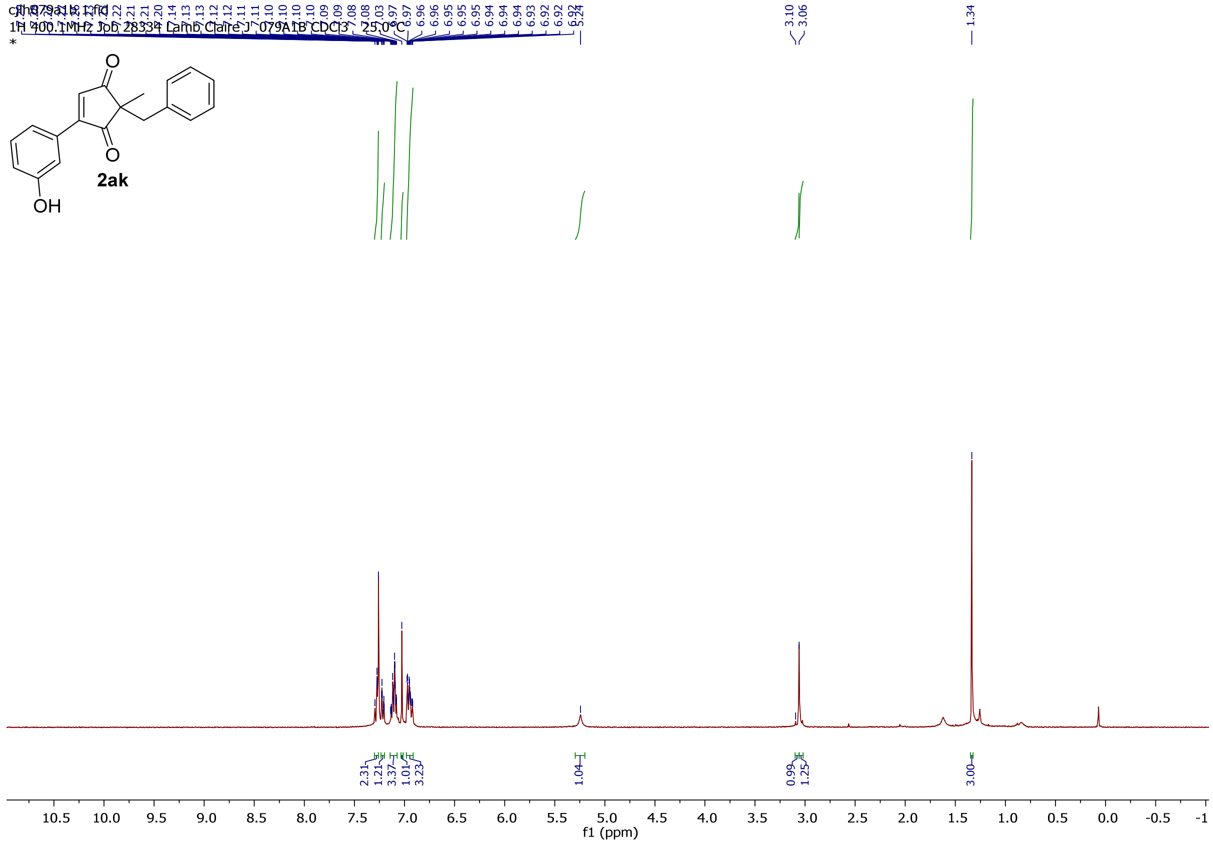
1H 400.1MHz Job 28334 Lamb Claire J 079A1B CDCl3 25.0°C
3.10
3.06
1.34
2ak
OH
O
2.31
1.21
3.37
1.01
3.23
1.04
0.99
1.25
3.00
f1 (ppm)

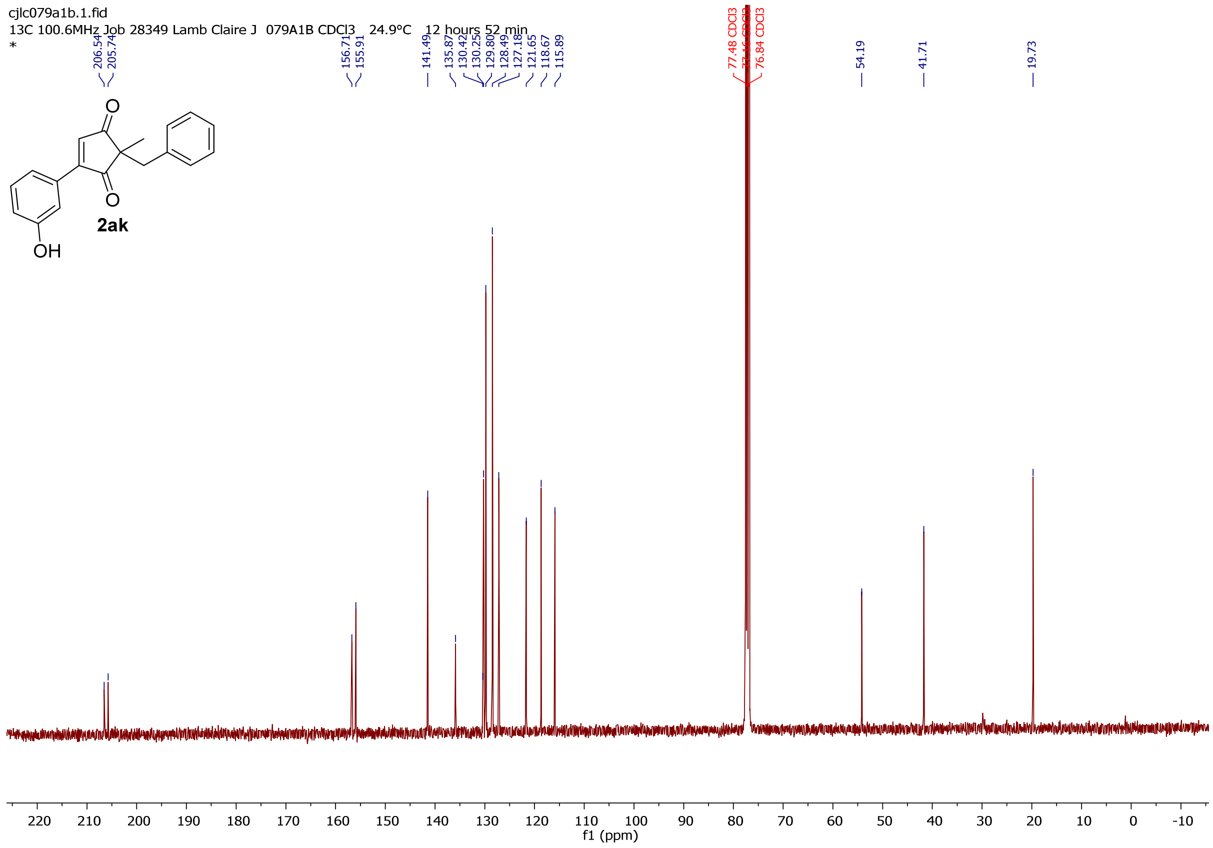
cjlc079a1b.1.fid
13C 100.6MHz Job 28349 Lamb Claire J 079A1B CDCl3 24.9°C 12 hours 52 min
206.54
205.74
156.71
155.91
141.49
135.87
130.42
130.25
129.80
128.49
127.18
121.65
118.67
115.89
77.48 CDCl3
76.84 CDCl3
54.19
41.71
19.73
2ak
OH
O
f1 (ppm)

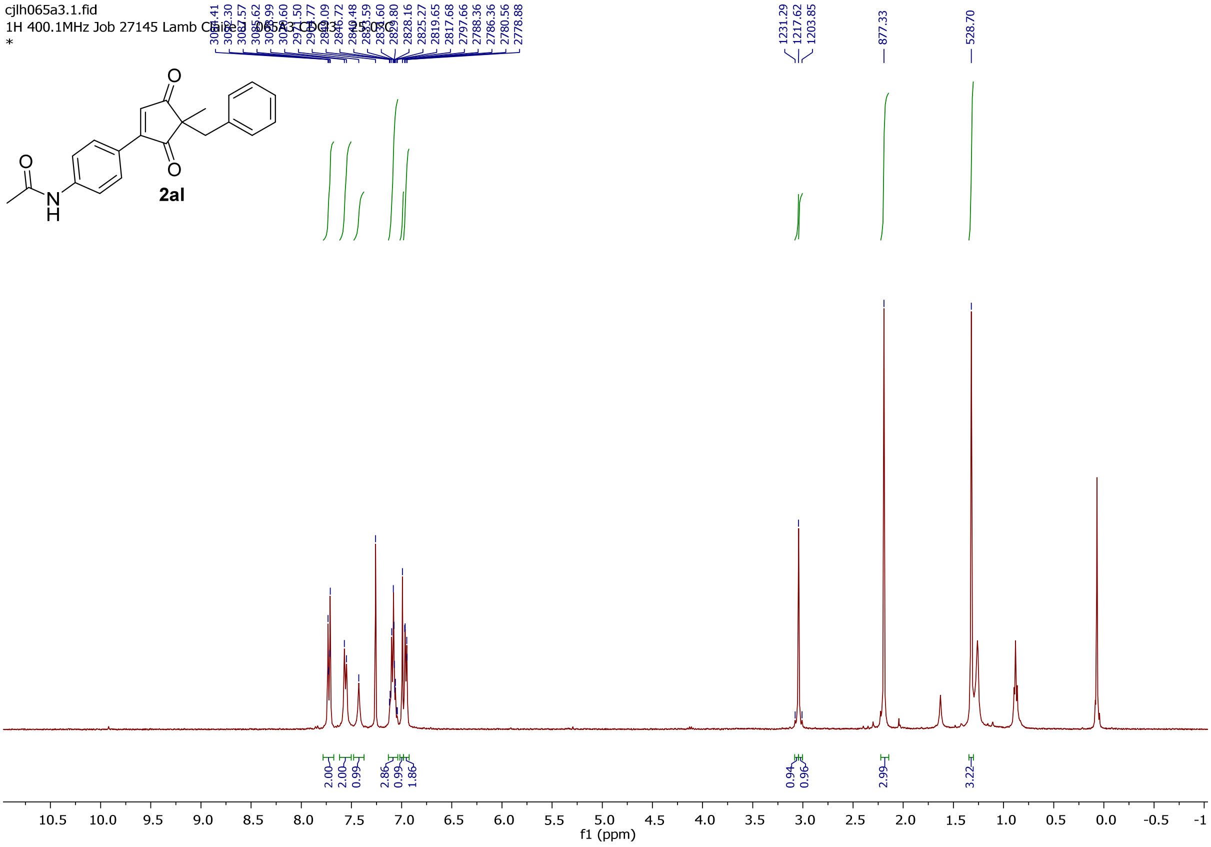
cjlh065a3.1.fid
1H 400.1MHz Job 27145 Lamb Claire J 065A3 CDCl3 25.0°C
*
1231.29
1217.62
1203.85
877.33
528.70
2al
2.00
2.00
0.99
2.86
0.99
1.86
0.94
0.96
2.99
3.22
f1 (ppm)

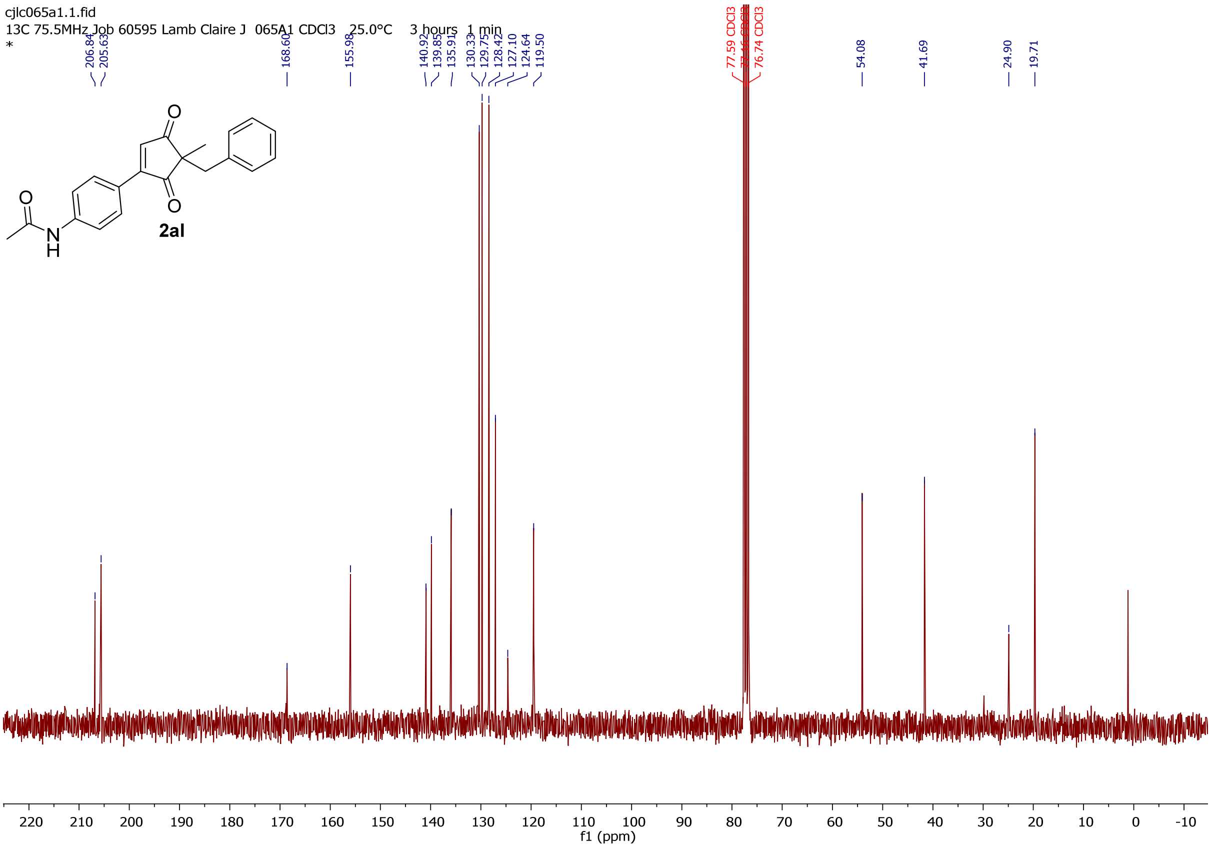
cjlc065a1.1.fid
13C 75.5MHz Job 60595 Lamb Claire J 065A1 CDCl3 25.0°C 3 hours 1 min
*
206.84
205.63
168.60
155.98
140.92
139.85
135.91
130.33
129.75
128.42
127.10
124.64
119.50
77.59 CDCl3
76.74 CDCl3
54.08
41.69
24.90
19.71
2al
f1 (ppm)

cjlh069b1.1.fid
1H 300.1MHz Job 62695 Lamb Claire J 069B1 CDCl3 24.9°C
*

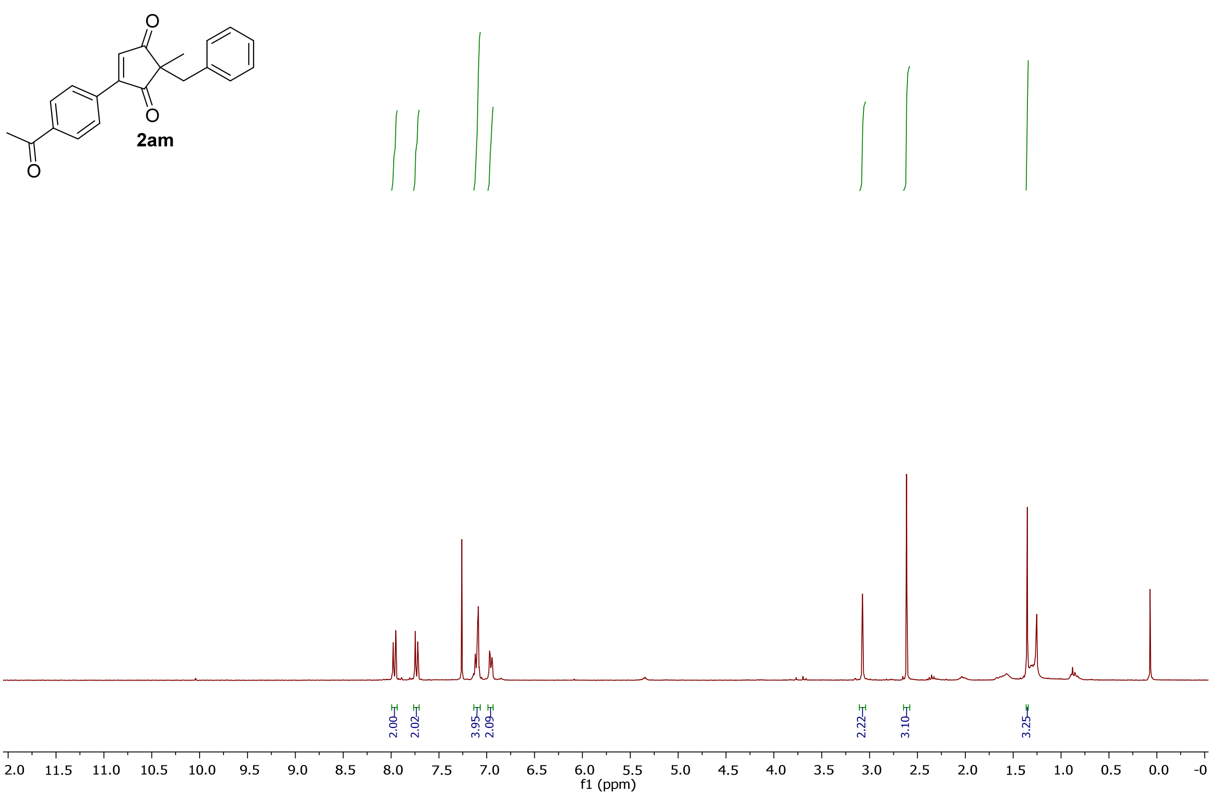

cjlc069b.1.fid
13C 100.6MHz Job 28385 Lamb Claire J 069B CDCl3 25.0°C 32 hours 48 min
*

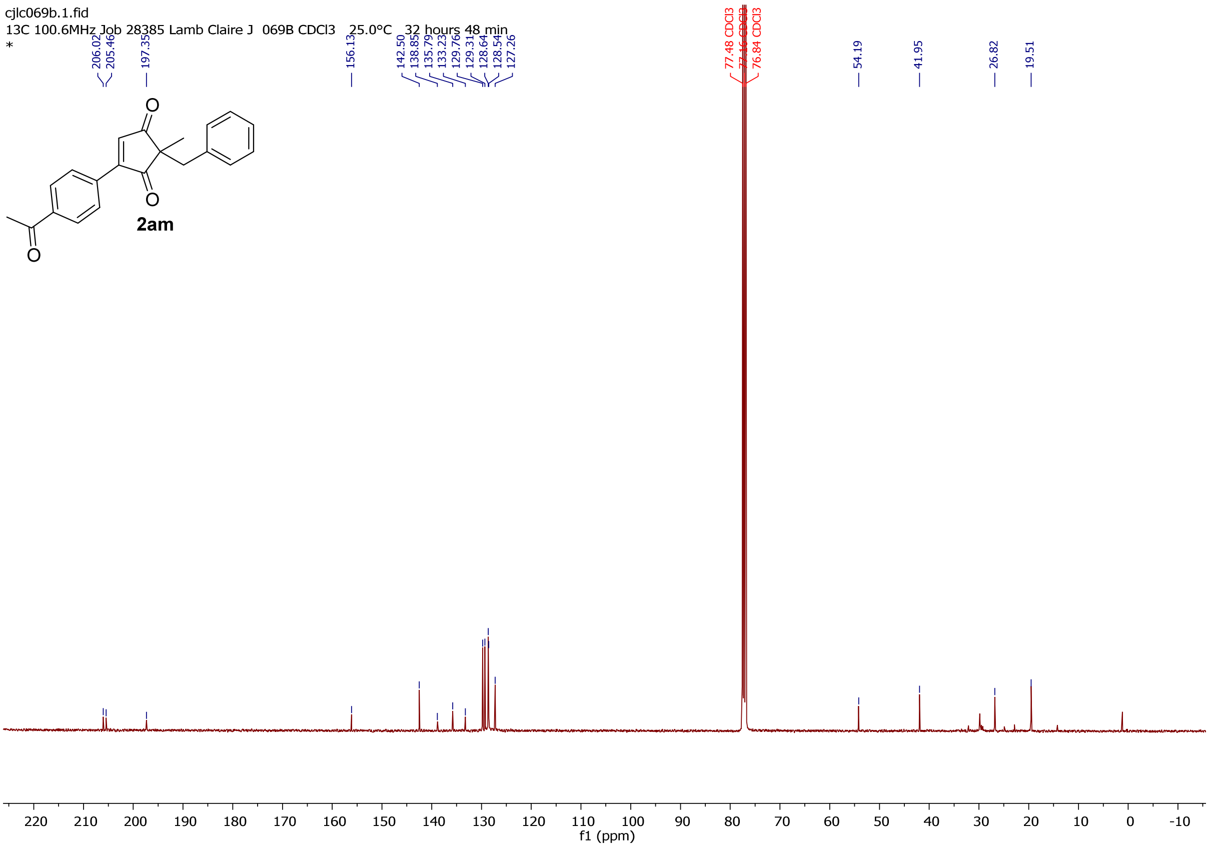

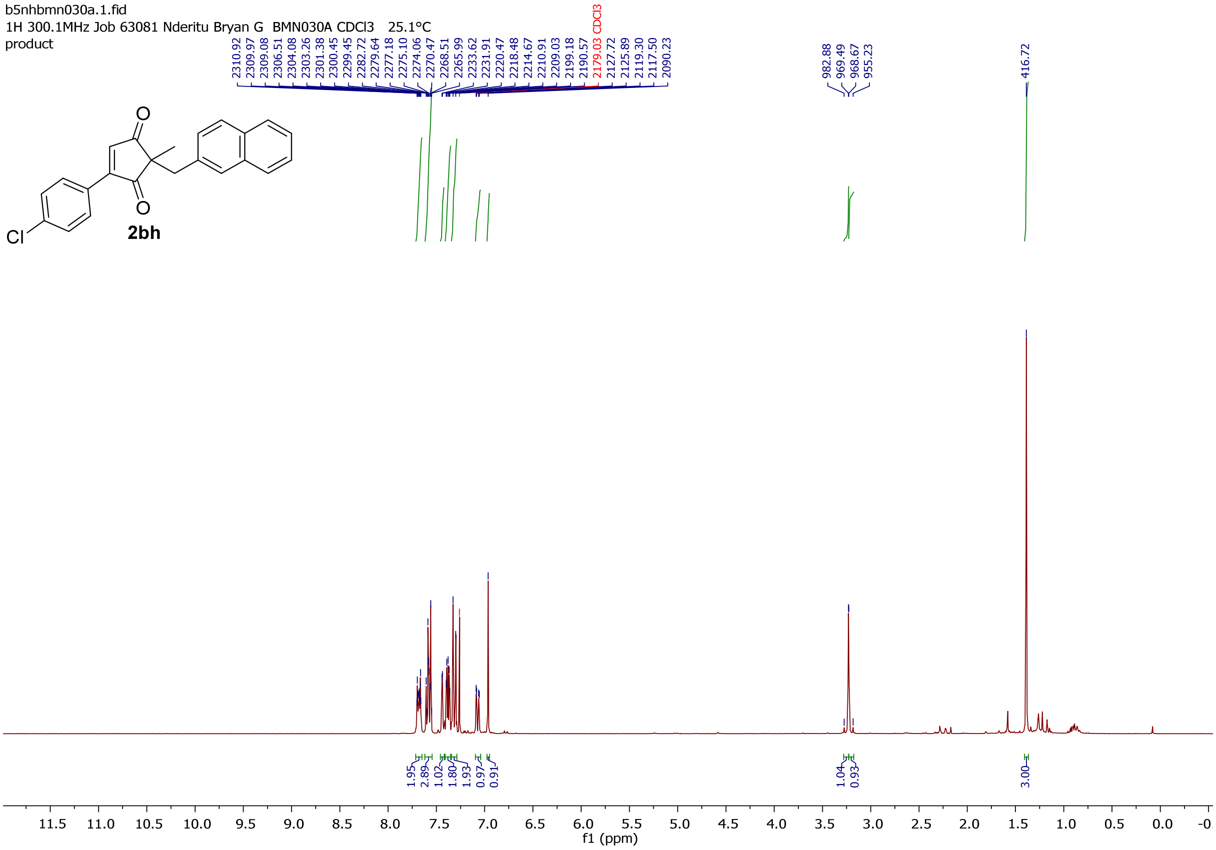
b5nhbmn030a.1.fid
1H 300.1MHz Job 63081 Nderitu Bryan G BMN030A CDCl3 25.1°C
product
2310.92 2309.97 2309.08 2306.51 2304.08 2303.26 2301.38 2300.45 2299.45 2282.72 2279.64 2277.18 2275.10 2274.06 2270.47 2268.51 2265.99 2233.62 2231.91 2220.47 2218.48 2214.67 2210.91 2209.03 2199.18 2190.57 2179.03 CDCl3 2127.72 2125.89 2119.30 2117.50 2090.23
982.88 969.49 968.67 955.23
416.72
2bh
1.95 2.89 1.02 1.80 1.93 0.97 0.91
1.04 0.93
3.00
f1 (ppm)

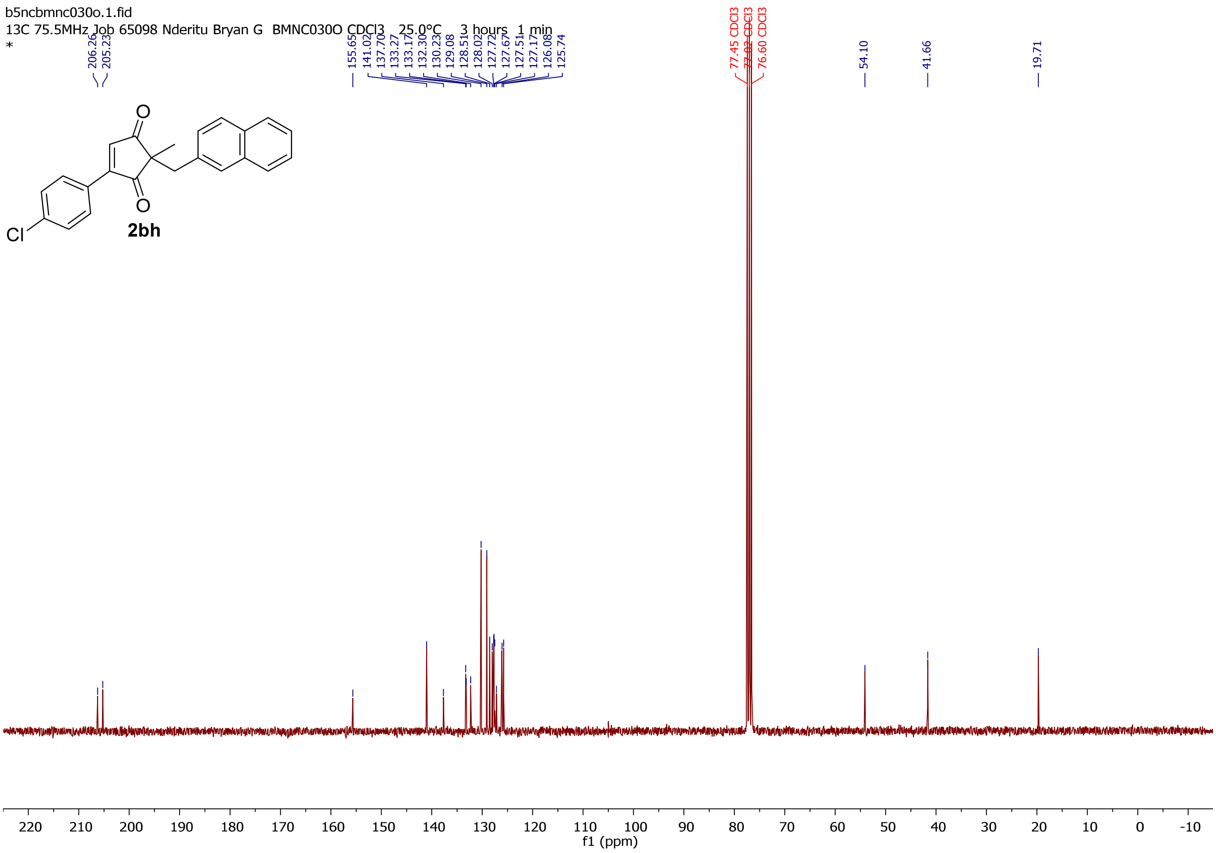
b5ncbmnc030o.1.fid
13C 75.5MHz Job 65098 Nderitu Bryan G BMNC030O CDCl3 25.0°C 3 hours 1 min
*
206.26 205.23
155.65 141.02 137.70 133.27 133.17 132.30 130.23 129.08 128.51 128.02 127.72 127.67 127.51 127.17 126.08 125.74
77.45 CDCl3 77.02 CDCl3 76.60 CDCl3
54.10
41.66
19.71
2bh
f1 (ppm)

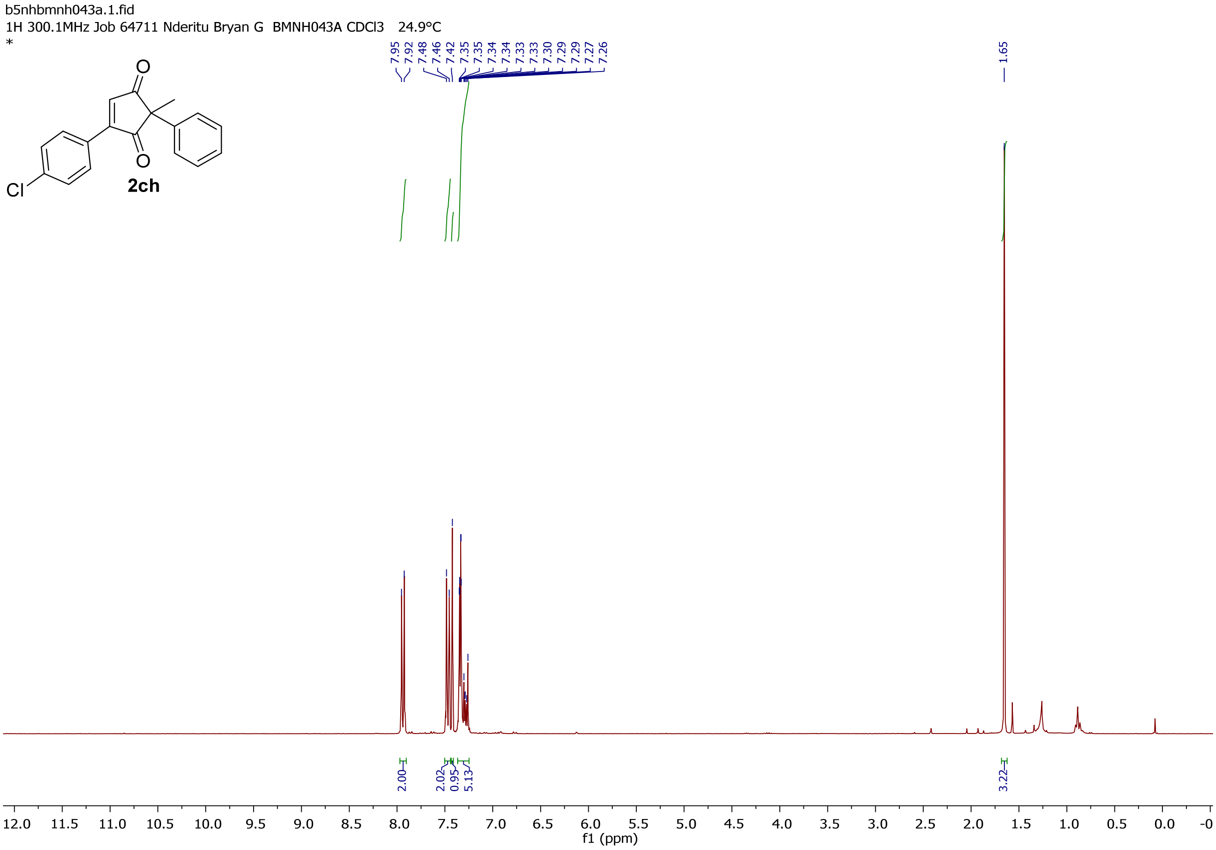
b5nhbmnh043a.1.fid
1H 300.1MHz Job 64711 Nderitu Bryan G BMNH043A CDCl3 24.9°C
*
7.95 7.92 7.48 7.46 7.42 7.35 7.35 7.34 7.34 7.33 7.33 7.30 7.29 7.29 7.27 7.26
1.65
Cl
2ch
2.00 2.02 0.95 5.13
3.22
f1 (ppm)

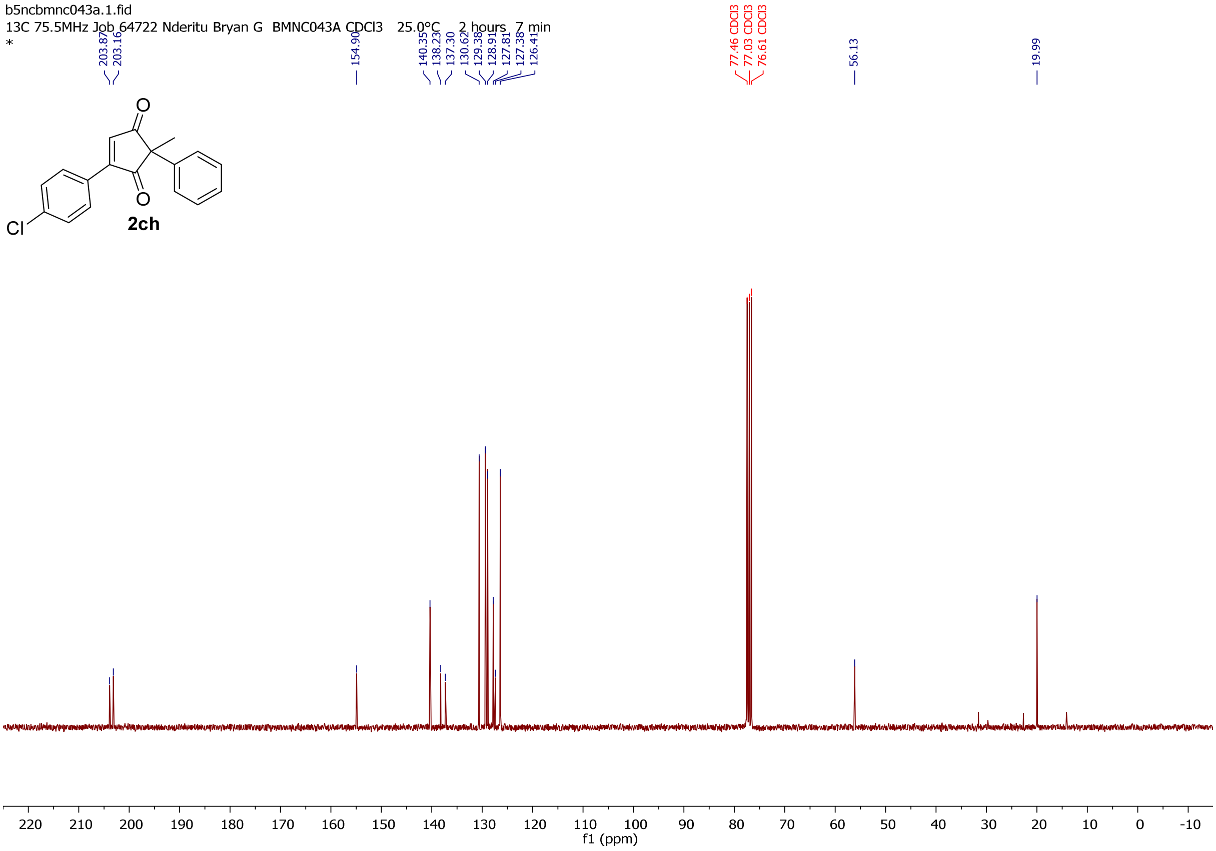
b5ncbmnc043a.1.fid
13C 75.5MHz Job 64722 Nderitu Bryan G BMNC043A CDCl3 25.0°C 2 hours 7 min
*
203.87 203.16
154.90
140.35 138.23 137.30 130.62 129.38 128.91 127.81 127.38 126.41
77.46 CDCl3 77.03 CDCl3 76.61 CDCl3
56.13
19.99
Cl
2ch
f1 (ppm)

b5nhbmnh037a.1.fid
1H 300.1MHz Job 64540 Nderitu Bryan G BMNH037A CDCl3 24.9°C
*

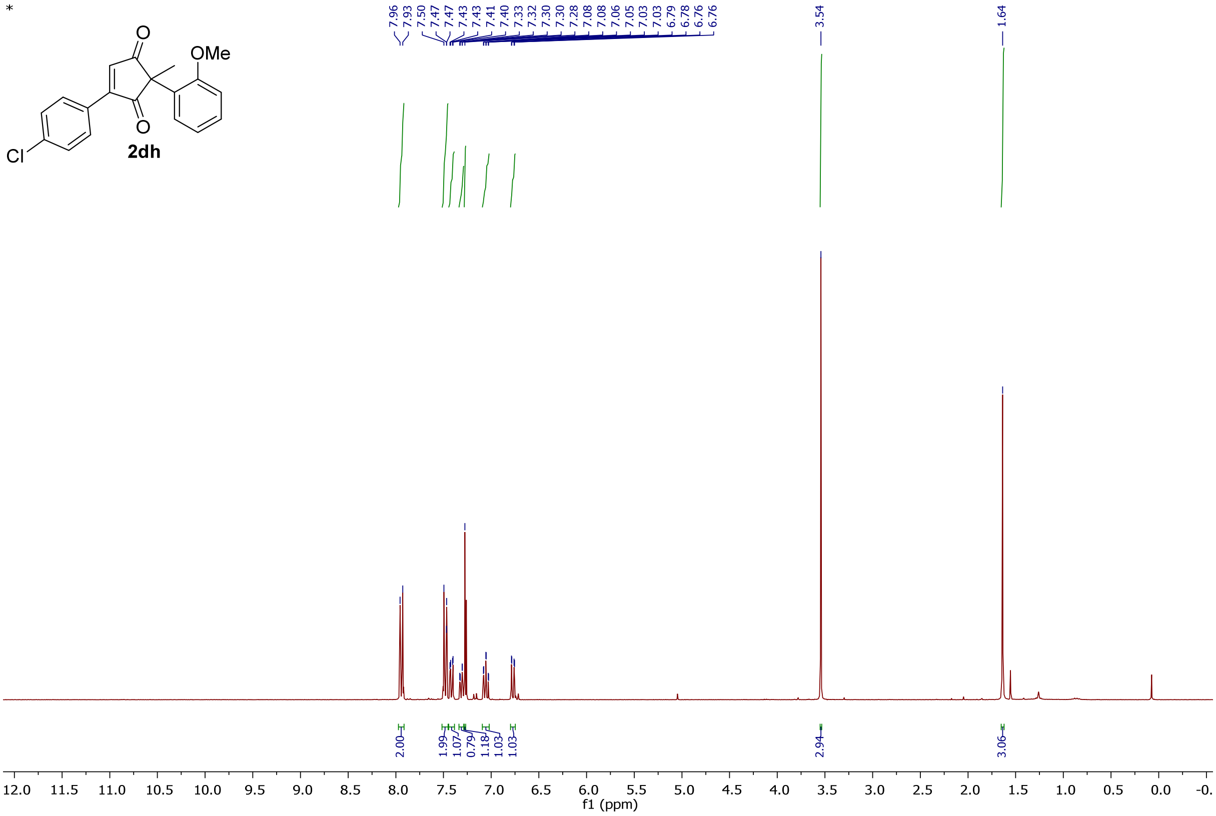

b5ncbmnc037a.1.fid
13C 75.5MHz Job 64594 Nderitu Bryan G BMNC037A CDCl3 25.0°C 3 hours 1 min
*

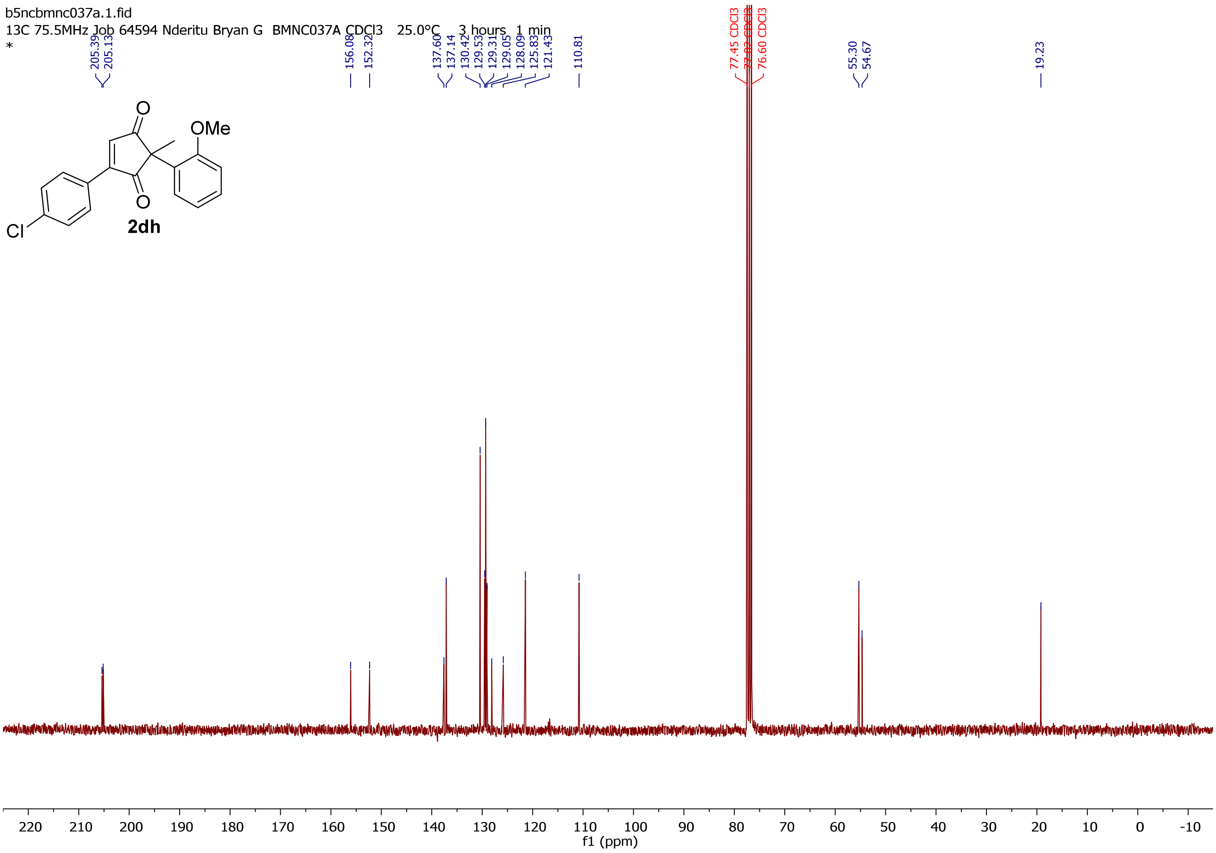

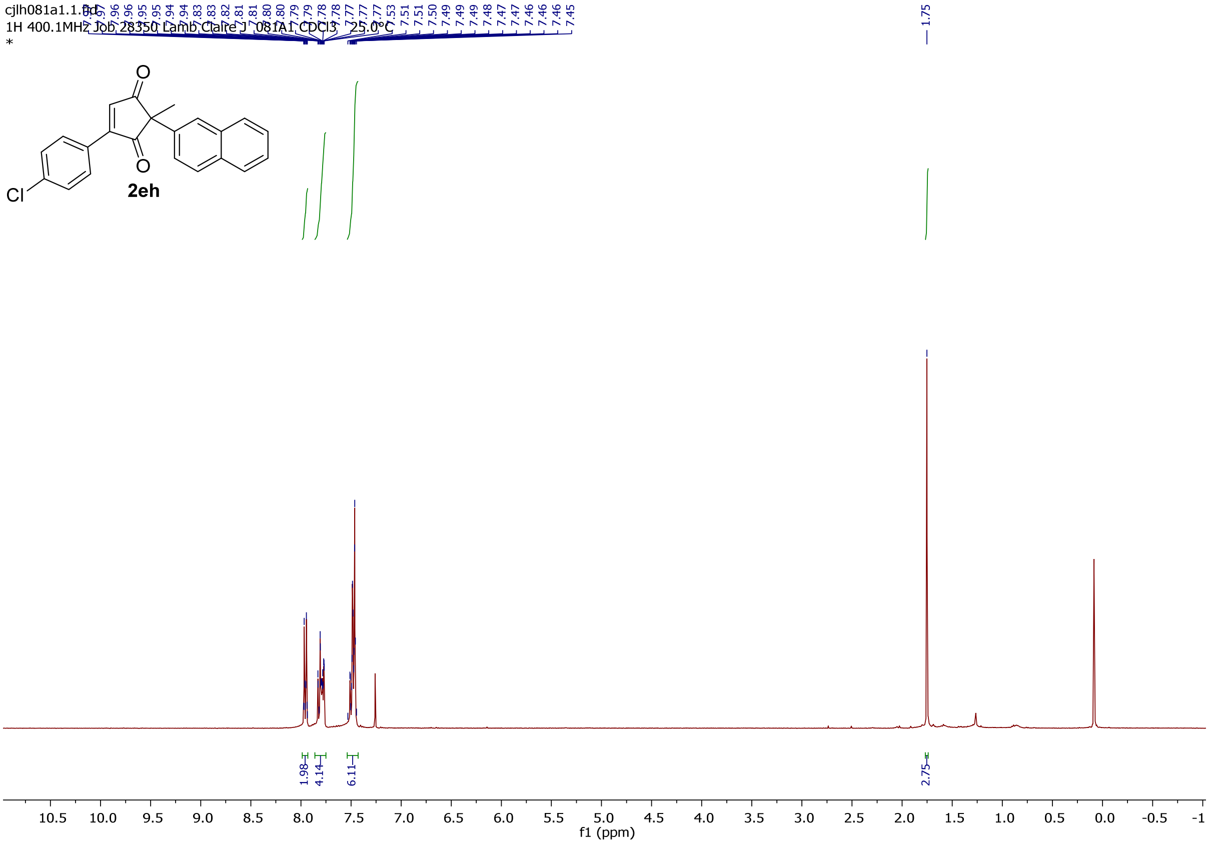

cjlh081a1.1.fid
1H 400.1MHz Job 28350 Lamb Claire J 081A1 CDCl3 25.0°C
7.96 7.95 7.95 7.94 7.94 7.83 7.83 7.82 7.81 7.81 7.80 7.80 7.79 7.79 7.78 7.78 7.77 7.77 7.77 7.53 7.51 7.51 7.50 7.49 7.49 7.49 7.48 7.47 7.47 7.46 7.46 7.46 7.45
1.75
O
O
Cl
2eh
1.98
4.14
6.11
2.75
f1 (ppm)

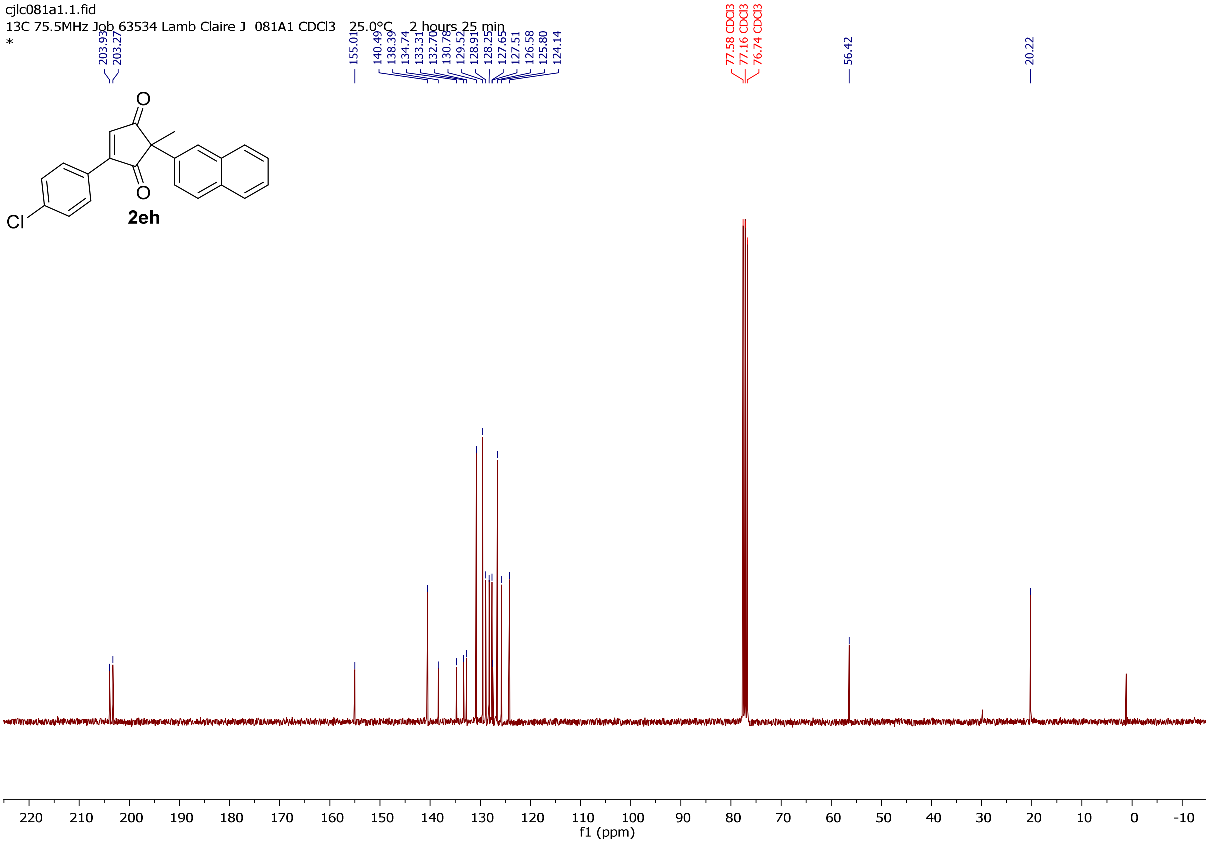

cjlc081a1.1.fid
13C 75.5MHz Job 63534 Lamb Claire J 081A1 CDCl3 25.0°C 2 hours 25 min
203.93
203.27
155.01
140.49
138.39
134.74
133.31
132.70
130.78
129.52
128.91
128.25
127.65
127.51
126.58
125.80
124.14
77.58 CDCl3
77.16 CDCl3
76.74 CDCl3
56.42
20.22
O
O
Cl
2eh
f1 (ppm)

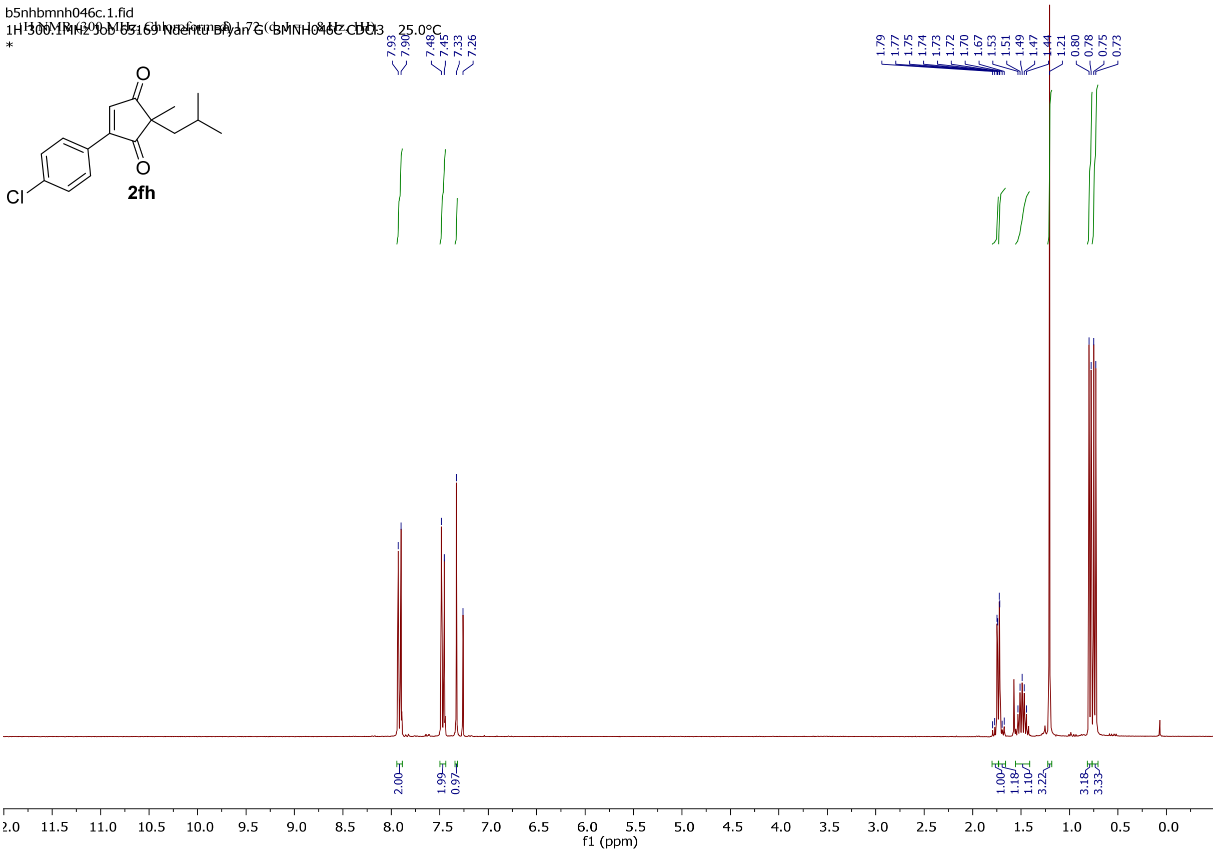

b5nhbmnh046c.1.fid
1H 300.1MHz Job 65169 Nderitu Bryan G BMNH046C CDCl3 25.0°C
*
7.93
7.90
7.48
7.45
7.33
7.26
1.79
1.77
1.75
1.74
1.73
1.72
1.70
1.67
1.53
1.51
1.49
1.47
1.44
1.21
0.80
0.78
0.75
0.73
O
O
Cl
2fh
2.00
1.99
0.97
1.00
1.18
1.10
3.22
3.18
3.33
f1 (ppm)

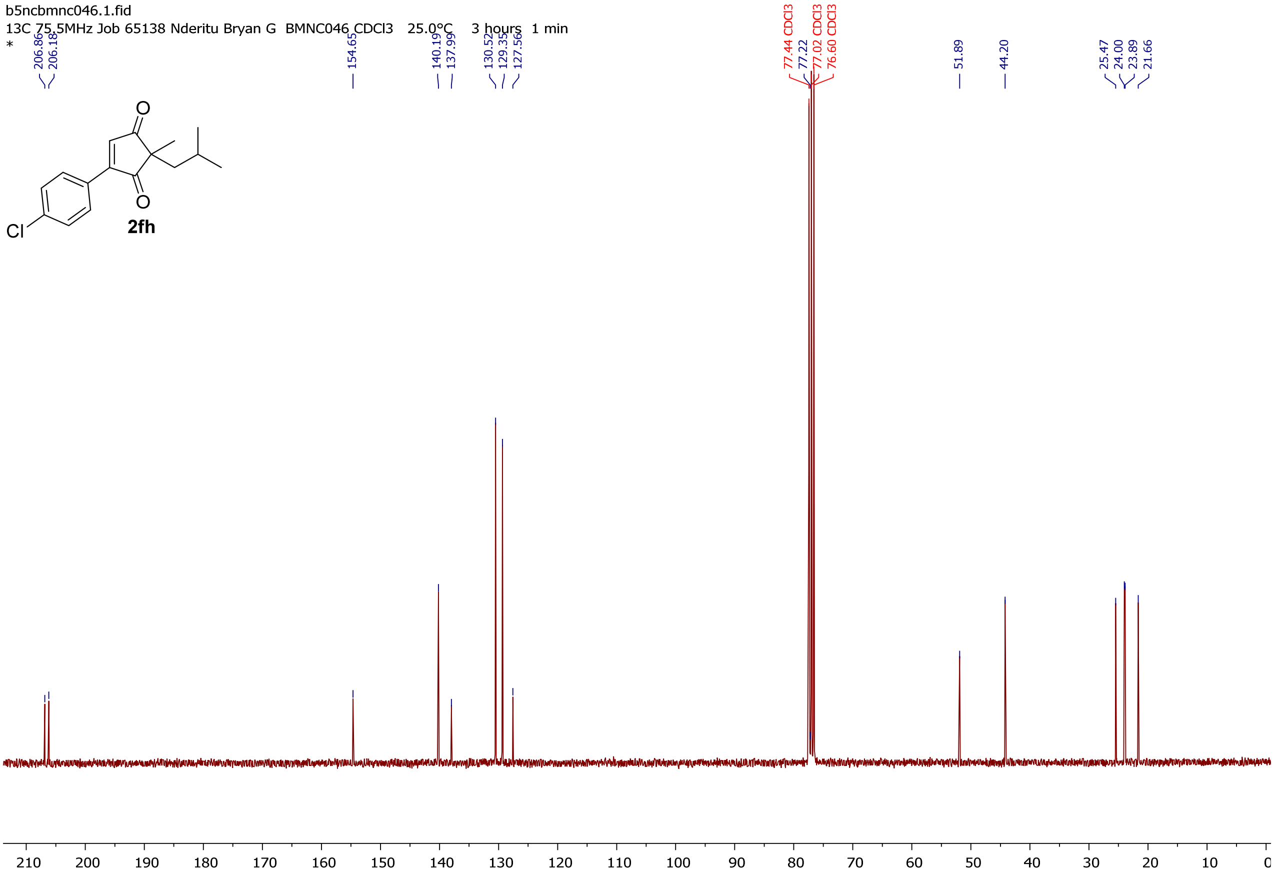

b5ncbmnc046.1.fid
13C 75.5MHz Job 65138 Nderitu Bryan G BMNC046 CDCl3 25.0°C 3 hours 1 min
*
206.86
206.18
154.65
140.19
137.99
130.52
129.35
127.56
77.44 CDCl3
77.22
77.02 CDCl3
76.60 CDCl3
51.89
44.20
25.47
24.00
23.89
21.66
O
O
Cl
2fh
f1 (ppm)

b5nhbmnh038d.1.fid
1H 300.1MHz Job 64653 Nderitu Bryan G BMNH038D CDCl3 24.9°C
*

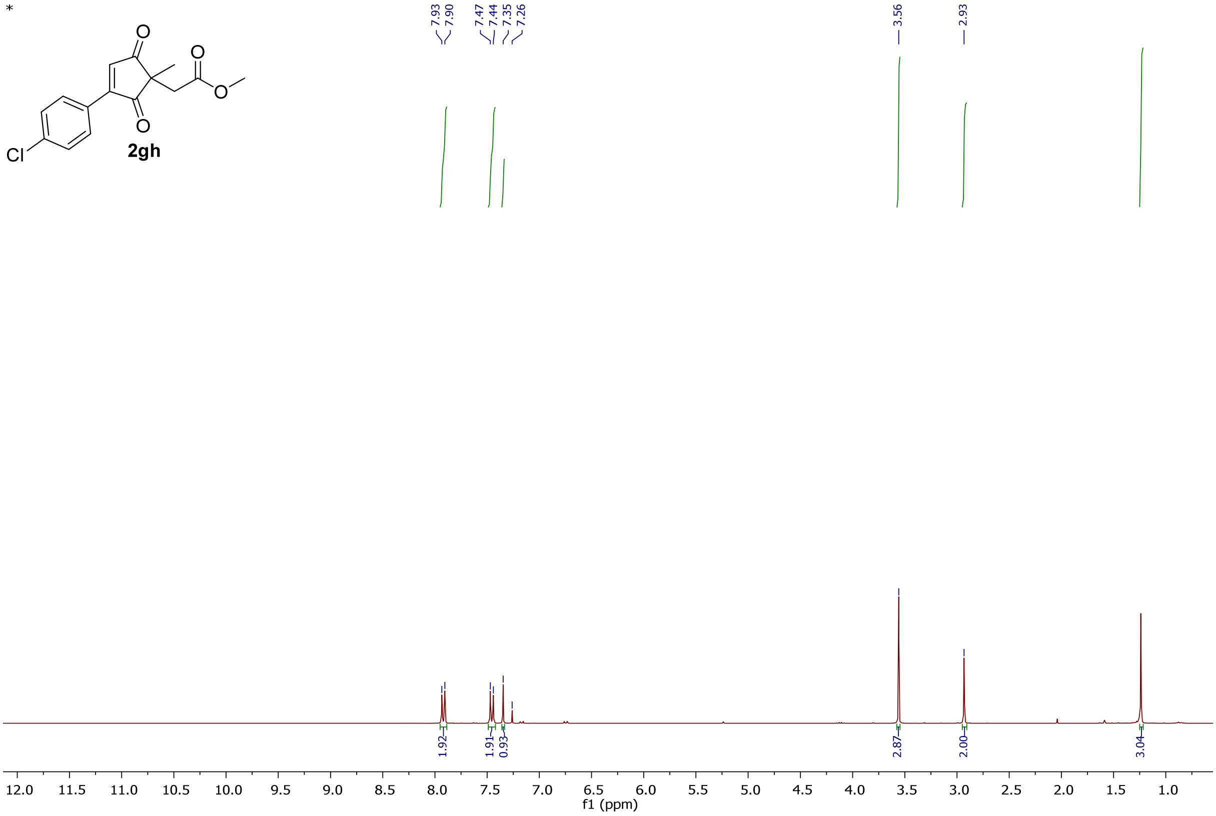

b5ncbmnc038c.1.fid
13C 75.5MHz Job 64721 Nderitu Bryan G BMNC038C CDCl3 25.0°C 3 hours 1 min
*

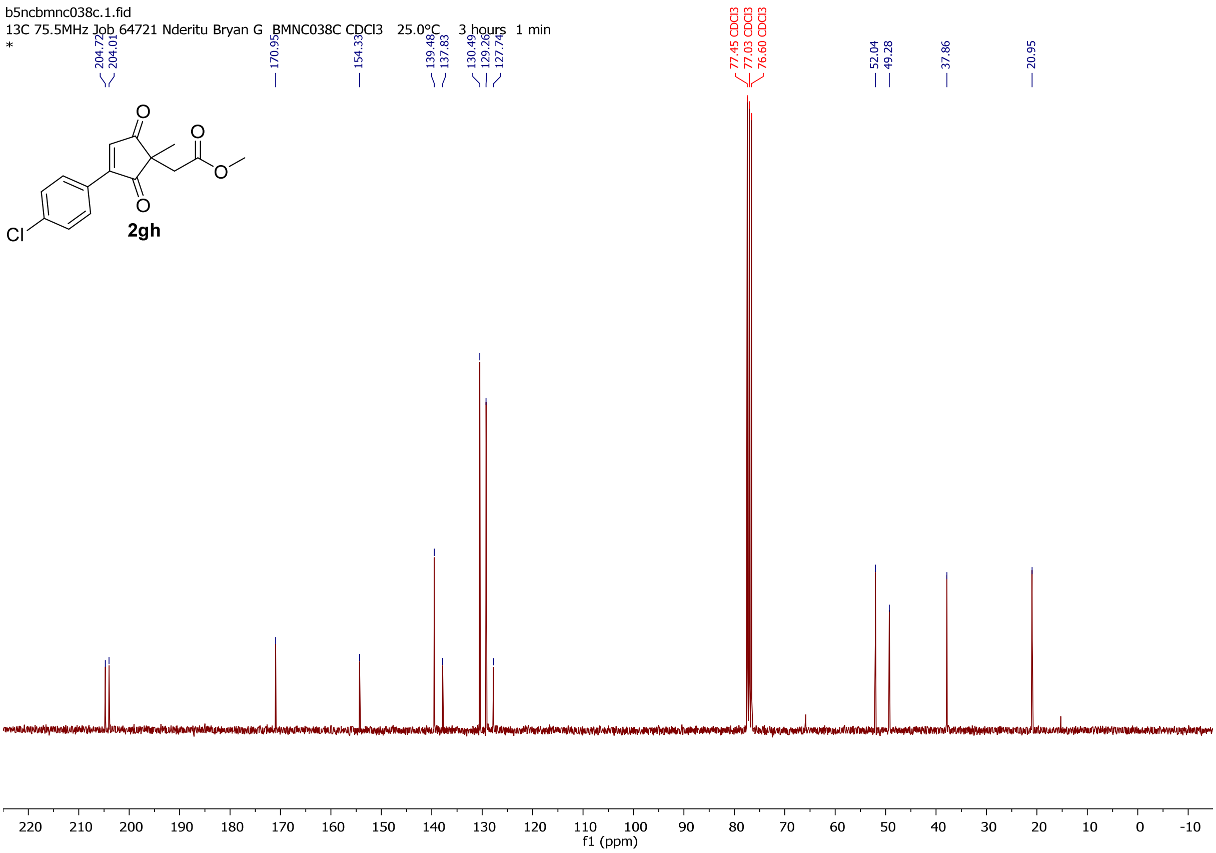

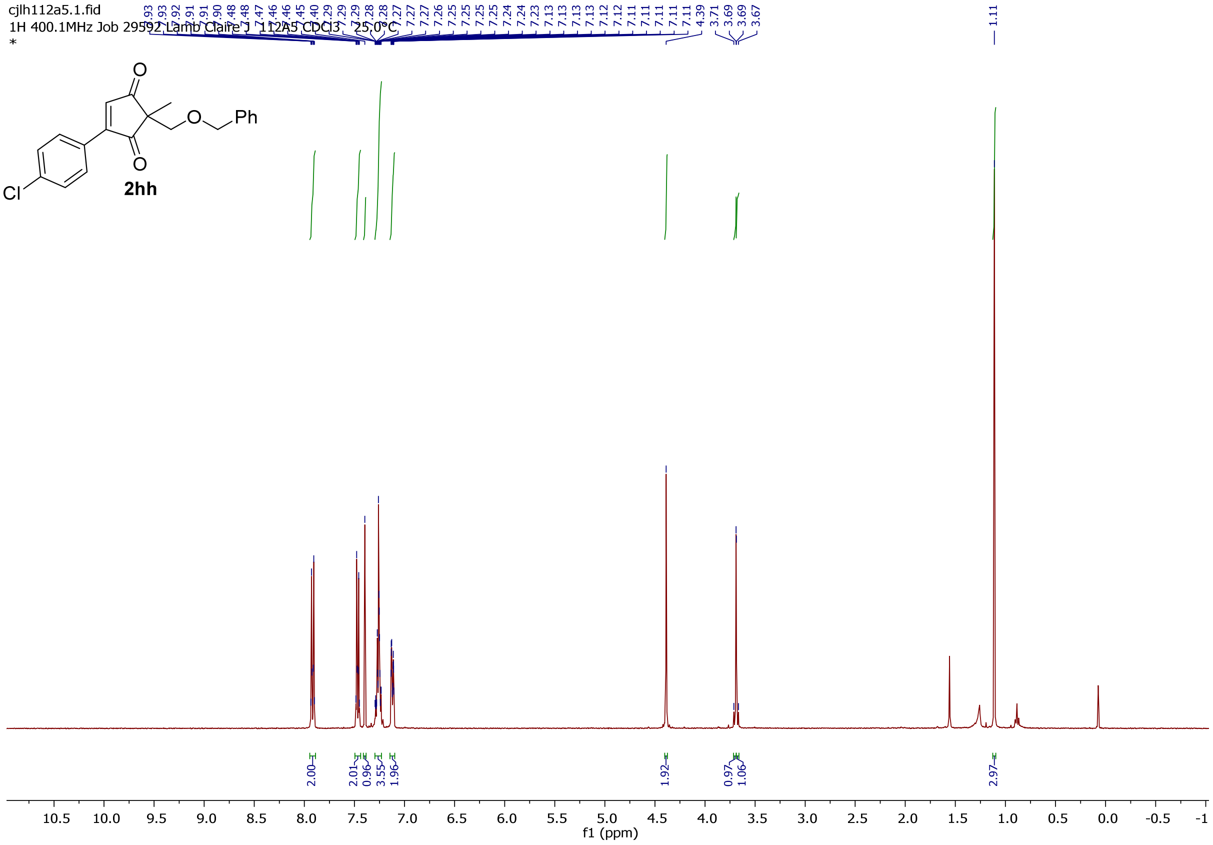
cjlh112a5.1.fid
1H 400.1MHz Job 29592 Lamb Claire J 112A5 CDCl3 25.0°C
*
O
O
Ph
Cl
2hh
4.39
3.71
3.69
3.69
3.67
1.11
2.00
2.01
0.96
3.55
1.96
1.92
0.97
1.06
2.97
f1 (ppm)

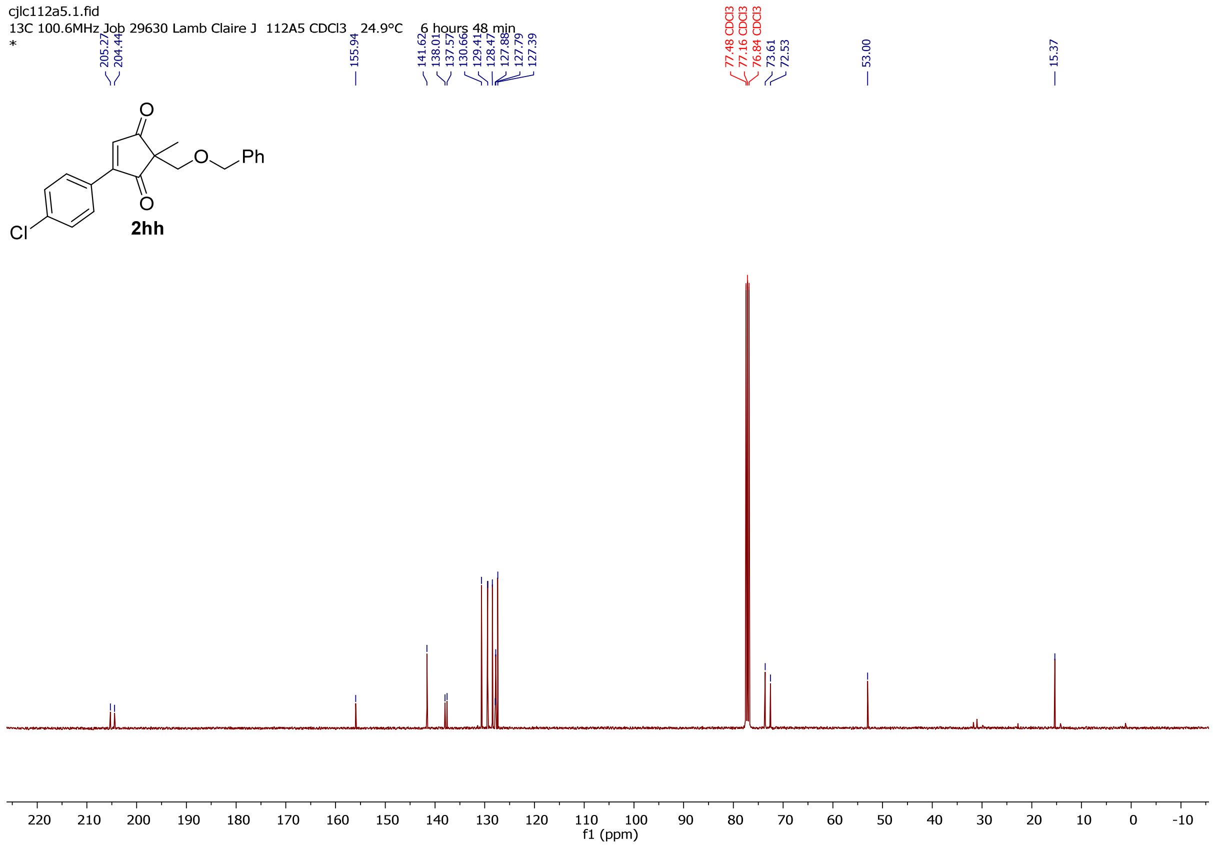
cjlc112a5.1.fid
13C 100.6MHz Job 29630 Lamb Claire J 112A5 CDCl3 24.9°C 6 hours 48 min
*
205.27
204.44
155.94
141.62
138.01
137.57
130.66
129.41
128.47
127.88
127.79
127.39
77.48 CDCl3
77.16 CDCl3
76.84 CDCl3
73.61
72.53
53.00
15.37
O
O
Ph
Cl
2hh
f1 (ppm)

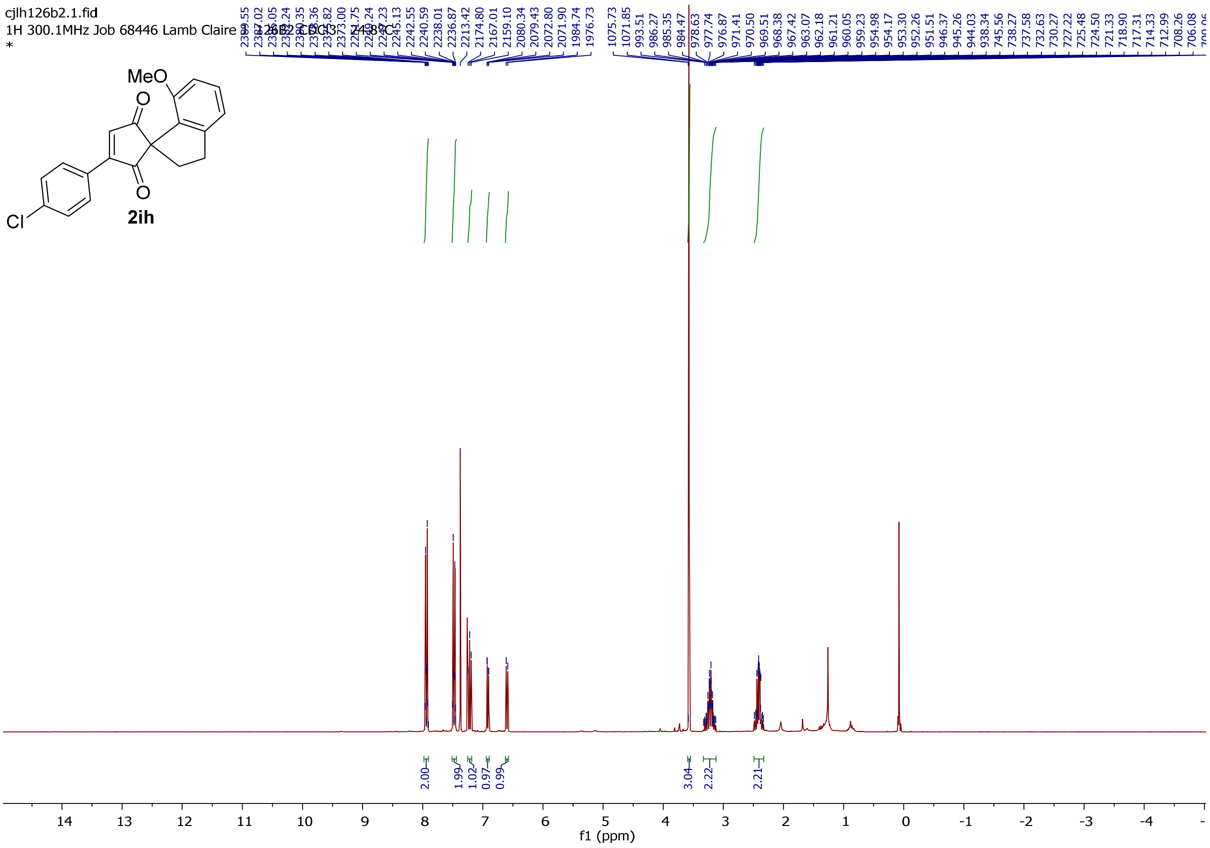

cjlh126b2.1.fid
1H 300.1MHz Job 68446 Lamb Claire J 126B2 CDCl3
MeO
O
O
Cl
2ih
2.00
1.99
1.02
0.97
0.99
3.04
2.22
2.21
f1 (ppm)

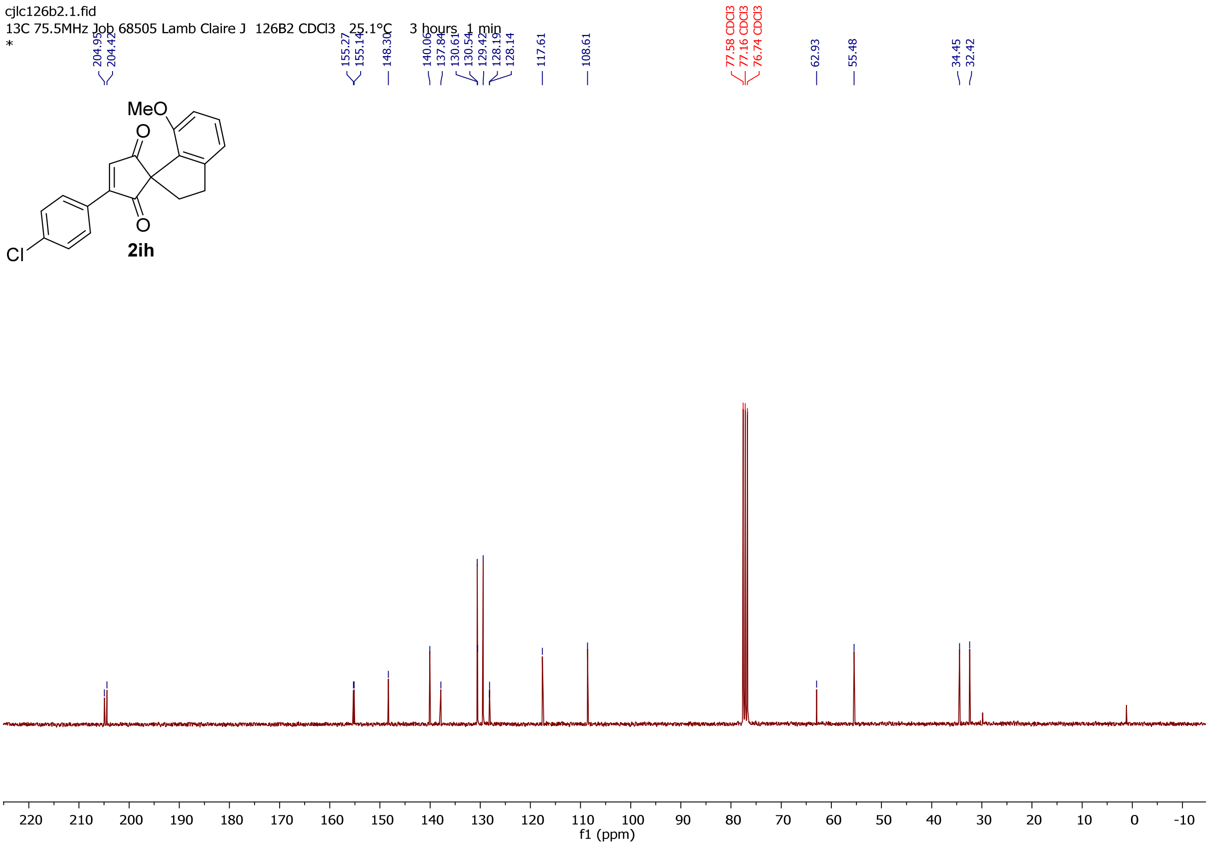

cjlc126b2.1.fid
13C 75.5MHz Job 68505 Lamb Claire J 126B2 CDCl3 25.1°C 3 hours 1 min
204.95
204.42
155.27
155.14
148.30
140.06
137.84
130.61
130.54
129.42
128.19
128.14
117.61
108.61
77.58 CDCl3
77.16 CDCl3
76.74 CDCl3
62.93
55.48
34.45
32.42
MeO
O
O
Cl
2ih
f1 (ppm)

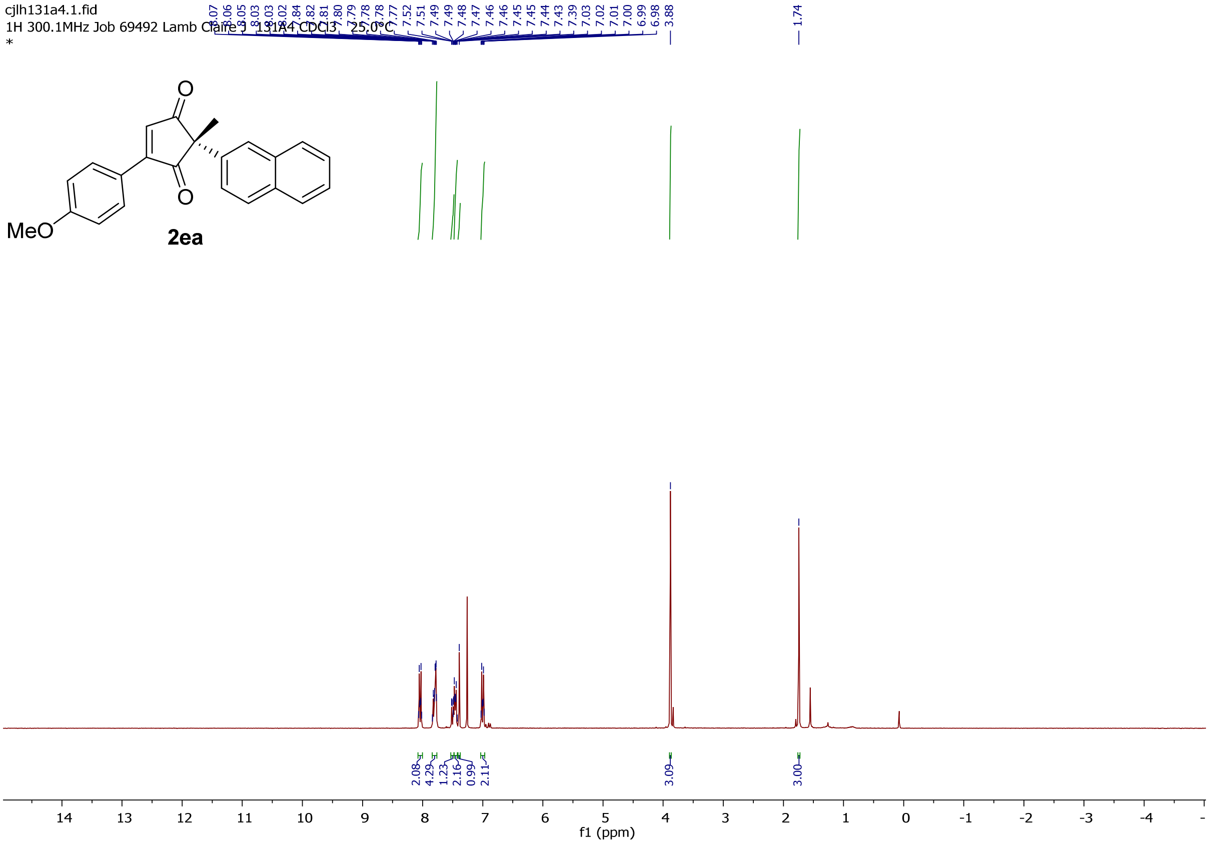
cjlh131a4.1.fid
1H 300.1MHz Job 69492 Lamb Claire J 131A4 CDCl3 25.0°C
*
8.07
8.06
8.05
8.03
8.03
8.02
7.84
7.82
7.81
7.80
7.79
7.78
7.78
7.77
7.52
7.51
7.49
7.49
7.48
7.47
7.46
7.46
7.45
7.45
7.44
7.43
7.39
7.03
7.02
7.01
7.00
6.99
6.98
3.88
1.74
MeO
O
O
2ea
2.08
4.29
1.23
2.16
0.99
2.11
3.09
3.00
f1 (ppm)

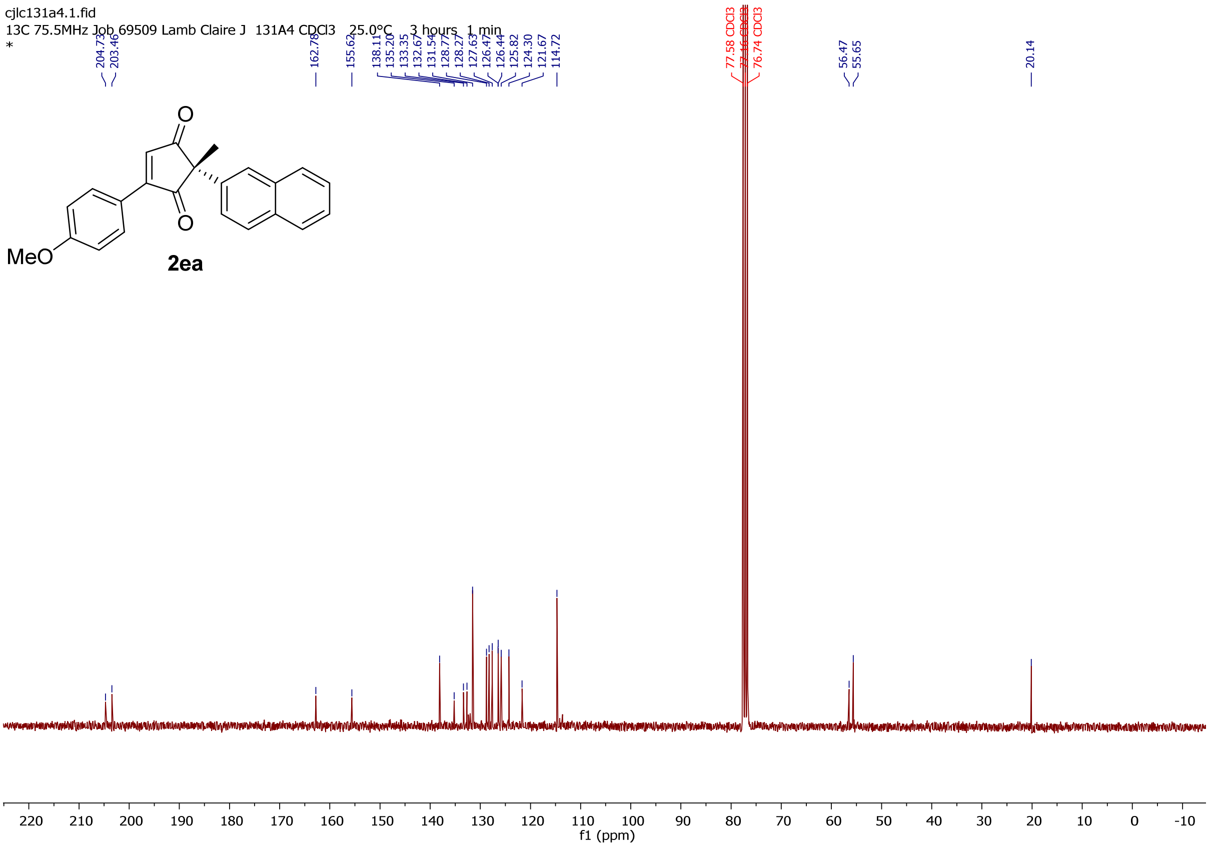
cjlc131a4.1.fid
13C 75.5MHz Job 69509 Lamb Claire J 131A4 CDCl3 25.0°C 3 hours 1 min
*
204.73
203.46
162.78
155.62
138.11
135.20
133.35
132.67
131.54
128.77
128.27
127.63
126.47
126.44
125.82
124.30
121.67
114.72
77.58 CDCl3
76.74 CDCl3
56.47
55.65
20.14
MeO
O
O
2ea
f1 (ppm)

References:

[1] T. Diao, S. S. Stahl, *J. Am. Chem. Soc.* **2011**, *133*, 14566-14569.

[2] K. Aikawa, T. Okamoto, K. Mikami, *J. Am. Chem. Soc.* **2012**, *134*, 10329.

[3] S. E. Walker, C. J. C. Lamb, N. A. Beattie, P. Nikodemiak, A.-L. Lee, *Chem. Commun.* **2015**, *51*, 4089.
